# Synthesis and biological evaluation of 4-phenoxy-phenyl isoxazoles as novel acetyl-CoA carboxylase inhibitors

**DOI:** 10.1080/14756366.2021.1936514

**Published:** 2021-06-08

**Authors:** Xin Wu, Yongbo Yu, Tonghui Huang

**Affiliations:** Jiangsu Key Laboratory of New Drug Research and Clinical Pharmacy, School of Pharmacy, Xuzhou Medical University, Xuzhou, China

**Keywords:** Acetyl-CoA carboxylase, antitumor, docking, cell cycle, apoptosis

## Abstract

Acetyl-CoA carboxylase (ACC) is a crucial enzyme in fatty acid metabolism, which plays a major role in the occurrence and development of certain tumours. Herein, one potential ACC inhibitor (**6a**) was identified through high-throughput virtual screening (HTVS), and a series of 4-phenoxy-phenyl isoxazoles were synthesised for structure-activity relationship (SAR) studies. Among these compounds, **6g** exhibited the most potent ACC inhibitory activity (IC_50_=99.8 nM), which was comparable to that of CP-640186. Moreover, the antiproliferation assay revealed that compound **6l** exhibited the strongest cytotoxicity, with IC_50_ values of 0.22 µM (A549), 0.26 µM (HepG2), and 0.21 µM (MDA-MB-231), respectively. The preliminary mechanistic studies on **6g** and **6l** suggested that the compounds decreased the malonyl-CoA levels, arrested the cell cycle at the G0/G1 phase, and induced apoptosis in MDA-MB-231 cells. Overall, these results indicated that the 4-phenoxy-phenyl isoxazoles are potential for further study in cancer therapeutics as ACC inhibitors.

## 1. Introduction

Fatty acids are one of the essential biological macromolecules for cell growth and survival, which are widely involved in energy supply, membrane biosynthesis, and signal transduction^1^^,^^2^. In contrast to normal cells that preferentially use circulating lipids to satisfy their requirement for fatty acids, malignant cells primarily undergo high rates of *de novo* fatty acid synthesis (FASyn) to support their continuous proliferation and division^3^^,^^4^. Therefore, elevated FASyn is accepted as a major characteristic of numerous cancers, including those in the prostate, colon, breast, lung, and liver. The drugs that intervene with the enzymes in FASyn may provide an innovative approach for the fight against cancer^5–8^.

Acetyl-CoA carboxylase (ACC), a crucial enzyme in FASyn, has two subcellular specific isoforms, namely ACC1 and ACC2, which catalyse the carboxylation of acetyl-CoA to malonyl-CoA and display distinct physiological roles^9^. ACC1 is a cytosolic enzyme that mainly controls the FASyn process, with its product malonyl-CoA extends the fatty acids chain by two carbon increments under the catalysis of fatty acid synthase^10^. In contrast, the mitochondrial isoform ACC2 is primarily responsible for fatty acid oxidation (FAOxn) through the inhibition of carnitine palmitoyltransferase I (CPT-1) by localised malonyl-CoA production^11^. Thus, the functional abnormalities on ACC could provide a viable modality for disturbing the energy metabolism and causing cell damage, which facilitates the utilisation of ACC as an attractive therapeutic target^12^^,^^13^. Currently, cancer therapeutics mainly focuses on the ACC1 isoform due to the over-expression of ACC1 mRNA in most human cancers^14–18^.

**Figure 1.**
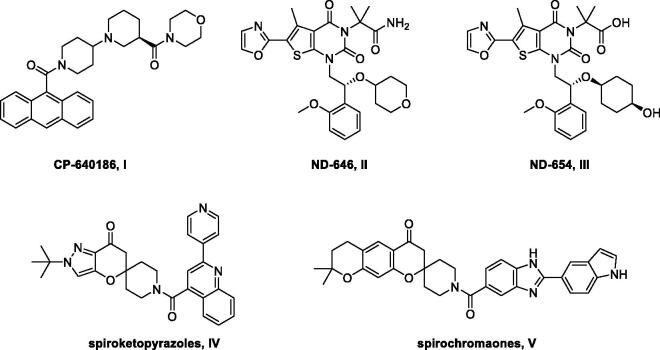
CP-640186 and representative ACC inhibitors with anticancer activity.

**Figure 2.**
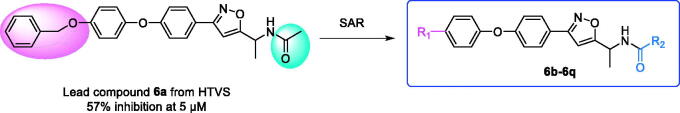
The design strategy of 4-phenoxy-phenyl isoxazoles as novel ACC inhibitors.

Since the first discovery of the mammalian ACC inhibitor CP-640186 in 2003 (Figure 1), a large amount of ACC inhibitors have been identified, some of which have even been approved in clinical trials for the treatment of glycolipid metabolic diseases, such as type 2 diabetes mellitus (T2DM) and non-alcoholic steatohepatitis (NASH)^19–21^. However, ACC inhibitors with effective anticancer activities were rarely reported. And most of these inhibitors contain thienopyrimidinone or spirocyclic scaffolds, among which the spiroketopyrazoles (IV) were previously identified by the author to display potent ACC1 inhibitory activity and cytotoxicity^22–26^. Therefore, an investigation into the generation of novel ACC inhibitors was conducted to further expand our research on antitumor agents and increase the diversity of ACC inhibitors. During the initial stage of this work, compound **6a** (Figure 2), an initial high-throughput virtual screening (HTVS) hit with moderate ACC1 inhibitory potency (57% inhibition at 5 µM), was selected as a lead compound. Later, the structure–activity relationship (SAR) study of 4-phenoxy-phenyl isoxazoles was performed with the belief that these new chemical entities would effectively target the ACC enzyme and provide a new approach for cancer treatment.

Herein, starting from the HTVS hit **6a**, a series of 4-phenoxy-phenyl isoxazole derivatives were identified. The *p*-benzyloxy group of **6a** was substituted with various alkoxy groups to reduce the structural rigidity and increase the flexibility of target compounds. Furthermore, diverse amide and urea substituents were introduced to improve lipophilicity and increase the number of hydrogen bond donors. All of the compounds were evaluated for biological activity towards ACC1 enzyme and three different cancer cell lines (A549, HepG2, and MDA-MB-231). The preliminary docking and pharmacological studies of the representative compounds were carried out to elucidate the possible action mechanism of 4-phenoxy-phenyl isoxazoles.

## 2. Results and discussion

### 2.1. High-throughput virtual screening

To identify potent ACC inhibitors with new structural skeletons, 1,500,000 commercially available compounds from the ChemDiv database were docked into the active site of ACC in silico^27^^,^^28^. However, the co-crystal structure of ACC inhibitor and hACC1 has not been resolved. Therefore, we selected the co-crystal structure of CP-640186 and hACC2 (PDB ID: 3FF6) for HTVS due to the high degree of identity between ACC1 and ACC2 at the nucleotide level (60%) and the amino acid level (75%). Based on the docking results, 500 top-ranked compounds with high total scores were selected for the second round of screening, which mainly focussed on the binding modes and interactions between the candidates and the active site^28^. Finally, nine potential hits with new skeletons were obtained. Their corresponding properties are summarised in Table 1 (structures are provided in Figure S1). Thereafter, the selected compounds were purchased from the commercial vendors and evaluated for their ACC inhibitory activity using a luminescent ADP detection assay^24^. As shown in Table 1, compound **6a** emerged as a top candidate, exhibiting the strongest inhibition rate at 5 µM. Thus, compound **6a** was selected as a lead compound, and its benzyloxy and acetyl groups were modified for SAR studies (Figure 2).

**Table 1.** The screening results and biological activity of selected candidates.

Compounds	Total score^a^	Crash^a^	Polar^a^	hACC1 IRs (%)^b^
**C450-0875**	7.37	−1.58	1.82	13.53
**C522-2022**	9.71	−1.21	4.04	2.84
**D074-0032**	7.99	−4.43	2.70	14.98
**D098-1432 (6a)**	8.07	−1.25	2.08	57.39
**D400-1402**	6.41	−1.13	0.35	3.44
**D400-3199**	6.77	−1.05	0.99	6.93
**E667-1377**	8.84	−1.91	4.50	12.56
**F292-0452**	7.77	−0.95	2.43	6.98
**F697-0546**	9.04	−0.81	2.62	20.53
**CP-640186**	7.18	−1.05	2.08	86.83

^a^Total score, crash, and polar values were calculated using Sybyl-X 2.1 software.

^b^Inhibition rates (IRs) of compounds for hACC1 enzyme at 5 μM.

### 2.2. Chemistry

**Scheme 1.**
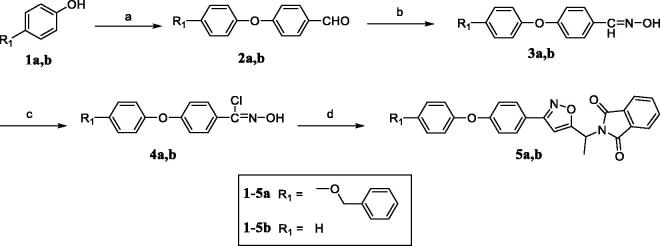
Synthetic route of key intermediate **5a** and **5b**. Reagents and conditions: (a) 4-fluorobenzaldehyde, K_2_CO_3_, DMF, 120 °C, 12 h; (b) hydroxylamine hydrochloride, NaOH, H_2_O/EtOH (3:1), 0 °C to r.t., 2 h; (c) NCS, DMF, r.t., 2 h; (d) 2-(but-3-yn-2-yl)isoindoline-1,3-dione, K_2_CO_3_, toluene, reflux, 12 h.

**Scheme 2.**
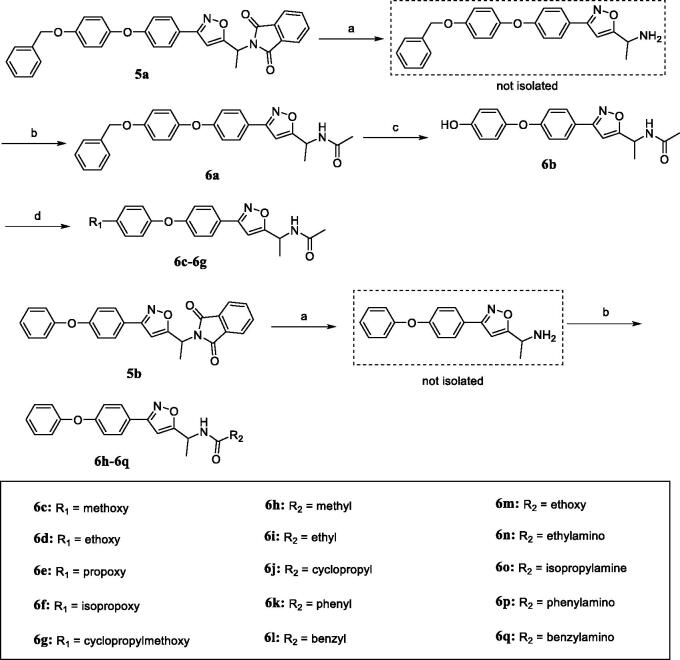
Synthetic route of target compounds **6a–6q**. Reagents and conditions: (a) hydrazine hydrate, CH_2_Cl_2_/EtOH (5:1), reflux, 2 h; (b) appropriate acyl chloride or isocyanate, TEA, CH_2_Cl_2_, 0 °C to r.t., 6 h; (c) H_2_, Pd-C, MeOH, r.t., 3 h; (d) appropriate iodoalkane, Cs_2_CO_3_, DMF, reflux, 12 h.

The 4-phenoxy-phenyl isoxazole derivatives (**6a–6q**) were synthesised according to the pathways described in Schemes 1 and 2. To brief, the commercially available 4-fluorobenzaldehyde was reacted with **1** (phenol or 4-(benzyloxy)phenol) in the presence of K_2_CO_3_ as the base at 120 °C to afford intermediate **2**. Then, the addition–elimination reactions of **2** and hydroxylamine readily generated the aldoxime **3**, which was subsequently chlorinated with N-chlorosuccinimide (NCS) to give the corresponding chloroaldoxime **4**. The [3 + 2] dipolar cycloaddition of **4** and 2-(but-3-yn-2-yl)isoindoline-1,3-dione provided the key intermediate **5** in good yields (**5a**, 86%; **5b**, 80%).

The phthalimide protecting group of **5a** was removed by the Ing-Manske reaction, and the resulting primary amine was condensed with acetyl chloride to afford the final compound **6a**. Later, compound **6b** was generated via Pd/C-catalysed hydrogenolysis. Moreover, the nucleophilic substitution of **6b** and appropriate iodoalkane using caesium carbonate as catalyst gave the target compounds **6c–6g**. The synthetic routes of the final compounds **6h–6q** were similar to that of **6a**, namely by the reaction of deprotected **5b** with various acyl chlorides or isocyanates.

### 2.3. Biological evaluation

#### 2.3.1. *In vitro* ACC inhibitory activity

To explore the SAR of 4-phenoxy-phenyl isoxazoles, the *in vitro* enzymatic inhibitory effects of **6a–6q** were evaluated. The preliminary screening was carried out at two concentrations (5 µM and 0.1 µM), where CP-640186 was selected as the positive control^29^. As shown in Table 2, the derivatives **6b–6d** with short-chain alkoxy groups at R_1_ exhibited weaker hACC1 inhibitory activity than **6a**. In contrast, the propoxy-substituted **6e** displayed 78.95 and 31.12% inhibition towards hACC1 at 5 and 0.1 µM, respectively, which indicated that the increase of the chain length is beneficial to the inhibition of ACC. Among the analogues with different substituents at R_1_ (**6a–6h**), compound **6g** appeared to be the most potential ACC inhibitor, with inhibition rates (IRs) of 95.26 and 51.37% at 5 µM and 0.1 µM, respectively.

**Table 2.** The hACC1 inhibitory activity and lipophilicity of compounds **6a–6q**.

Compounds	IRs (%)^a^		IC_50_ (nM)^b^	cLogP^c^
	5 μM	0.1 μM		
**6a**	54.93	18.13	NT	5.27
**6b**	26.83	8.76	NT	3.31
**6c**	37.32	9.89	NT	3.66
**6d**	45.46	12.73	NT	4.14
**6e**	78.95	31.12	438.2 ± 16.3	4.62
**6f**	52.76	16.69	NT	4.40
**6g**	95.26	51.37	99.8 ± 3.7	4.74
**6h**	36.37	8.78	NT	3.57
**6i**	51.83	18.51	NT	4.07
**6j**	58.36	20.79	NT	4.13
**6k**	72.47	28.11	626.3 ± 41.6	5.00
**6l**	81.42	37.86	279.1 ± 13.1	5.08
**6m**	16.77	3.57	NT	4.27
**6n**	44.72	13.36	NT	4.02
**6o**	49.82	17.90	NT	4.36
**6p**	65.73	21.52	1055.0 ± 179.7	5.21
**6q**	73.68	28.27	711.1 ± 52.6	5.18
**CP-640186**	87.84	48.87	108.9 ± 6.2	3.59

^a^The data represent the mean values of two independent experiments.

^b^IC_50_ values for ACC are presented as mean ± SD. NT: not test.

^b^cLogP values were calculated by Molsoft online software.

For 4-phenoxy-phenyl isoxazoles with various amide substituents at R_2_ (**6h**–**6l**), the analogues bearing aromatic ring showed better enzymatic activity, wherein compound **6l** exhibited the strongest ACC inhibitory effect (IRs = 81.42 and 37.86%), which could be partly attributed to its bulky phenylacetamide group. Besides, the IRs of compound **6q** with the benzylurea group (IRs = 73.68 and 28.27%) were the highest among the ureido-substituted derivatives (**6n–6q**), although slightly lower than that of the corresponding amide derivative **6l**. The inhibitory activity of the carbamate-substituted compound **6m** was hardly observed. These results suggested that the introduction of flexible alkoxy groups (such as propoxy and cyclopropylmethoxy, **6e** and **6g**) at the left-hand hydrophobic terminus, or the incorporation of bulky functional groups (such as phenylacetamide and benzylurea, **6l** and **6q**) at the right-hand polar end of the molecule was conducive to the inhibition of ACC.

**Figure 3.**
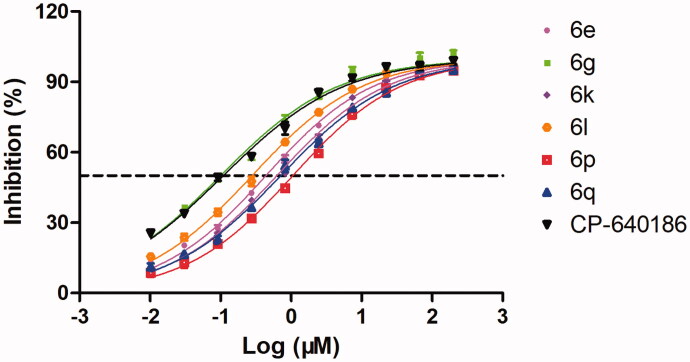
The dose–response curves of selected compounds on hACC1 enzyme.

Based on the above results, the IC_50_ values of the preferred compounds (**6e**, **6g**, **6k**, **6l**, **6p** and **6q**) with IRs greater than 60% at 5 µM were calculated and summarised in Table 2. Similar to the preliminary screening results, the cyclopropylmethoxy-substituted derivative **6g** exhibited the optimal ACC inhibitory activity among the synthesised compounds. The IC_50_ value of **6g** on the hACC1 enzyme was 99.8 nM, which was lower than that of CP-640186 (IC_50_=108.9 nM). The dose-response curves of the selected compounds were plotted, as presented in Figure 3.

#### 2.3.2. *In vitro* cytotoxic activity

The cytotoxicity of **6a**–**6q** on three different cancer cell lines with a high expression of ACC1 (A549, HepG2, and MDA-MB-231) was determined using the MTT assay^24^. The initial screening was performed at a single concentration of 10 µM, where doxorubicin (DOX) was served as a reference drug. As illustrated in Figure S2, the 4-phenoxyphenylisoxazoles showed the best antiproliferative activity on MDA-MB-231 cells, followed by A549 cells, and the weakest on HepG2 cells. Besides, most of the target compounds displayed significant inhibitory effects on cell proliferation. Among them, compounds **6a**, **6g**, and **6l** exhibited inhibitory rates greater than 80% against the three cancer cell lines, which were comparable to that of DOX.

Based on the good initial screening results, the IC_50_ values and calculated logarithm of the octanol-water partition coefficient (cLogp) of 4-phenoxy-phenyl isoxazoles were determined for further SAR studies^30^. As shown in Table 3, when *R*_2_ was fixed as a methyl group, the derivatives **6a**–**6h** exhibited varying degrees of antiproliferative activity. Among them, the compounds **6e** and **6g** with good ACC inhibitory activities exhibited satisfactory antitumor effects, in which **6g** displayed the best inhibition on A549, HepG2, and MDA-MB-231 cells, with IC_50_ values of 1.10, 1.73, and 1.50 µM, respectively. Besides, compound **6a** with moderate ACC activity and high lipophilicity showed considerable antiproliferative activity on the three cancer cell lines. However, the cytotoxicity of the derivatives (**6b**–**6d**, **6h**) with relatively weak enzymatic activity and low lipophilicity decreased remarkably (IC_50_ >10 µM).

**Table 3.** *In vitro* anti-proliferative activity of compounds **6a–6q**.

Compounds	IC_50_ (μM)^a^			Cell viability (%) of HUVEC at 10 μM^b^
	A549	HepG2	MDA-MB-231	
**6a**	1.40 ± 0.22	1.91 ± 0.66	1.57 ± 0.09	45.79 ± 1.16
**6b**	>10	>10	>10	88.14 ± 3.83
**6c**	>10	>10	>10	82.39 ± 4.85
**6d**	>10	>10	>10	89.06 ± 6.09
**6e**	2.66 ± 0.47	3.48 ± 0.74	1.53 ± 0.38	83.68 ± 4.40
**6f**	>10	>10	6.77 ± 0.74	34.96 ± 1.66
**6g**	1.10 ± 0.12	1.73 ± 0.55	1.50 ± 0.58	87.33 ± 6.19
**6h**	>10	>10	>10	91.53 ± 2.58
**6i**	6.02 ± 0.63	4.21 ± 0.53	2.97 ± 1.49	70.33 ± 0.25
**6j**	2.66 ± 0.47	3.48 ± 0.74	1.13 ± 0.38	59.46 ± 0.95
**6k**	2.61 ± 0.34	3.23 ± 0.89	0.57 ± 0.18	80.00 ± 2.47
**6l**	0.22 ± 0.07	0.26 ± 0.02	0.21 ± 0.01	89.54 ± 3.21
**6m**	>10	>10	>10	79.12 ± 4.76
**6n**	5.78 ± 0.86	9.04 ± 1.43	8.63 ± 1.14	83.23 ± 2.33
**6o**	3.12 ± 0.19	2.31 ± 0.44	1.90 ± 0.52	77.65 ± 5.67
**6p**	5.30 ± 0.46	1.80 ± 0.62	0.84 ± 0.08	85.60 ± 7.35
**6q**	5.73 ± 0.11	2.47 ± 0.50	0.89 ± 0.26	84.92 ± 3.98
**DOX**	0.52 ± 0.07	0.67 ± 0.13	0.42 ± 0.09	18.95 ± 4.02

^a^IC_50_ values for A549, HepG2, and MDA-MB-231 cells are presented as mean ± SD (*n* = 3).

^b^Cell viability was evaluated after incubation of compounds at a concentration of 10 μM.

On the other hand, when *R*_1_ was fixed as a hydrogen atom (**6h**–**6q**), most of the synthesised compounds exhibited potent *in vitro* antiproliferative activity. Of the five amide derivatives (**6h–6l**), compound **6l** showed the best potency in cancer treatment, with IC_50_ values of 0.22, 0.26, and 0.21 µM for A549, HepG2, and MDA-MB-231 cells, respectively, which were apparently superior to DOX. The outstanding cell activity of **6l** was supposed to be attributed to its optimal balance between the enzymatic activity and lipophilicity. Besides, the ureido-substituted compounds (**6n**–**6q**) exhibited remarkable performances towards the inhibition of MDA-MB-231 cells. The IC_50_ values of **6p** (0.84 µM) and **6q** (0.89 µM) against MDA-MB-231 cells were both less than 1 µM. In contrast, the carbamate-substituted **6m** only provided a faint antitumor effect, suggesting that the carbamate group was detrimental to bioactivity.

To investigate the selectivity of the synthesised compounds towards cancer cells, the non-cancerous HUVEC cell line was selected for testing^24^. As shown in Table 2, most of the compounds exhibited weak toxic to HUVEC. Moreover, the compounds with strong ACC inhibitory and antitumor activities, namely **6e**, **6g**, **6k**, **6l**, **6p** and **6q**, showed less than 20% IRs towards HUVEC, which indicated the cancer cell selectivity of ACC inhibitors. Overall, the SAR studies revealed that the ACC inhibitory activity and lipophilicity of the target compounds play an important role in their antiproliferative activity.

#### 2.3.3. Effect on intracellular malonyl-CoA levels

**Figure 4.**
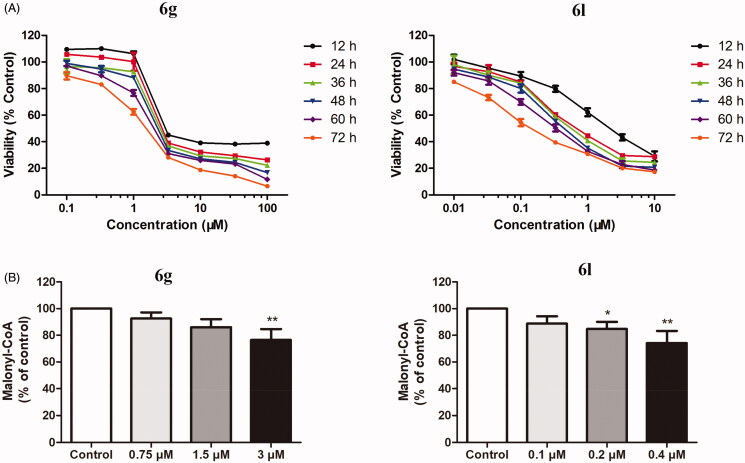
(A) Concentration- and time-dependent cytotoxicity of compounds **6g** and **6l**. The MDA-MB-231 cells were treated with drugs at increasing concentrations for 12, 24, 36, 48, 60, and 72 h, respectively. (B) Effects of **6g** and **6l** on the levels of malonyl-CoA in MDA-MB-231 cells. MDA-MB-231 cells were treated with diverse concentrations of drugs for 48 h, with the intracellular malonyl-CoA quantified. Data were expressed as mean ± SD (*n* = 3). *p < 0.05, **p < 0.01 versus control group.

Due to the most potent ACC1 inhibitory activity of **6g** and strongest antitumor activity of **6l**, these compounds were selected for further biological studies. Moreover, MDA-MB-231 was chosen as the model cell line due to the best antiproliferative effect of 4-phenoxy-phenyl isoxazoles on these cells. As illustrated in Figure 4(A), compounds **6g** and **6l** inhibited the proliferation of MDA-MB-231 cells in a concentration- and time-dependent manner. Later, the effects of **6g** and **6l** on the levels of malonyl-CoA in MDA-MB-231 cells were investigated. The results suggested that the treatment of the cells with **6g** and **6l** significantly decreased the intracellular malonyl-CoA levels, with an approximate reduction of 23.59 and 25.85% compared to the control group at the maximum test concentration, respectively (Figure 4(B)).

#### 2.3.4. Cell-cycle assay

**Figure 5.**
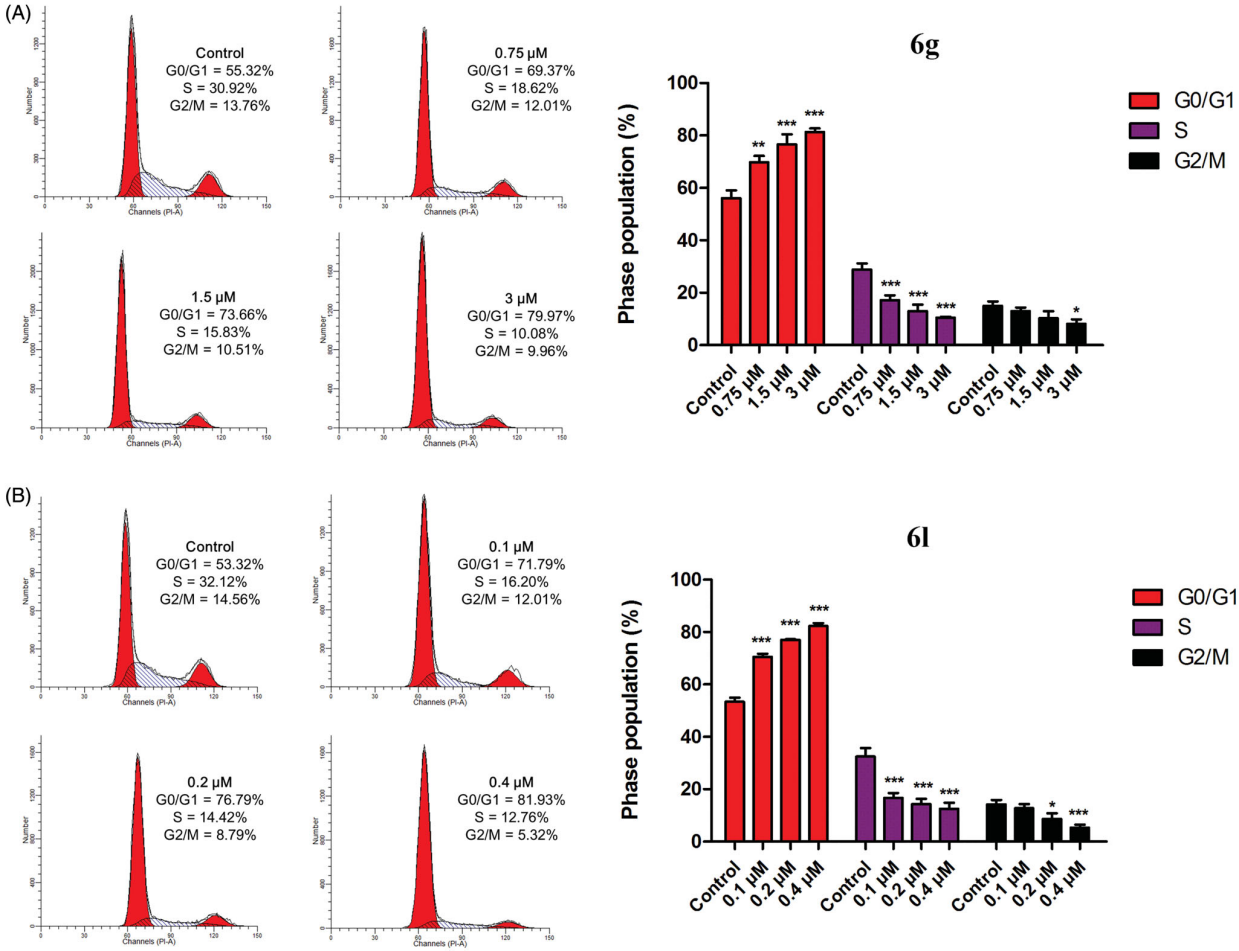
The effect of compounds **6g** (A) and **6l** (B) on cell-cycle distribution. MDA-MB-231 cells were treated with **6g** and **6l** at the indicated concentrations for 48 h, and stained with propidium iodide for flow cytometry. Data are expressed as mean ± SD (*n* = 3). *p < 0.05, **p < 0.01, and ***p < 0.001 versus control group.

The cell-cycle arrest creates a stopping point at which the cells no longer participate in the process of duplication and division that represents an important approach for cancer treatment^31^^,^^32^. To investigate the cell-cycle progression, a flow cytometric analysis was performed on the MDA-MB-231 cells treated with various concentrations (1/2 IC_50_, IC_50_, and 2 × IC_50_) of **6g** and **6l** for 48 h. As shown in Figure 5, compound **6g** led to an obvious increase in the cells at the G0/G1 phase from 55.32 to 79.97%, accompanied by a decrease in the cells at the S and G2/M phases from 30.93 and 13.76% to 10.08 and 9.96%, respectively. Moreover, the treatment of the MDA-MB-231 cells with increasing concentrations of **6l** (0.1, 0.2, and 0.4 µM) increased the proportion of cells at the G0/G1 phase from 53.32% to 81.93%, while the proportion of cells at the S and G2/M phases decreased. These results confirmed that **6g** and **6l** induced cell-cycle arrest at the G0/G1 phase in a dose-dependent manner, which may be a possible mechanism for their antitumor activity.

#### 2.3.5. Cell apoptosis assay

**Figure 6.**
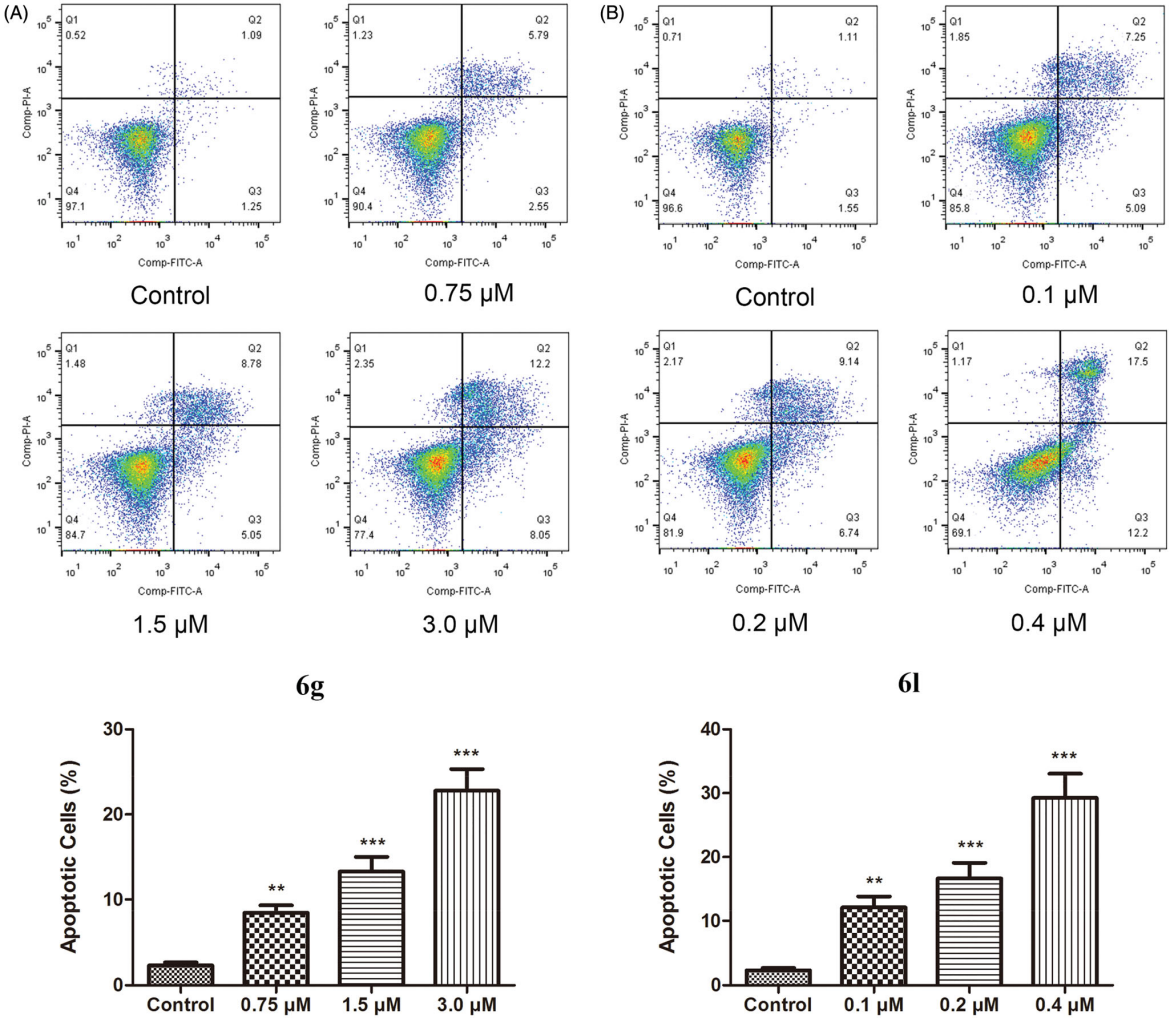
The effect of compounds **6g** (A) and **6l** (B) on cell apoptosis. MDA-MB-231 cells were incubated with **6g** and **6l** at the indicated concentrations for 48 h, and stained with Annexin V-FITC/PI for flow cytometry. Data are expressed as mean ± SD (*n* = 3). **p < 0.01 and ***p < 0.001 versus control group.

Apoptosis is a form of programmed cell death, which plays an important role in many cancer treatment strategies^33^^,^^34^. To explore the relationship between the antiproliferative effects of 4-phenoxy-phenyl isoxazoles and cell apoptosis, Annexin V and propidium iodide (PI) double staining was performed on the vehicle- and drug-treated MDA-MB-231 cells. Upon incubation with compound **6g** (0.75, 1.5, and 3 µM) for 48 h, the total percentage of the early (bottom right quadrant) and late (upper right quadrant) apoptotic cells was increased from 2.34 to 8.34, 13.83, and 20.25%, respectively, as depicted in Figure 6. In addition, compound **6l** induced early (from 1.55 to 12.20%) and late apoptosis (from 1.11 to 17.50%) in a dose-dependent manner. These data suggested that the antitumor activity of compounds **6g** and **6l** might be involved in the induction of apoptosis.

### 2.4. Molecular docking

**Figure 7.**
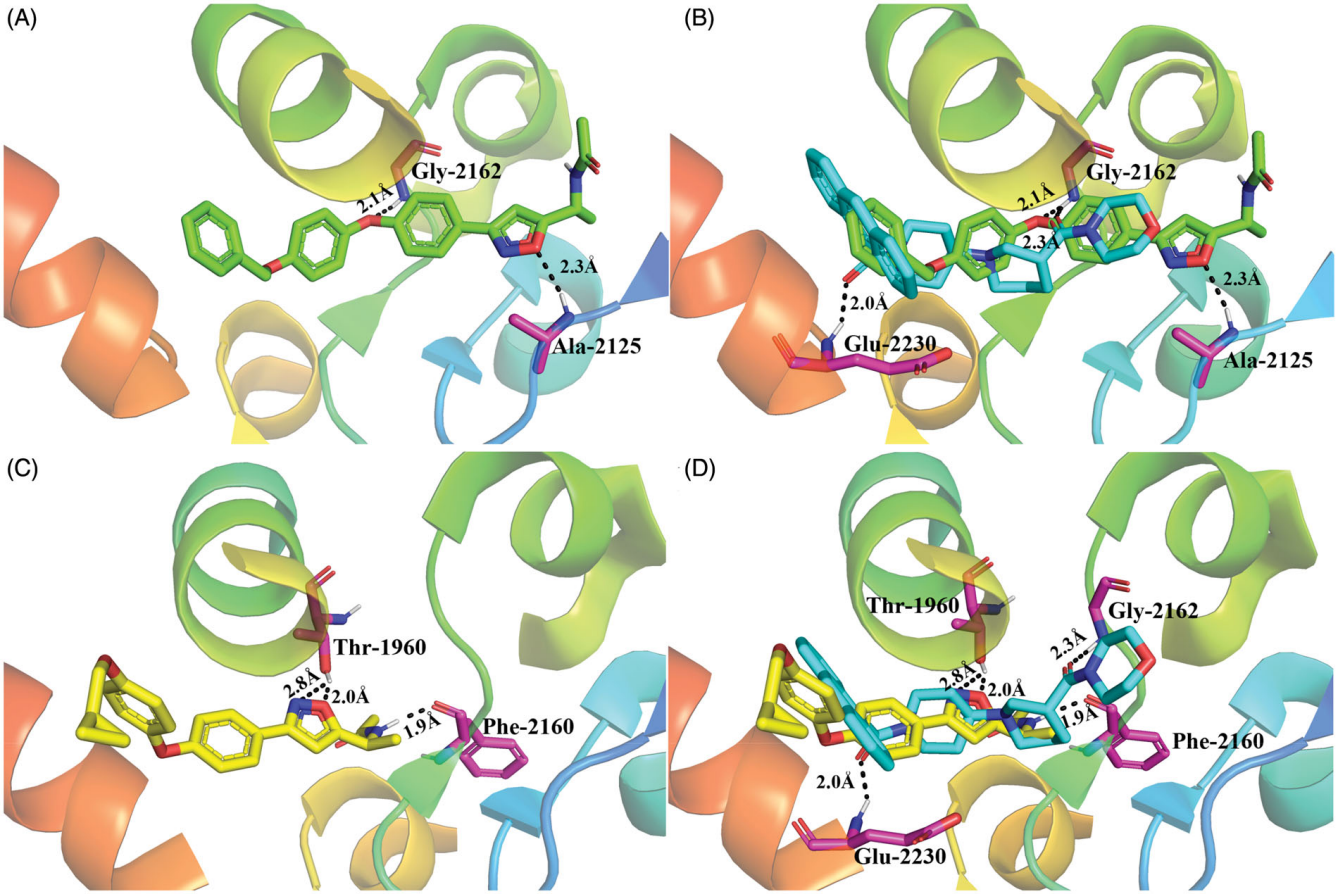
Molecular docking studies for compounds **6a** and **6g** (PDB ID: 3FF6). (A) Docking model of compound **6a** (green) with the active site of ACC. (B) Overlapping conformation of **6a** (green) with CP-640186 (cyan). (C) Docking model of compound **6g** (yellow) with the active site of ACC. (D) Overlapping conformation of **6g** (yellow) with CP-640186 (cyan). All hydrogen bonds are shown with black dashed lines, and related amino acids are highlighted with magenta sticks.

To gain insight into the specific interactions between the target compounds and ACC enzyme, molecular docking studies of the representative compounds (**6a** and **6g**) were conducted using the co-crystal structure of ACC and CP-640186 (PDB ID: 3FF6) as the template^27^. As illustrated in Figure 7, compounds **6a** and **6g** were located at the dimer interface of the carboxyltransferase (CT) domain of ACC and exhibited different binding modes. Compared with CP-640186, **6a** formed one conserved hydrogen bond with Gly-2162 (2.1 Å), and one additional hydrogen bond between the oxygen of oxazole and Ala-2125 (2.3 Å) was observed (Figure 7(A,B)). However, there was no hydrogen bond between **6a** and Glu-2230, which partly explained its moderate ACC inhibitory activity. The substitution of a cyclopropylmethoxy group at R_1_ (**6g**) led to the movement of the binding site and formed three hydrogen bonds between **6g** and the CT domain (Figure 7(C)). To our surprise, **6g** and CP-640186 did not have the same hydrogen bond interaction with ACC (Figure 7(D)). In detail, the Thr-1960 residue formed two hydrogen bonds with the nitrogen (2.8 Å) and oxygen atoms (2.0 Å) of oxazole, and the Phe-2160 residue formed one hydrogen bond with the nitrogen of amide (1.9 Å). The stronger interaction of **6g** with ACC than that of **6a** might account for its better activity in enzyme inhibition.

## 3. Conclusions

In this study, a series of structurally novel 4-phenoxy-phenyl isoxazoles were synthesised and evaluated for their antiproliferative activities towards a panel of human cancer cell lines as ACC inhibitors. The lead compound **6a** was discovered from HTVS, and further SAR studies resulted in the identification of several derivatives with potent biological activity. Among them, compound **6g**, which formed three hydrogen bonds with the CT domain of ACC, exhibited the most potent hACC1 inhibitory activity (IC_50_=99.8 nM). Compound **6l** displayed the strongest antiproliferative activity, with IC_50_ values of 0.22, 0.26, and 0.21 µM against A549, HepG2, and MDA-MB-231 cells, respectively, which were superior to that of DOX. The best antitumor activity of compound **6l** might be attributed to its optimal balance between the enzymatic activity and lipophilicity. Furthermore, treatment of the MDA-MB-231 cells with **6g** and **6l** significantly decreased the intracellular malonyl-CoA levels. The flow cytometry analysis confirmed that **6g** and **6l** arrested cell cycle at the G0/G1 phase and induced apoptosis in a dose-dependent manner. Overall, these results indicated that the 4-phenoxy-phenyl isoxazoles exhibited potent anticancer activity as novel ACC inhibitors, which demonstrated the causal relationship between ACC inhibition and loss of cell viability.

## 4. Experimental

### 4.1. Chemistry

All reagents and solvents were purchased from commercial sources without further purification. The reaction progress was monitored by TLC on Silica Gel 60 F_254_ plates, and the target compounds were purified by column chromatography with silica gel (200–300 mesh size). Melting points were measured in open capillary tubes using YRT-3 melting point apparatus and were uncorrected. ^1^H NMR and ^13 ^C NMR spectra were recorded on JNM-ECZR 400 MHz spectrometer with tetramethyl silane (TMS) as an internal standard. High-resolution mass spectra (HRMS) were obtained from a (UHR-TOF) maXis 4 G instrument. Analytical HPLC was run on the Agilent 1260 HPLC instrument, equipped with Agilent SB-C18 column (Agilent Technologies, Palo Alto, CA) and UV detection at 250 nm. Eluent system was: 70% MeOH in H_2_O; flow rate = 0.2 ml/min.

#### 4.1.1. General procedure for the preparation of key intermediate 5a–b

To a solution of phenol or 4-benzyloxy phenol (**1**, 27.5 mmol) in DMF (25 ml), K_2_CO_3_ (50 mmol) and 4-fluorobenzaldehyde (27.5 mmol) was added. After stirring at 120 °C for 12 h, the mixture was poured into water (100 ml) and extracted with ethyl acetate (3 × 25 ml). The combined organics were dried over anhydrous Na_2_SO_4_ and concentrated to give **2**. Then, hydroxylamine hydrochloride (20 mmol) and 50% NaOH aqueous solution (40 mmol) was slowly added to a solution of **2** (20 mmol) in H_2_O/ethanol (3:1, 20 ml) at 0 °C. After stirring at room temperature for 2 h, the mixture was acidified to pH 6 by concentrated HCl and extracted with ethyl acetate (3 × 5 ml). The organic phases were dried over anhydrous Na_2_SO_4_ and concentrated *in vacuo* to afford **3**. To a solution of oxime **3** (20 mmol) in DMF (25 ml), *N*-chlorosuccinimide (22 mmol) was added slowly at ambient temperature, the solution was stirred for 2 h before being poured into ice water (100 ml). The resulting precipitate was collected by filtration and dried to provide **4**. Finally, 2-(But-3-yn-2-yl)isoindoline-1,3-dione (10 mmol), **4** (11 mmol), and K_2_CO_3_ (30 mmol) was dissolved in toluene (50 ml) and heated at reflux for 12 h. After cooling to room temperature, the mixture was filtered, concentrated, and purified by a silica gel flash column to give the key intermediate **5**.

##### 4.1.1.1. 2-(1-(3-(4-(4-(benzyloxy)phenoxy)phenyl)isoxazol-5-yl)ethyl)isoindoline-1,3-dione (5a)

White solid, yield 70%, m.p. 160.5–162.5 °C. ^1^H NMR (400 MHz, CDCl_3_): *δ* 7.87–7.85 (m, 2H), 7.77–7.67 (m, 4H), 7.49–7.31 (m, 5H), 7.06–6.92 (m, 6H), 6.55 (d, *J* = 0.6 Hz, 1H), 5.70 (q, *J* = 7.3 Hz, 1H), 5.06 (s, 2H), 1.93 (d, *J* = 7.3 Hz, 3H). ^13 ^C NMR (100 MHz, CDCl_3_): *δ* 170.6, 167.3, 161.9, 160.1, 155.4, 149.4, 136.8, 134.3, 131.7, 128.6, 128.3, 128.0, 127.5, 123.5, 122.9, 121.2, 117.5, 116.0, 100.3, 70.5, 42.3, 16.8. HRMS *m/z* calcd for C_32_H_24_N_2_O_5_ [*M* + Na]^+^ 539.1577, found 539.1577.

##### 4.1.1.2. 2-(1-(3-(4-Phenoxyphenyl)isoxazol-5-yl)ethyl)isoindoline-1,3-dione (5b)

White solid, yield 72%, m.p. 133.5–134.5 °C. ^1^H NMR (400 MHz, CDCl_3_): *δ* 7.87–7.84 (m, 2H), 7.78–7.72 (m, 4H), 7.40–7.33 (m, 2H), 7.15 (t, *J* = 7.4 Hz, 1H), 7.08–7.01 (m, 4H), 6.57 (s, 1H), 5.70 (q, *J* = 7.0 Hz, 1H), 1.94 (d, *J* = 7.3 Hz, 3H). ^13 ^C NMR (100 MHz, CDCl_3_): *δ* 170.7, 167.3, 161.9, 159.0, 156.3, 134.3, 131.7, 129.9, 128.4, 123.9, 123.6, 119.5, 118.6, 100.3, 42.3, 16.8. HRMS *m/z* calcd for C_26_H_24_N_2_O_4_ [*M* + Na]^+^ 433.1159, found 433.1167.

#### 4.1.2. General procedure for the preparation of target compounds 6a–6q

(A) Hydrazine monohydrate (8 mmol) was added dropwise to a solution of **5** (1.2 mmol) in methylene chloride/ethanol (5:1, 12 ml) and refluxed for 2 h. The mixture was extracted with methylene chloride (3 × 10 ml), dried over anhydrous Na_2_SO_4_, and concentrated *in vacuo* to give corresponding amines. The amine was dissolved in methylene chloride (8 ml) under an ice-bath. Then, triethylamine (3 mmol) and appropriate acyl chloride or isocyanate (1 mmol) were added sequentially at 0 °C, with the mixture stirred for 6 h. After removal of the solvent, the residue was purified by column chromatography to yield target compounds **6a** and **6h–6q**.

(B) To a solution of **6a** (10 mmol) in anhydrous methanol (60 ml), Pd-C catalyst (10%, 1.5 mmol) was added, and the mixture was hydrogenated for 3 h at atmospheric pressure. Upon completion of the reaction, the catalyst was removed by filtration and the solvent was evaporated to give **6b**.

(C) A mixture of **6b** (1 mmol), Cs_2_CO_3_ (2 mmol), and corresponding iodoalkane (1.2 mmol) in DMF (10 ml) was stirred at reflux for 12 h. After cooling to room temperature, the mixture was poured into ice water (50 ml) and extracted with methylene dichloride (3 × 10 ml). The combined organic phases were dried over anhydrous Na_2_SO_4_, filtered, and concentrated under reduced pressure. The residue was purified by column chromatography to afford target compounds **6c**–**6g**.

##### 4.1.2.1. N-(1-(3-(4-(4-(Benzyloxy)phenoxy)phenyl)isoxazol-5-yl)ethyl)acetamide (6a)

White solid, yield 70%, m.p. 152.5–153.5 °C. ^1^H NMR (400 MHz, DMSO-d_6_): *δ* 7.74–7.65 (m, 2H), 7.49–7.31 (m, 5H), 7.05–6.94 (m, 6H), 6.39 (s, 1H), 5.94 (d, *J* = 8.2 Hz, 1H), 5.44–5.33 (m, 1H), 5.06 (s, 2H), 2.03 (s, 3H), 1.57 (d, *J* = 7.1 Hz, 3H). ^13 ^C NMR (100 MHz, DMSO-d_6_): *δ* 173.0, 169.5, 162.0, 160.3, 155.6, 149.6, 137.0, 128.7, 128.4, 128.2, 127.6, 123.0, 121.4, 117.6, 116.1, 99.1, 70.6, 42.3, 23.3, 19.6. HRMS *m/z* calcd for C_26_H_24_N_2_O_4_ [*M* + Na]^+^ 451.1628, found 451.1630. Purity: 99.3%.

##### 4.1.2.2. N-(1-(3-(4-(4-Hydroxyphenoxy)phenyl)isoxazol-5-yl)ethyl)acetamide (6b)

White solid, yield 65%, m.p. 103–105 °C. ^1^H NMR (400 MHz, CDCl_3_): *δ* 7.70 (dd, *J* = 13.5, 8.3 Hz, 2H), 7.12–6.79 (m, 6H), 6.40 (s, 1H), 5.90 (d, *J* = 16.1 Hz, 1H), 5.46–5.31 (m, 1H), 2.05 (s, 3H), 1.59 (d, *J* = 5.9 Hz, 3H). ^13 ^C NMR (100 MHz, CDCl_3_): ^13 ^C NMR (101 MHz,) *δ* 172.7, 170.1, 162.1, 160.6, 153.2, 148.7, 128.4, 121.6, 117.9, 117.4, 116.6, 99.3, 42.4, 23.3, 19.5. HRMS *m/z* calcd for C_19_H_18_N_2_O_4_ [*M* + Na]^+^ 361.1159, found 361.1162. Purity: 99.2%.

##### 4.1.2.3. N-(1-(3-(4-(4-Methoxyphenoxy)phenyl)isoxazol-5-yl)ethyl)acetamide (6c)

White solid, yield 70%, m.p. 127.5–128.5 °C. ^1^H NMR (400 MHz, CDCl_3_): *δ* 7.81–7.60 (m, 2H), 7.03–6.91 (m, 6H), 6.40 (s, 1H), 5.97 (d, *J* = 6.2 Hz, 1H), 5.50–5.28 (m, 1H), 3.82 (s, 3H), 2.03 (s, 3H), 1.58 (d, *J* = 6.4 Hz, 3H). ^13 ^C NMR (100 MHz, CDCl_3_): *δ* 172.9, 169.4, 162.0, 160.4, 156.4, 149.3, 128.4, 121.4, 118.0, 117.5, 115.1, 99.1, 55.8, 42.2, 23.3, 19.6. HRMS *m/z* calcd for C_20_H_20_N_2_O_4_ [*M* + Na]^+^ 375.1315, found 375.1321. Purity: 98.4%.

##### 4.1.2.4. N-(1-(3-(4-(4-Ethoxyphenoxy)phenyl)isoxazol-5-yl)ethyl)acetamide (6d)

White solid, yield 72%, m.p. 106.5–107.5 °C. ^1^H NMR (400 MHz, CDCl_3_): *δ* 7.83–7.58 (m, 2H), 7.01–6.89 (m, 6H), 6.40 (s, 1H), 5.98 (d, *J* = 5.8 Hz, 1H), 5.50–5.25 (m, 1H), 4.07 (q, *J* = 12.0 Hz, 2H), 2.03 (s, 3H), 1.58 (d, *J* = 5.9 Hz, 3H), 1.43 (t, *J* = 6.3 Hz, 3H). ^13 ^C NMR (100 MHz, CDCl_3_): *δ* 172.9, 169.5, 162.1, 160.4, 155.8, 149.1, 128.4, 121.4, 118.0, 117.5, 115.7, 99.1, 64.0, 42.2, 23.3, 19.6, 15.0. HRMS *m/z* calcd for C_21_H_22_N_2_O_4_ [*M* + Na]^+^ 389.1472, found 389.1477. Purity: 97.4%.

##### 4.1.2.5. N-(1-(3-(4-(4-Propoxyphenoxy)phenyl)isoxazol-5-yl)ethyl)acetamide (6e)

White solid, yield 68%, m.p. 118.5–119.5 °C. ^1^H NMR (400 MHz, CDCl_3_): *δ* 7.71 (t, *J* = 8.2 Hz, 2H), 6.99 (t, *J* = 7.7 Hz, 4H), 6.91 (d, *J* = 8.8 Hz, 2H), 6.40 (s, 1H), 5.95 (d, *J* = 9.3 Hz, 1H), 5.47–5.28 (m, 1H), 3.92 (t, *J* = 4.5 Hz, 2H), 2.04 (s, 3H), 1.88–1.75 (m, 2H), 1.58 (d, *J* = 5.9 Hz, 3H), 1.06 (t, *J* = 6.2 Hz, 3H). ^13 ^C NMR (100 MHz, CDCl_3_): *δ* 172.9, 169.5, 162.1, 160.5, 156.0, 149.1, 128.4, 121.4, 117.9, 117.5, 115.7, 99.2, 70.1, 42.3, 23.3, 22.7, 19.6, 10.6. HRMS *m/z* calcd for C_22_H_24_N_2_O_4_ [*M* + Na]^+^ 403.1628, found 403.1633. Purity: 99.1%.

##### 4.1.2.6. N-(1-(3-(4-(4-Isopropoxyphenoxy)phenyl)isoxazol-5-yl)ethyl)acetamide (6f)

White solid, yield 65%, m.p. 124–126 °C. ^1^H NMR (400 MHz, CDCl_3_): *δ* 7.71 (t, *J* = 8.9 Hz, 2H), 7.07–6.81 (m, 6H), 6.40 (s, 1H), 5.95 (d, *J* = 6.8 Hz, 1H), 5.49–5.25 (m, 1H), 4.51 (s, 1H), 2.04 (s, 3H), 1.58 (d, *J* = 5.4 Hz, 3H), 1.37 (dd, *J* = 15.2, 5.6 Hz, 6H). ^13 ^C NMR (100 MHz, CDCl_3_): *δ* 172.9, 169.5, 162.1, 160.4, 154.7, 149.1, 128.4, 121.4, 118.1, 117.6, 117.3, 99.1, 70.6, 42.2, 23.3, 22.2, 19.6. HRMS *m/z* calcd for C_22_H_24_N_2_O_4_ [*M* + Na]^+^ 403.1628, found 403.1633. Purity: 98.8%.

##### 4.1.2.7. N-(1-(3-(4-(4-(Cyclopropylmethoxy)phenoxy)phenyl)isoxazol-5-yl)ethyl)acetamide (6g)

White solid, yield 50%, m.p. 131.5–132.5 °C. ^1^H NMR (400 MHz, CDCl_3_): *δ* 7.71 (t, *J* = 9.0 Hz, 2H), 7.14–6.81 (m, 6H), 6.40 (s, 1H), 5.89 (d, *J* = 8.4 Hz, 1H), 5.55–5.24 (m, 1H), 3.84 (dd, *J* = 33.2, 5.9 Hz, 2H), 2.04 (s, 3H), 1.58 (d, *J* = 6.7 Hz, 3H), 1.40–1.19 (m, 1H), 0.73–0.57 (m, 2H), 0.45–0.30 (m, 2H). ^13 ^C NMR (100 MHz, CDCl_3_): *δ* 172.9, 169.4, 162.0, 160.4, 155.9, 149.2, 128.4, 121.4, 118.0, 117.5, 115.84, 99.1, 73.4, 42.3, 23.3, 19.6, 10.4, 3.3. HRMS *m/z* calcd for C_23_H_24_N_2_O_4_ [*M* + Na]^+^ 415.1628, found 415.1634. Purity: 99.3%.

##### 4.1.2.8. N-(1-(3-(4-Phenoxyphenyl)isoxazol-5-yl)ethyl)acetamide (6h)

White solid, yield 85%, m.p. 118.5–119.5 °C. ^1^H NMR (400 MHz, CDCl_3_): *δ* 7.76–7.68 (m, 2H), 7.40–7.32 (m, 2H), 7.19–7.11 (m, 1H), 7.07–7.00 (m, 4H), 6.40 (s, 1H), 6.01 (d, *J* = 71.1 Hz, 1H), 5.45–5.31 (m, 1H), 2.03 (s, 3H), 1.57 (d, *J* = 7.1 Hz, 3H). ^13 ^C NMR (100 MHz, CDCl_3_): *δ* 173.0, 169.4, 161.9, 159.2, 156.3, 129.9, 128.4, 124.0, 123.5, 119.6, 118.6, 99.1, 42.2, 23.2, 19.5. HRMS *m/z* calcd for C_19_H_18_N_2_O_3_ [*M* + Na]^+^ 345.1210, found 345.1225. Purity: 96.9%.

##### 4.1.2.9. N-(1-(3-(4-Phenoxyphenyl)isoxazol-5-yl)ethyl)propionamide (6i)

White solid, yield 83%, m.p. 129.5–130.5 °C. ^1^H NMR (400 MHz, CDCl_3_): *δ* 7.78–7.68 (m, 2H), 7.40–7.36 (m, 2H), 7.21–7.12 (m, 1H), 7.09–7.00 (m, 4H), 6.41 (s, 1H), 5.87 (d, *J* = 7.8 Hz, 1H), 5.46–5.35 (m, 1H), 2.26 (q, *J* = 7.6 Hz, 2H), 1.59 (d, *J* = 7.1 Hz, 3H), 1.18 (t, *J* = 7.6 Hz, 3H). ^13 ^C NMR (100 MHz, CDCl_3_): *δ* 173.1, 173.1, 162.0, 159.3, 156.4, 130.0, 128.5, 124.1, 123.7, 119.6, 118.7, 99.1, 42.1, 29.6, 19.6, 9.7. HRMS *m/z* calcd for C_20_H_20_N_2_O_3_ [*M* + Na]^+^ 359.1366, found 359.1369. Purity: 98.2%.

##### 4.1.2.10. N-(1-(3-(4-Phenoxyphenyl)isoxazol-5-yl)ethyl)cyclopropanecarboxamide (6j)

White solid, yield 75%, m.p. 168.5–169.5 °C. ^1^H NMR (400 MHz, CDCl_3_): *δ* 7.79–7.70 (m, 2H), 7.44–7.33 (m, 2H), 7.18–7.14 (m, 1H), 7.10–7.01 (m, 4H), 6.41 (s, 1H), 6.09 (d, *J* = 8.3 Hz, 1H), 5.45–5.38 (m, 1H), 1.60 (d, *J* = 7.1 Hz, 3H), 1.43–1.32 (m, 1H), 1.06–0.94 (m, 2H), 0.81–0.77 (m, 2H). ^13 ^C NMR (100 MHz, CDCl_3_): *δ* 173.3, 162.0, 159.2, 156.4, 130.0, 128.5, 124.1, 123.7, 119.6, 118.7, 99.1, 42.3, 19.7, 14.8, 7.7. HRMS *m/z* calcd for C_21_H_20_N_2_O_3_ [*M* + Na]^+^ 371.1366, found 371.1367. Purity: 100%.

##### 4.1.2.11. N-(1-(3-(4-Phenoxyphenyl)isoxazol-5-yl)ethyl)benzamide (6k)

White solid, yield 72%, m.p. 155.5–156.5 °C. ^1^H NMR (400 MHz, CDCl_3_): *δ* 7.80 (dd, *J* = 5.3, 3.3 Hz, 2H), 7.76–7.71 (m, 2H), 7.56–7.50 (m, 1H), 7.48–7.42 (m, 2H), 7.40–7.34 (m, 2H), 7.19–7.12 (m, 1H), 7.07–7.03 (m, 4H), 6.52 (d, *J* = 7.7 Hz, 1H), 6.47 (s, 1H), 5.64–5.57 (m, 1H), 1.70 (d, *J* = 7.1 Hz, 3H). ^13 ^C NMR (100 MHz, CDCl_3_): *δ* 173.0, 166.8, 162.1, 159.3, 156.4, 133.8, 132.0, 130.0, 128.8, 128.5, 127.2, 124.1, 123.6, 119.6, 118.7, 99.3, 42.8, 19.7. HRMS *m/z* calcd for C_24_H_20_N_2_O_3_ [*M* + Na]^+^ 407.1366, found 407.1368. Purity: 99.0%.

##### 4.1.2.12. N-(1-(3-(4-Phenoxyphenyl)isoxazol-5-yl)ethyl)-2-phenylacetamide (6l)

White solid, yield 70%, m.p. 164.5–165.5 °C. ^1^H NMR (400 MHz, CDCl_3_): *δ* 7.70 (d, *J* = 8.6 Hz, 2H), 7.41–7.26 (m, 7H), 7.16 (t, *J* = 7.3 Hz, 1H), 7.05 (dd, *J* = 11.0, 4.4 Hz, 4H), 6.27 (s, 1H), 5.78 (d, *J* = 8.2 Hz, 1H), 5.41–5.33 (m, 1H), 3.62 (s, 2H), 1.50 (d, *J* = 7.0 Hz, 3H). ^13 ^C NMR (100 MHz, CDCl_3_): *δ* 172.9, 170.4, 161.9, 159.3, 156.4, 134.5, 130.0, 129.5, 129.2, 128.5, 127.7, 124.1, 123.6, 119.7, 118.7, 99.0, 43.8, 42.4, 19.4. HRMS *m/z* calcd for C_25_H_22_N_2_O_3_ [*M* + Na]^+^ 421.1523, found 421.1527. Purity: 96.7%.

##### 4.1.2.13. Ethyl (1-(3-(4-phenoxyphenyl)isoxazol-5-yl)ethyl)carbamate (6m)

White solid, yield 70%, m.p. 106.5–107.5 °C. ^1^H NMR (400 MHz, CDCl_3_): *δ* 7.74 (d, *J* = 7.5 Hz, 2H), 7.38 (dd, *J* = 9.2, 4.2 Hz, 2H), 7.29–7.23 (m, 1H), 7.16 (dd, *J* = 7.7, 6.8 Hz, 1H), 7.10–6.99 (m, 4H), 6.56–6.34 (m, 1H), 5.27–4.83 (m, 1H), 4.15 (q, *J* = 8.6 Hz, 2H), 1.59 (d, *J* = 3.0 Hz, 3H), 1.26 (t, *J* = 6.5 Hz, 3H). ^13 ^C NMR (100 MHz, CDCl_3_): *δ* 173.5, 161.9, 159.2, 156.4, 155.7, 130.0, 128.5, 124.1, 123.7, 119.6, 118.7, 98.9, 61.4, 44.2, 19.9, 14.6. HRMS *m/z* calcd for C_25_H_22_N_2_O_3_ [*M* + Na]^+^ 421.1523, found 421.1527. HRMS *m/z* calcd for C_20_H_20_N_2_O_4_ [*M* + Na]^+^ 375.1315, found 375.1320. Purity: 99.4%.

##### 4.1.2.14. 1-Ethyl-3-(1-(3-(4-phenoxyphenyl)isoxazol-5-yl)ethyl)urea (6n)

White solid, yield 80%, m.p. 158.5–159.5 °C. ^1^H NMR (400 MHz, DMSO-d_6_): *δ* 7.91–7.80 (m, 2H), 7.50–7.37 (m, 2H), 7.27–7.17 (m, 1H), 7.15–7.04 (m, 4H), 6.78 (s, 1H), 6.48 (d, *J* = 8.3 Hz, 1H), 5.87 (t, *J* = 5.6 Hz, 1H), 5.03–4.96 (m, 1H), 3.03 (q, *J* = 3.6 Hz, 2H), 1.42 (d, *J* = 7.1 Hz, 3H), 1.00 (t, *J* = 7.2 Hz, 3H). ^13 ^C NMR (100 MHz, DMSO-d_6_): *δ* 176.5, 161.6, 159.0, 157.5, 156.3, 130.8, 129.0, 124.7, 124.1, 120.0, 119.0, 99.0, 42.8, 34.7, 20.5, 16.2. HRMS *m/z* calcd for C_20_H_21_N_3_O_3_ [*M* + Na]^+^ 374.1475, found 374.1480. Purity: 98.6%.

##### 4.1.2.15. 1-Isopropyl-3-(1-(3-(4-phenoxyphenyl)isoxazol-5-yl)ethyl)urea (6o)

White solid, yield 75%, m.p. 197.5–198.5 °C. ^1^H NMR (400 MHz, DMSO-d_6_): *δ* 7.91–7.79 (m, 2H), 7.48–7.39 (m, 2H), 7.25–7.18 (m, 1H), 7.16–7.03 (m, 4H), 6.77 (s, 1H), 6.34 (d, *J* = 8.4 Hz, 1H), 5.75 (d, *J* = 7.7 Hz, 1H), 5.08–4.91 (m, 1H), 3.72–3.64 (m, 1H), 1.42 (d, *J* = 7.1 Hz, 3H), 1.04 (dd, *J* = 6.5, 1.8 Hz, 6H). ^13 ^C NMR (100 MHz, DMSO-d_6_): *δ* 176.4, 161.6, 159.0, 156.9, 156.3, 130.8, 129.0, 124.7, 124.1, 120.0, 119.0, 99.0, 42.7, 41.5, 23.7, 23.7, 20.6. HRMS *m/z* calcd for C_21_H_23_N_3_O_3_ [*M* + Na]^+^ 388.1632, found 388.1638. Purity: 100%.

##### 4.1.2.16. 1-(1-(3-(4-Phenoxyphenyl)isoxazol-5-yl)ethyl)-3-phenylurea (6p)

White solid, yield 73%, m.p. 195.5–196.5 °C. ^1^H NMR (400 MHz, DMSO-d_6_): *δ* 8.48 (s, 1H), 7.94–7.81 (m, 2H), 7.50–7.35 (m, 4H), 7.22 (dd, *J* = 16.1, 7.8 Hz, 3H), 7.14–7.05 (m, 4H), 6.96–6.86 (m, 2H), 6.80 (d, *J* = 8.1 Hz, 1H), 5.12–5.07 (m, 1H), 1.51 (d, *J* = 7.0 Hz, 3H). ^13 ^C NMR (100 MHz, DMSO-d_6_): *δ* 176.4, 161.6, 159.0, 156.9, 156.3, 130.8, 129.0, 124.7, 124.1, 120.0, 119.0, 99.0, 42.7, 41.5, 23.7, 23.7, 20.6. HRMS *m/z* calcd for C_24_H_21_N_3_O_3_ [*M* + Na]^+^ 422.1475, found 422.1480. Purity: 96.7%.

##### 4.1.2.17. 1-Benzyl-3-(1-(3-(4-phenoxyphenyl)isoxazol-5-yl)ethyl)urea (6q)

White solid, yield 76%, m.p. 161.5–162.5 °C. ^1^H NMR (400 MHz, DMSO-d_6_): *δ* 7.93–7.78 (m, 2H), 7.54–7.39 (m, 2H), 7.35–7.19 (m, 6H), 7.14–7.05 (m, 4H), 6.78 (s, 1H), 6.64 (d, *J* = 8.3 Hz, 1H), 6.42 (t, *J* = 6.0 Hz, 1H), 5.07–4.99 (m, 1H), 4.32–4.14 (m, 2H), 1.45 (d, *J* = 7.1 Hz, 3H). ^13 ^C NMR (100 MHz, DMSO-d_6_): *δ* 176.4, 161.6, 159.0, 157.7, 156.3, 141.2, 130.8, 129.0, 128.8, 127.6, 127.2, 124.7, 124.1, 120.0, 119.0, 99.1, 43.5, 43.0, 20.5. HRMS *m/z* calcd for C_25_H_23_N_3_O_3_ [*M* + Na]^+^ 436.1632, found 436.1636. Purity: 98.9%.

### 4.2. HTVS

The ChemDiv database was provided by Topscience Co., and the HTVS was performed using the Surflex docking module in Sybyl-X 2.1^28^. The crystal complex of ACC and CP-640186 (PDB ID: 3FF6) was downloaded from the Protein Data Bank. To improve the efficiency of screening, the maximum quantity of conformations was reduced from 20 to 10, and the maximum quantity of rotatable bonds was decreased from 100 to 50, with other parameters kept as usual. Compounds with high total-scores (top 500) were extracted to observe their binding modes and interactions with the CT domain. Finally, nine candidates with new scaffolds were obtained. The cLog*p* values and drug-likeness scores were calculated by Molsoft software (http://www.molsoft.com/mprop/).

### 4.3. ACC inhibitory assay

The hACC1 inhibitory activity of target compounds was evaluated using the ADP-Glo^TM^ kinase assay according to our previous report^24^. In brief, 4.5 µL of working solution containing recombinant ACC1 (BPS Biosciences, 50200) and 0.5 µL of diluted compound solution were added to a 384-well Optiplate plate (Perkin Elmer, 6007290, PerkinElmer, Waltham, MA, USA), and incubated at room temperature for 15 min. Then, 5 µL of substrate mixture was added to the 384-well plate to start the reaction. After incubating for 60 min at room temperature, 10 µL of ADP-Glo reagents were added to the 384-well plate, and incubated for another 40 min to deplete the remaining ATP. Finally, 20 µL of kinase detection reagents were added to the plate and incubated for 40 min to convert ADP to ATP. The luminescent signal of ATP was determined using an Envision multifunction reader (Perkin Elmer, 2104–0010, PerkinElmer, Waltham, MA, USA). The IC_50_ values were calculated using GraphPad Prism 5 software (GraphPad Software, La Jolla, CA, USA).

### 4.4. Molecular docking

The docking study was carried out with the Sybyl-X 2.1 software, and the crystal complex of ACC and CP-640186 (PDB ID: 3FF6) was downloaded from the Protein Data Bank. The whole waters and superfluous ligands were removed, and hydrogen atoms were added to the original protein structure. For the docked ligands, they were added with non-polar hydrogens and further energy was minimised with the Tripos force field. Then, the optimised ligands were docked to the prepared protein using a Surflex-Dock protocol. The optimal binding pose of the ligand was determined by the total score and its interaction with ACC. The best docking conformations of **6a** and **6g**, as well as their corresponding overlapping conformations with CP-640186 was visualised by PyMOL.

### 4.5. MTT assay

The cytotoxicity of target compounds was assessed by MTT assay. Briefly, cells were seeded in 96-well plates at a density of 2500–3000 per well and incubated for 24 h in a CO_2_ incubator. Then, the cells were treated with candidate compounds at seven different concentrations for 72 h. At the end of treatment, the MTT reagent (5 mg/mL) was added to determine the cell viability. After 4 h of incubation, the resulting medium was removed, and 100 µL of DMSO was added to dissolve the formed formazan. Finally, the absorbance at 550 nm was measured by a microplate reader (Varioskan LUX, Promega, Madison, WI), with the IC_50_ values calculated using GraphPad Prism 5 software (GraphPad Software, La Jolla, CA, USA).

### 4.6. Measurement of malonyl-CoA levels

MDA-MB-231 cells were seeded on 6-well plates and treated with various concentrations of **6g** and **6l** for 48 h. Cells were collected, washed with PBS, and lysed with 1% Triton X-100. The lysates were centrifuged at 4 °C for 15 min. The malonyl-CoA levels were determined using Elisa assay kits (Ruixin Biotech, Beijing, China).

### 4.7. Cell-cycle assay

MDA-MB-231 cells were seeded on 6-well plates (3 × 10^4^ cells/well) and incubated with indicated concentrations of **6g** (0.75, 1.5, and 3 µM) and **6l** (0.1, 0.2, and 0.4 µM) for 48 h in a 5% CO_2_ incubator. Then, the cells were collected by trypsinization and centrifugation, followed by fixing in 70% pre-cooled ethanol overnight. Finally, the cells were stained with PI/RNase A according to the manual and measured by flow cytometer (Facs Canto II, Becton Dickinson, San Jose, CA’, USA)^35^.

### 4.8. Cell apoptosis assay

MDA-MB-231 cells were plated on 6-well plates (3 × 10^4^ cells/well) and incubated with various concentrations of **6g** (0.75, 1.5, and 3 µM) and **6l** (0.1, 0.2, and 0.4 µM) for 48 h. Then, cells were harvested by trypsinization and centrifugation, followed by staining with annexin-V-FITC in binding buffer for 15 min protected from light. Subsequently, the cells were stained with PI, and the proportion of apoptotic cells was measured by flow cytometer (Facs Canto II, Becton Dickinson, San Jose, CA, USA)^36^.

## Supplementary Material

Supplemental Material
