# Supplementary material for: Synthesis and biological evaluation of 4-phenoxy-phenyl isoxazoles as novel acetyl-CoA carboxylase inhibitors
Source: J Enzyme Inhib Med Chem. 2021 Jun 8;36(1):1236–47. doi: 10.1080/14756366.2021.1936514 (PMC8205039; doi:10.1080/14756366.2021.1936514)
Supplement: Supplemental Material [file IENZ_A_1936514_SM0260.pdf]

Supporting Information

Synthesis and biological evaluation of 4-phenoxy-phenyl isoxazoles as novel acetyl-CoA carboxylase inhibitors

Xin Wu[a], Yongbo Yu[a], Tonghui Huang[a, *]

[a] *Jiangsu Key Laboratory of New Drug Research and Clinical Pharmacy, School of Pharmacy, Xuzhou Medical University, Xuzhou 221004, China.*

Table of Contents

1. Structures of HTVS candidates from the ChemDiv database (Fig. S1)...................S2

2. Single concentration inhibition rates of compounds on cancer cells (Fig. S2)……S3

3. ^{1}H, ^{13}C NMR, HRMS and HPLC spectra of target compounds…..........................S4

4. An abbreviation list……………………………………………………………S38

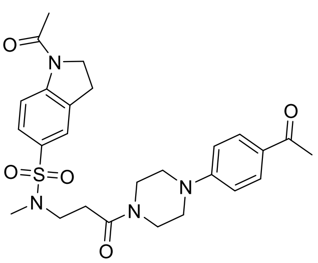

C450-0875

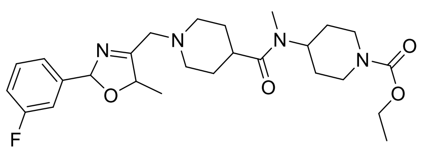

C522-2022

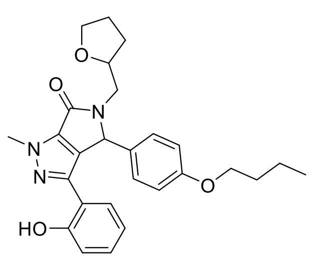

D074-0032

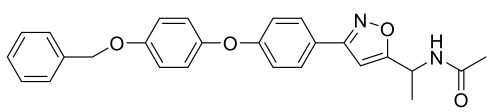

D098-1432 (6a)

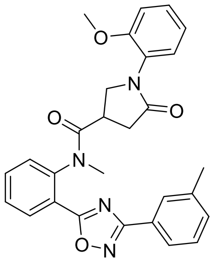

D400-1402

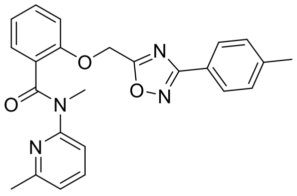

D400-3199

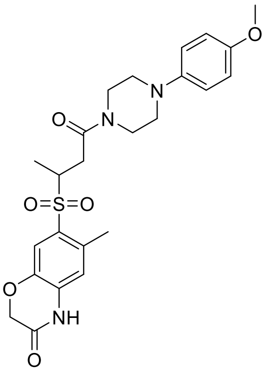

E667-1377

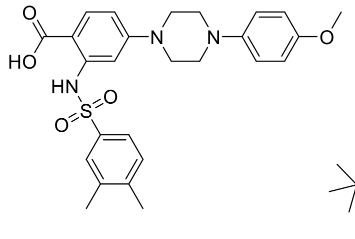

F292-0452

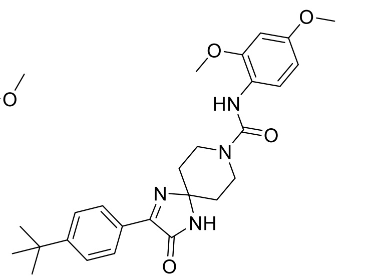

F697-0546

Fig. S1 Structures of HTVS candidates from the ChemDiv database.

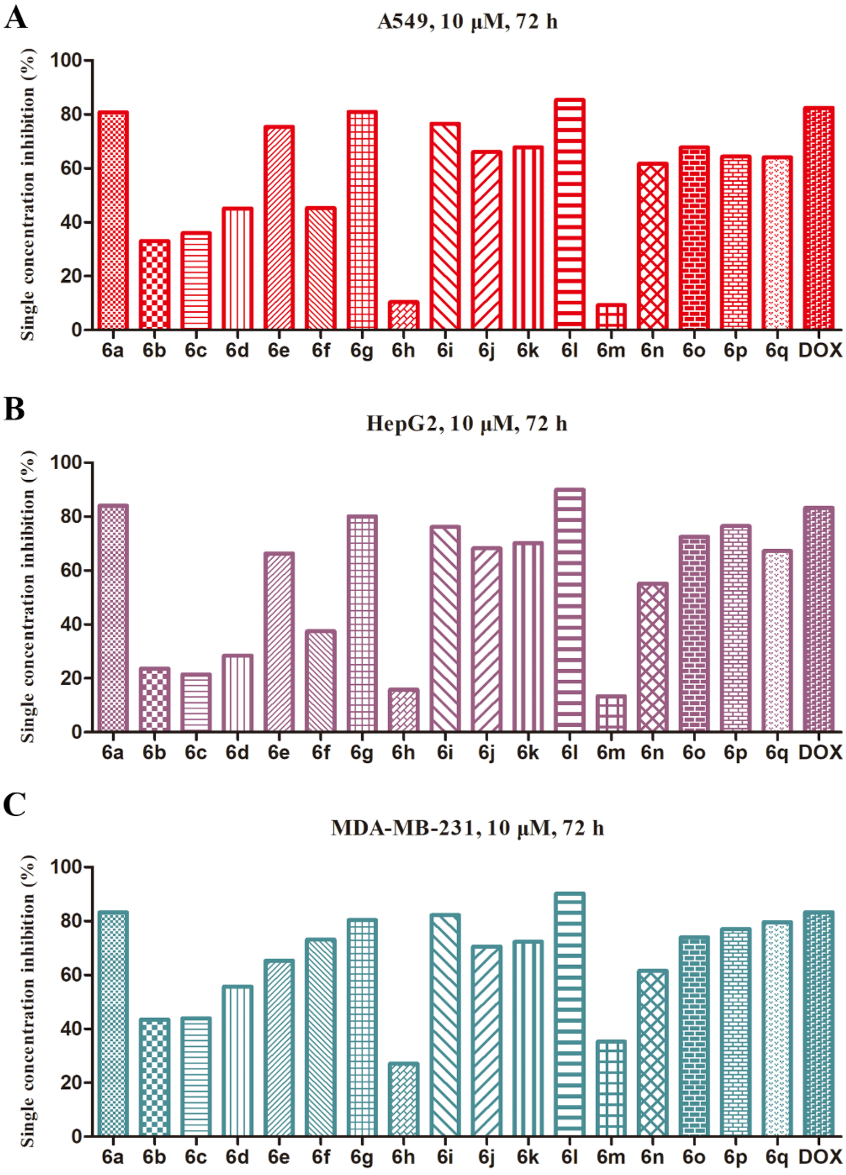

Fig. S2 Single concentration inhibition rates of target compounds on A549 (A), HepG2 (B), and MDA-MB-231 (C) cells.

*N-(1-(3-(4-(4-(benzyloxy)phenoxy)phenyl)isoxazol-5-yl)ethyl)acetamide (**6a**).*

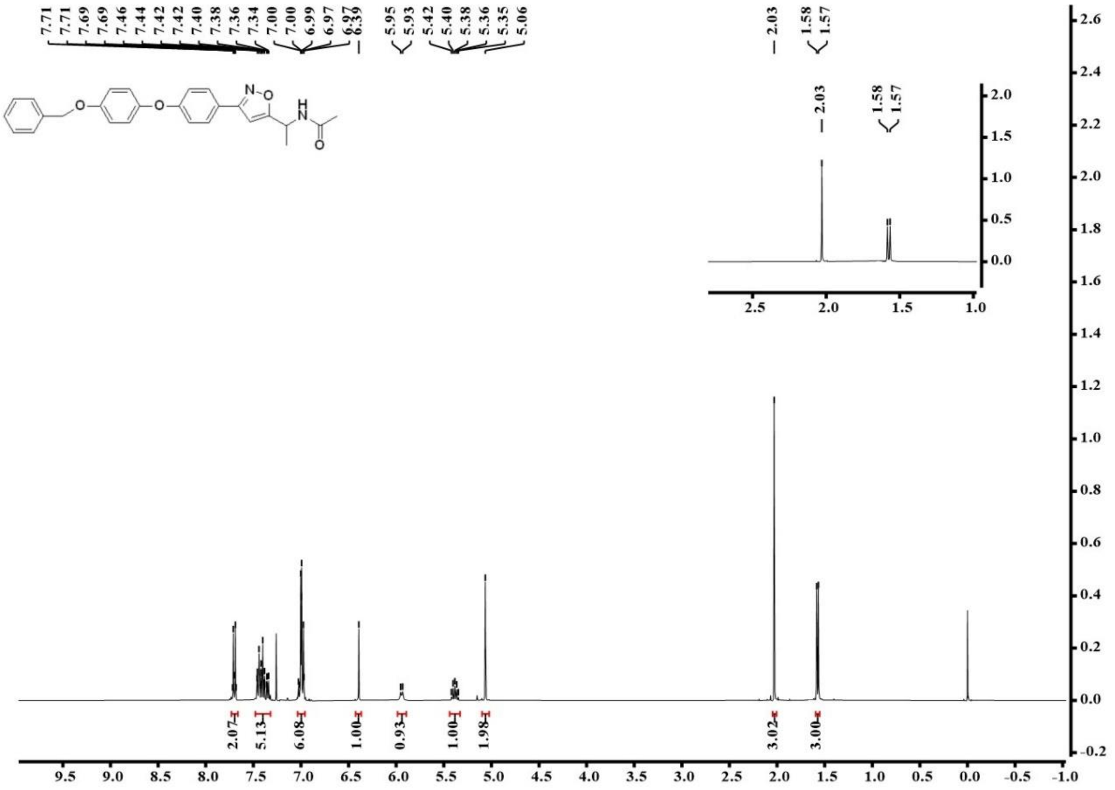

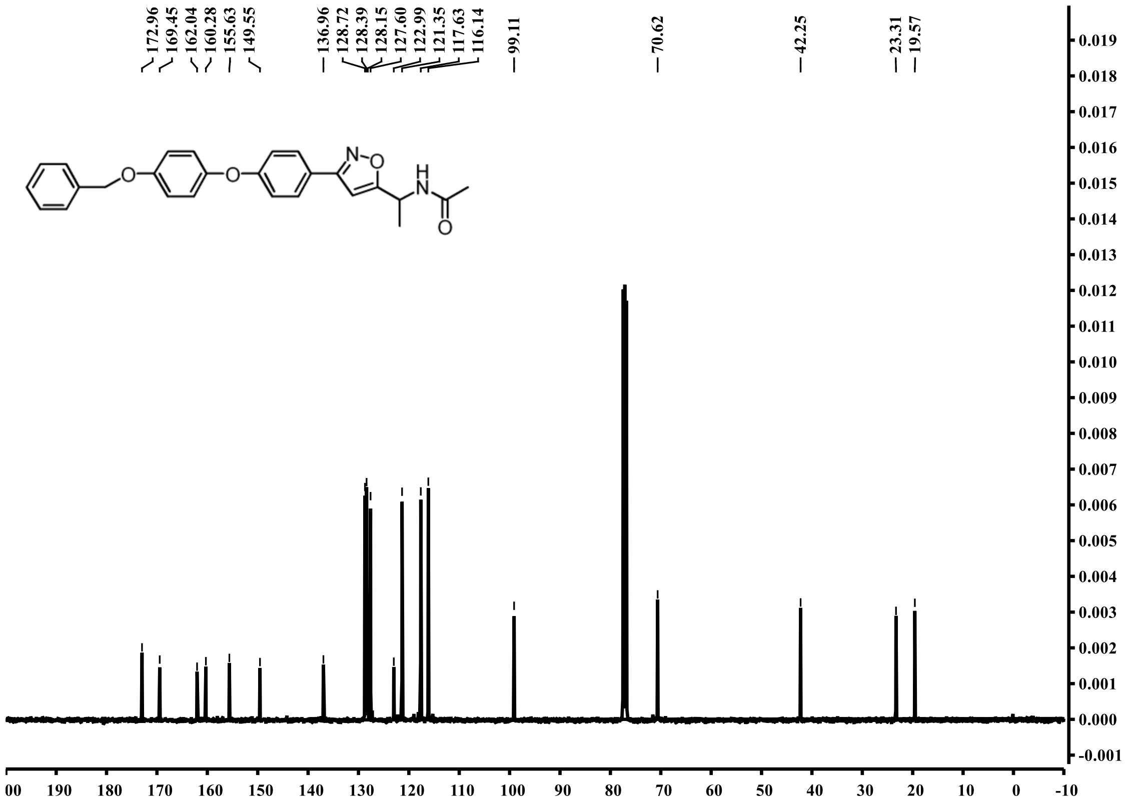

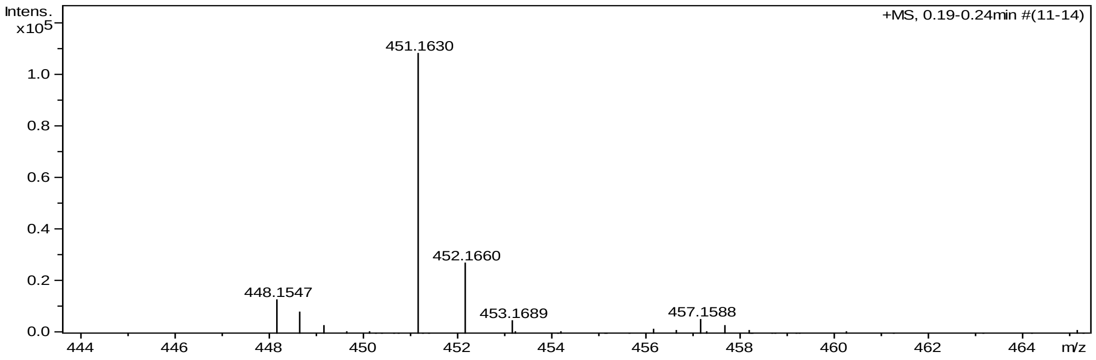

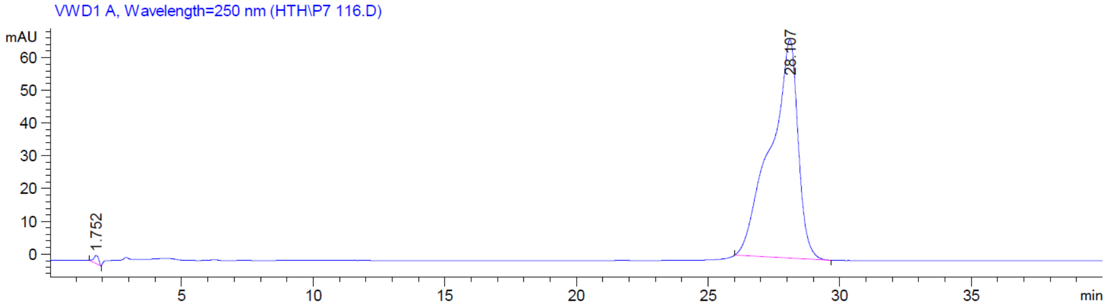

峰 #	保留时间 [min]	类型	峰宽 [min]	峰面积 [mAU*s]	峰高 [mAU]	峰面积 %
1	1.752	BB	0.2071	32.26326	2.49058	0.6579
2	28.107	BB	0.9996	4871.59180	66.73970	99.3421

*N-(1-(3-(4-(4-hydroxyphenoxy)phenyl)isoxazol-5-yl)ethyl)acetamide (**6b**).*

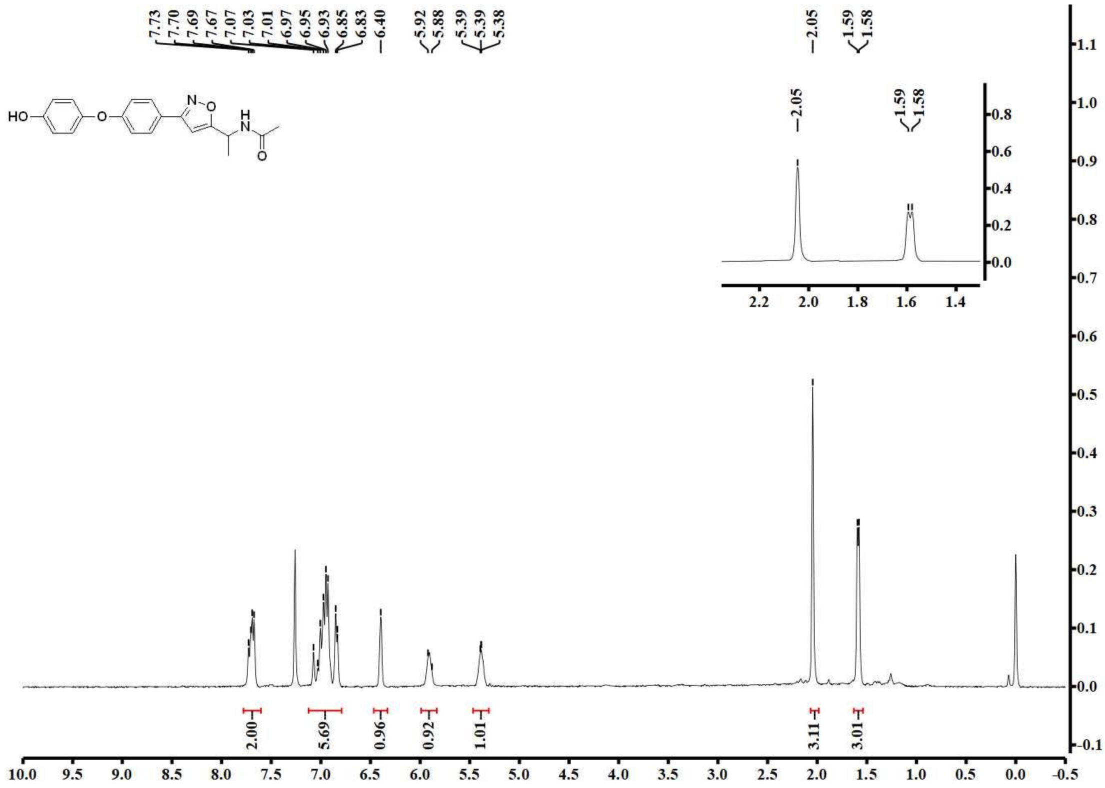

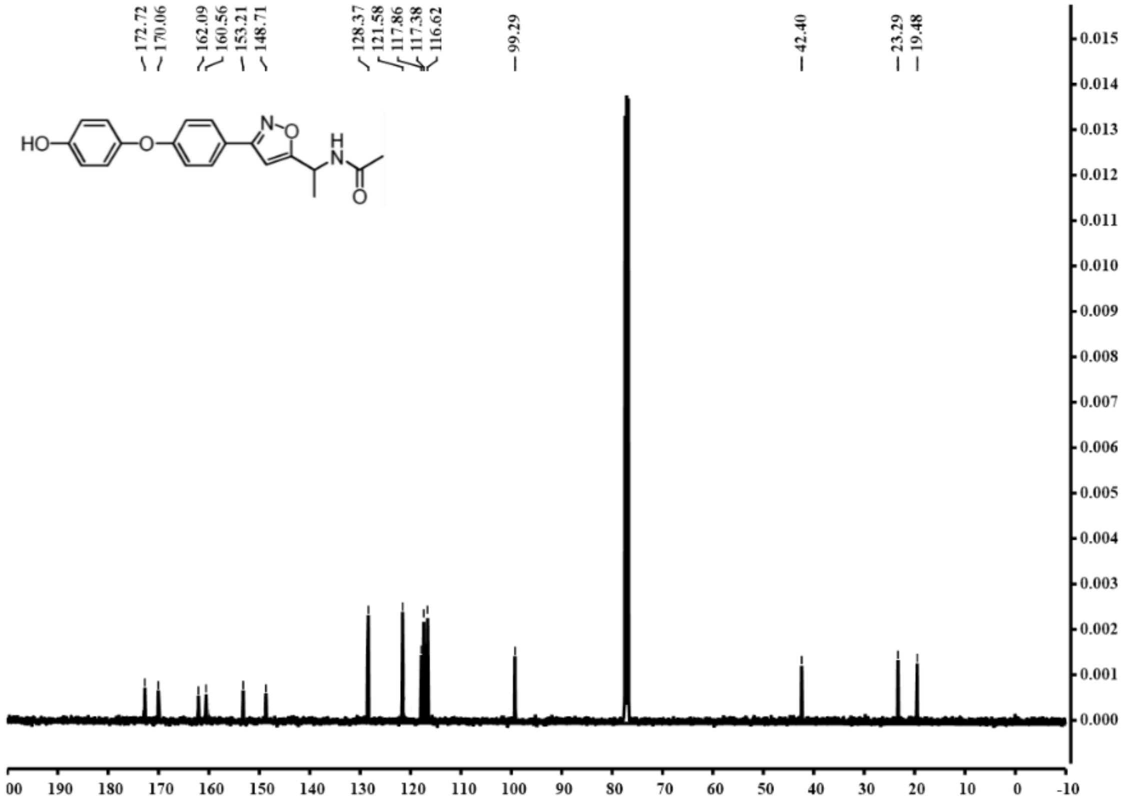

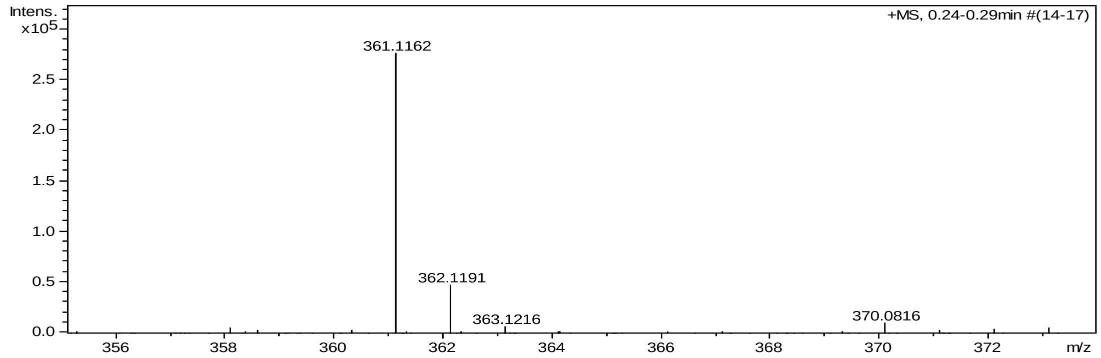

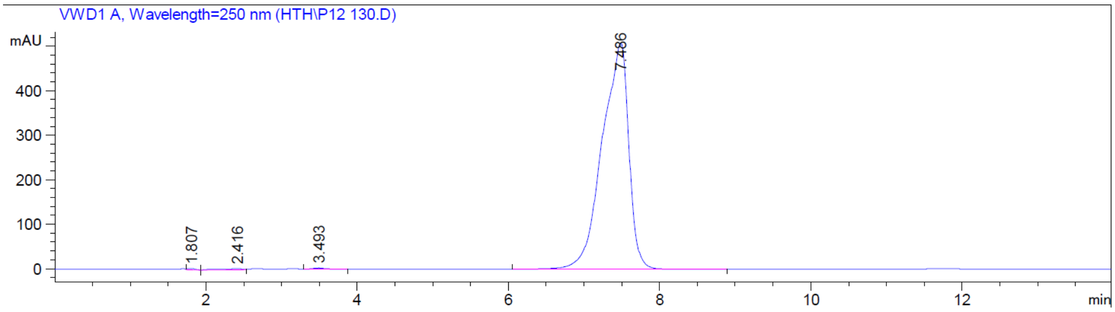

峰 #	保留时间 [min]	类型	峰宽 [min]	峰面积 [mAU*s]	峰高 [mAU]	峰面积 %
1	1.807	VB	0.1139	17.67358	2.40138	0.1416
2	2.416	BV	0.3198	47.49784	1.88633	0.3805
3	3.493	VB	0.1776	32.84626	2.74585	0.2631
4	7.486	BB	0.3328	1.23848e4	507.37000	99.2148

N-(1-(3-(4-(4-methoxyphenoxy)phenyl)isoxazol-5-yl)ethyl)acetamide (***6c***).

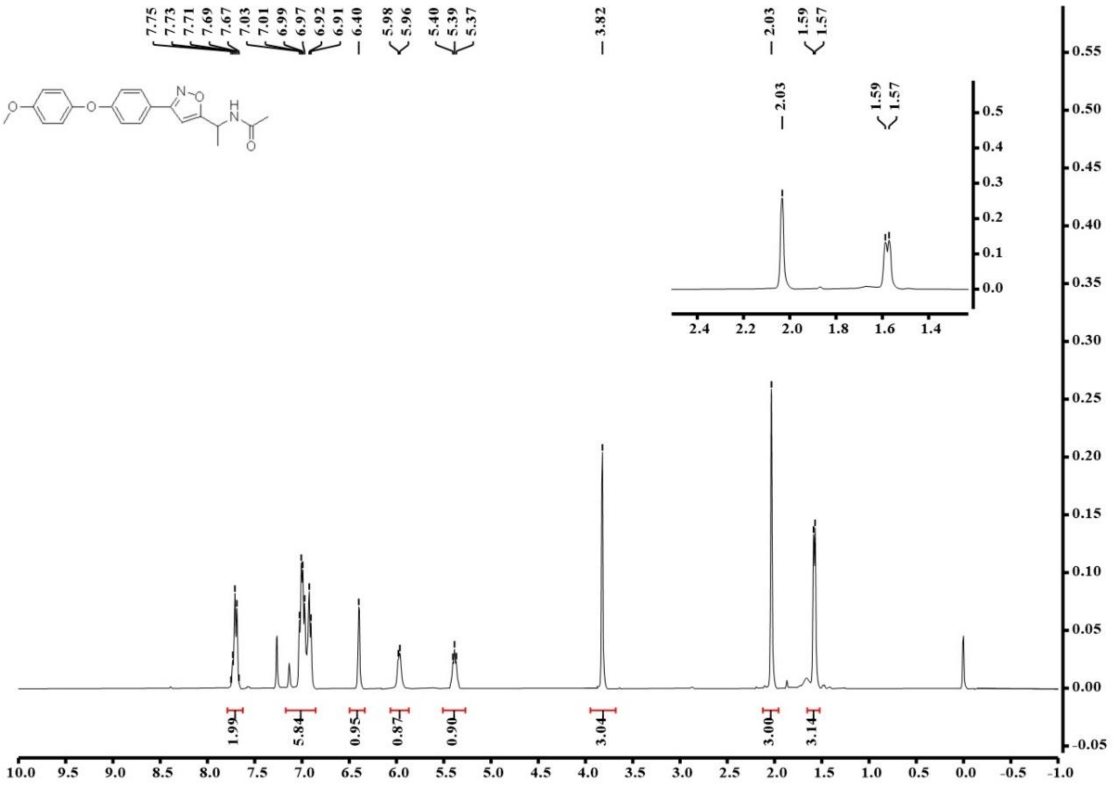

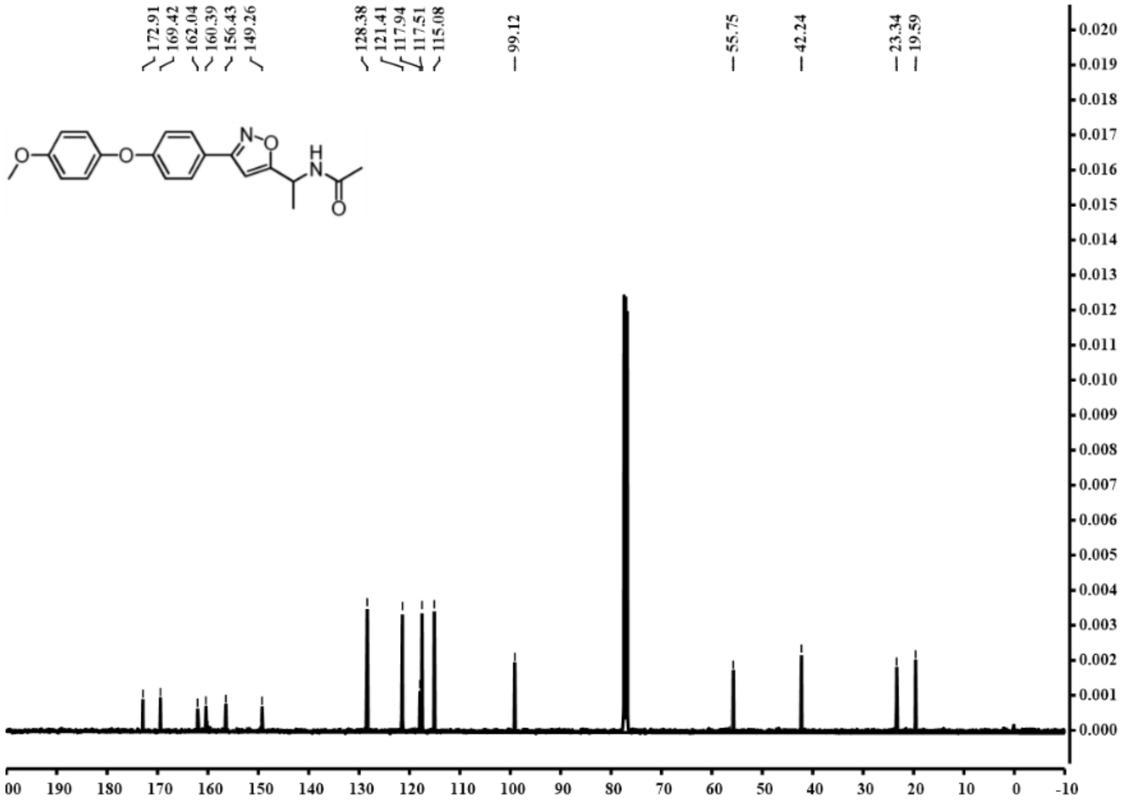

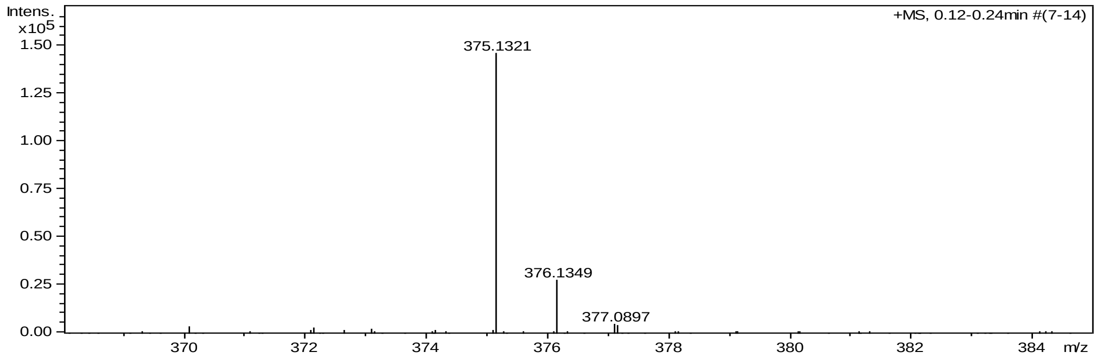

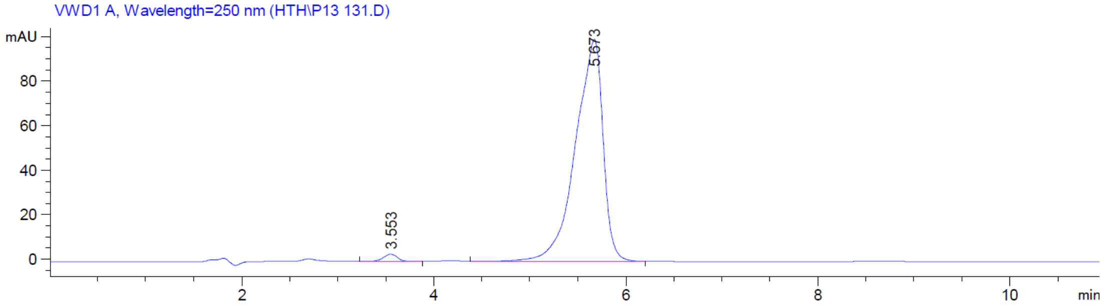

峰 #	保留时间 [min]	类型	峰宽 [min]	峰面积 [mAU*s]	峰高 [mAU]	峰面积 %
1	3.553	BB	0.1580	32.95910	3.25814	1.6293
2	5.673	BB	0.2746	1989.91992	99.80005	98.3707

N-(1-(3-(4-(4-ethoxyphenoxy)phenyl)isoxazol-5-yl)ethyl)acetamide (***6d***).

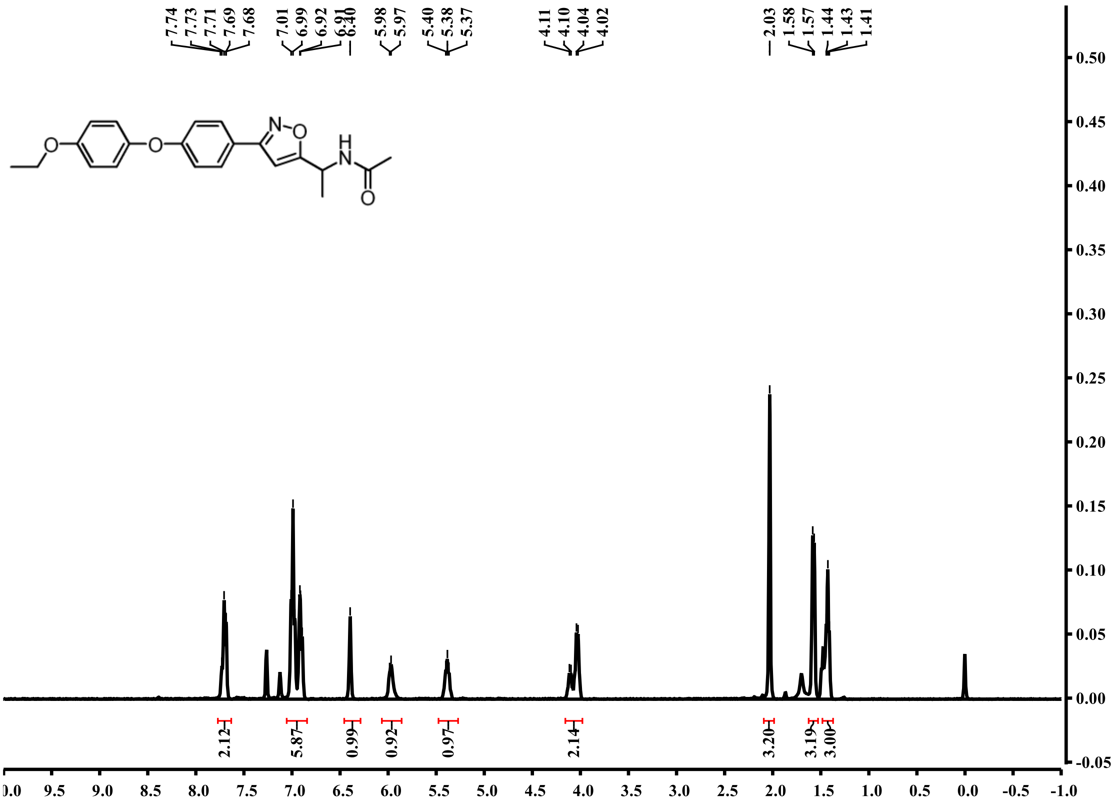

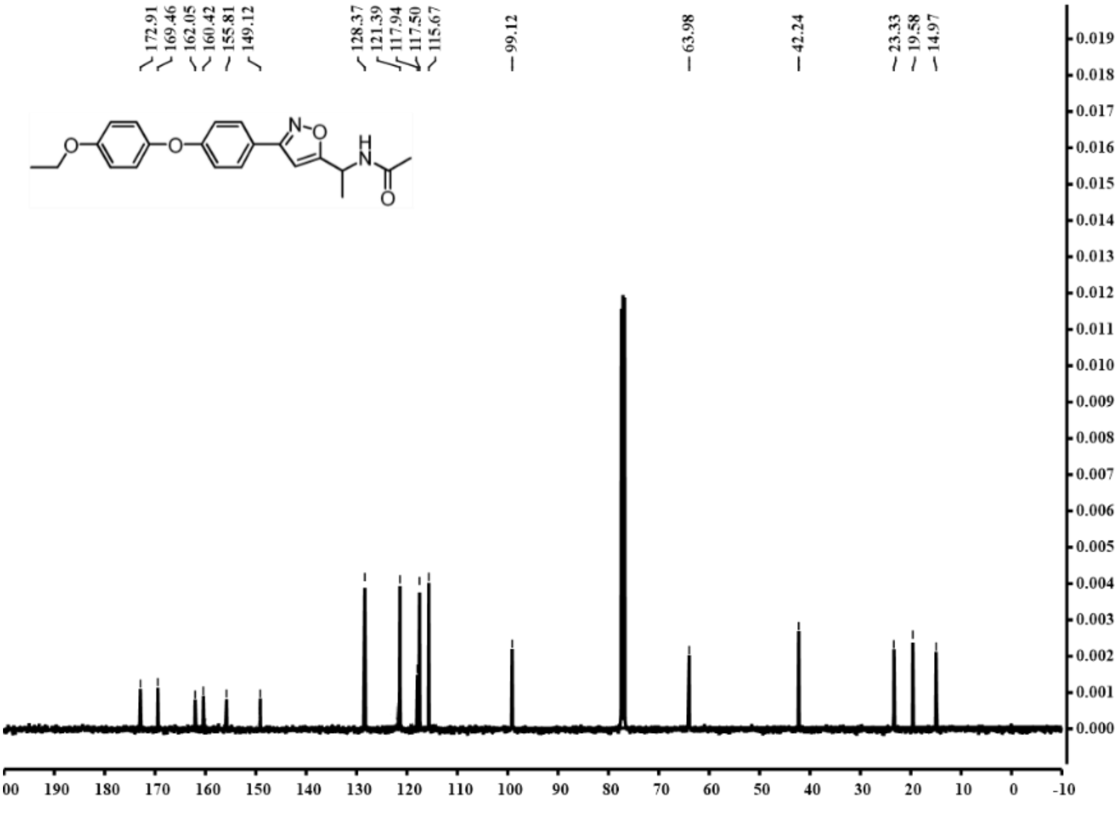

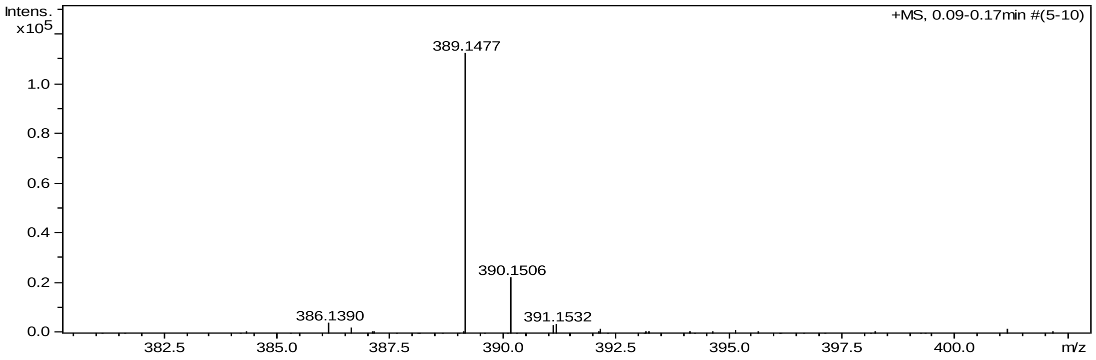

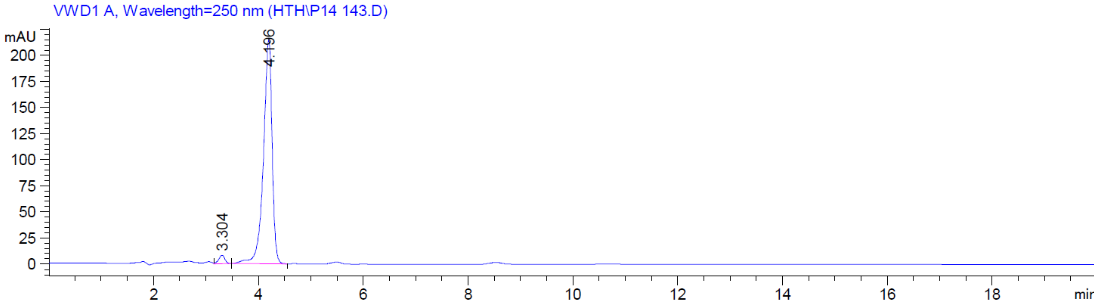

峰 #	保留时间 [min]	类型	峰宽 [min]	峰面积 [mAU*s]	峰高 [mAU]	峰面积 %
1	3.304	VB	0.1242	65.62139	8.12634	2.5883
2	4.196	BB	0.1720	2469.68799	215.08691	97.4117

*N-(1-(3-(4-(4-propoxyphenoxy)phenyl)isoxazol-5-yl)ethyl)acetamide (**6e**)*.

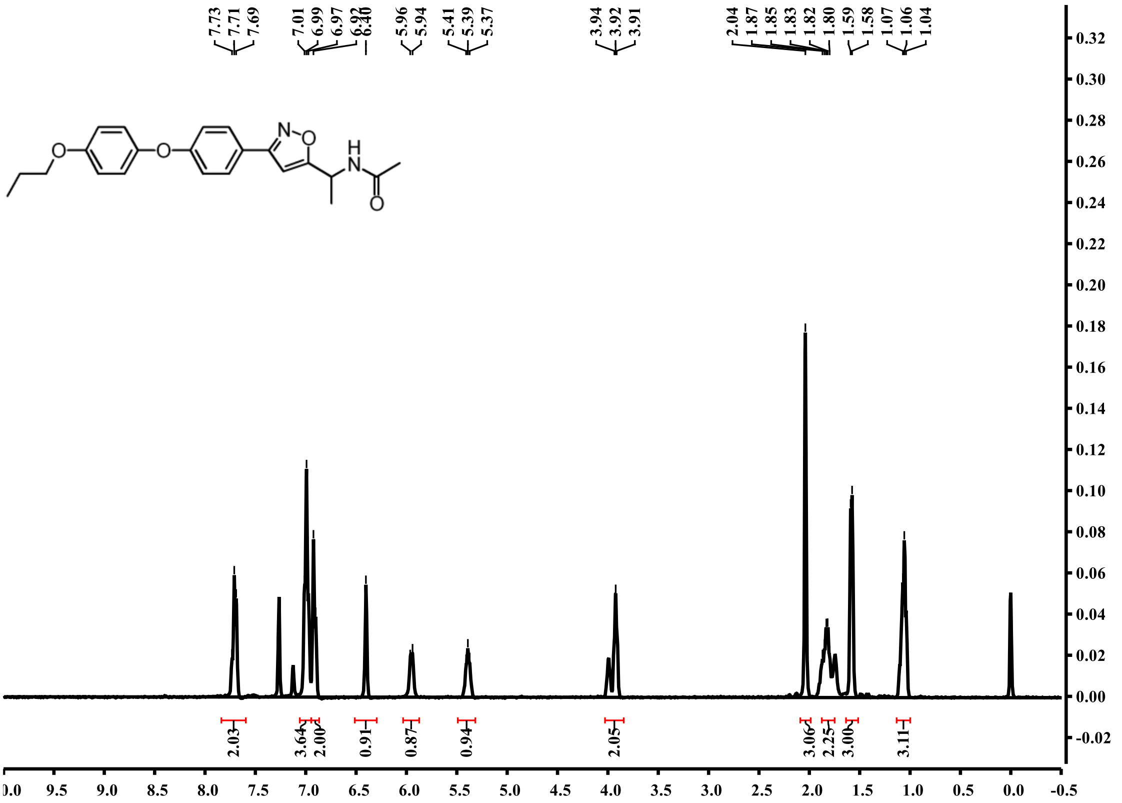

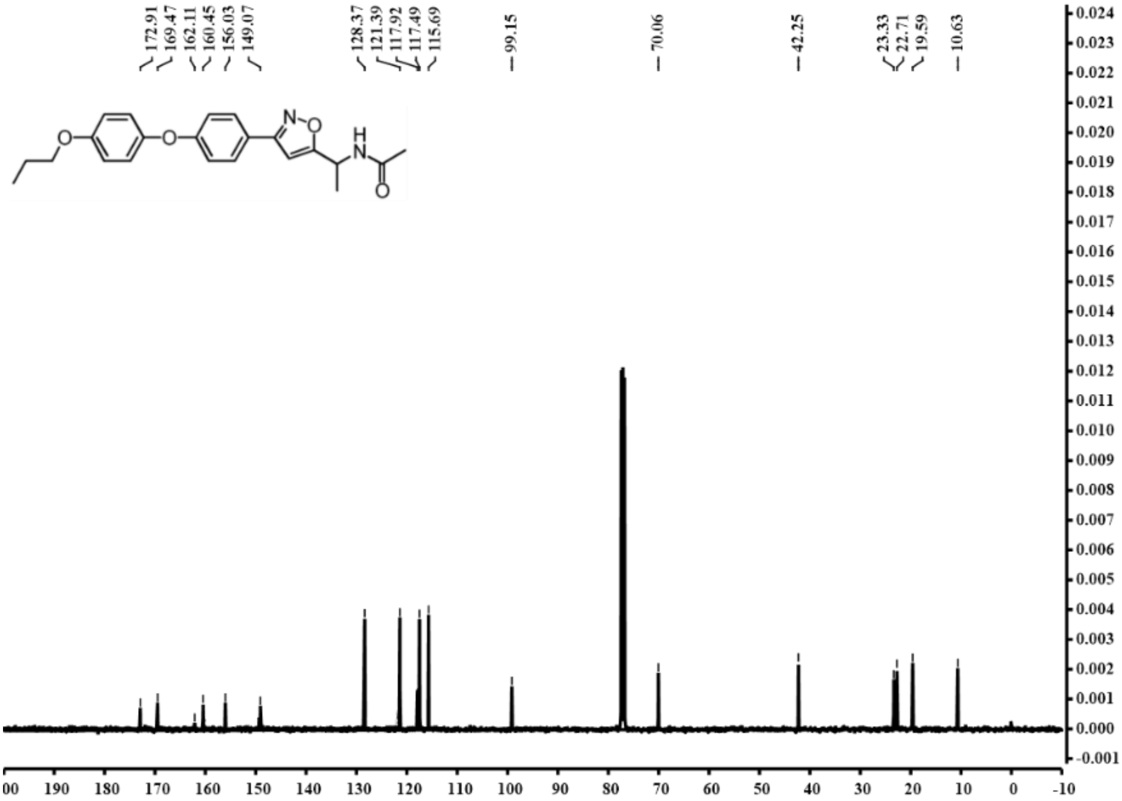

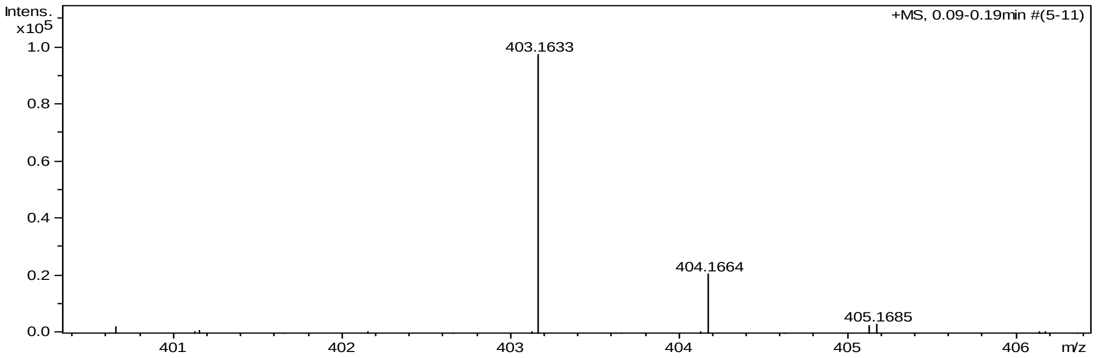

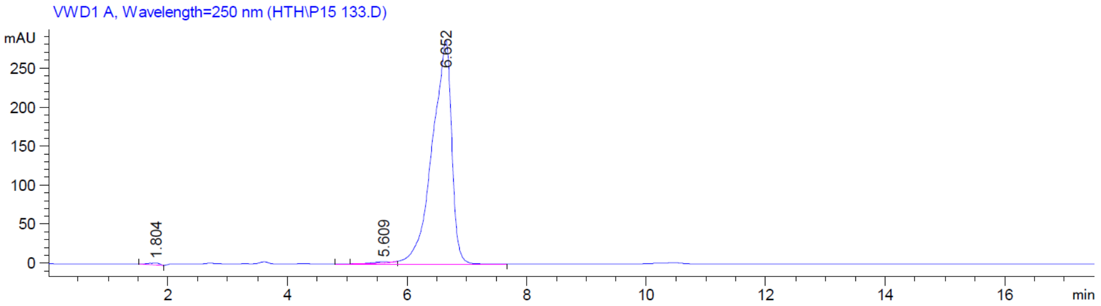

峰 #	保留时间 [min]	类型	峰宽 [min]	峰面积 [mAU*s]	峰高 [mAU]	峰面积 %
1	1.804	BB	0.1647	30.00153	2.59944	0.4573
2	5.609	BV E	0.2519	31.89456	1.72320	0.4861
3	6.652	VB R	0.3091	6498.81494	286.02844	99.0566

*N-(1-(3-(4-(4-isopropoxyphenoxy)phenyl)isoxazol-5-yl)ethyl)acetamide (**6f**).*

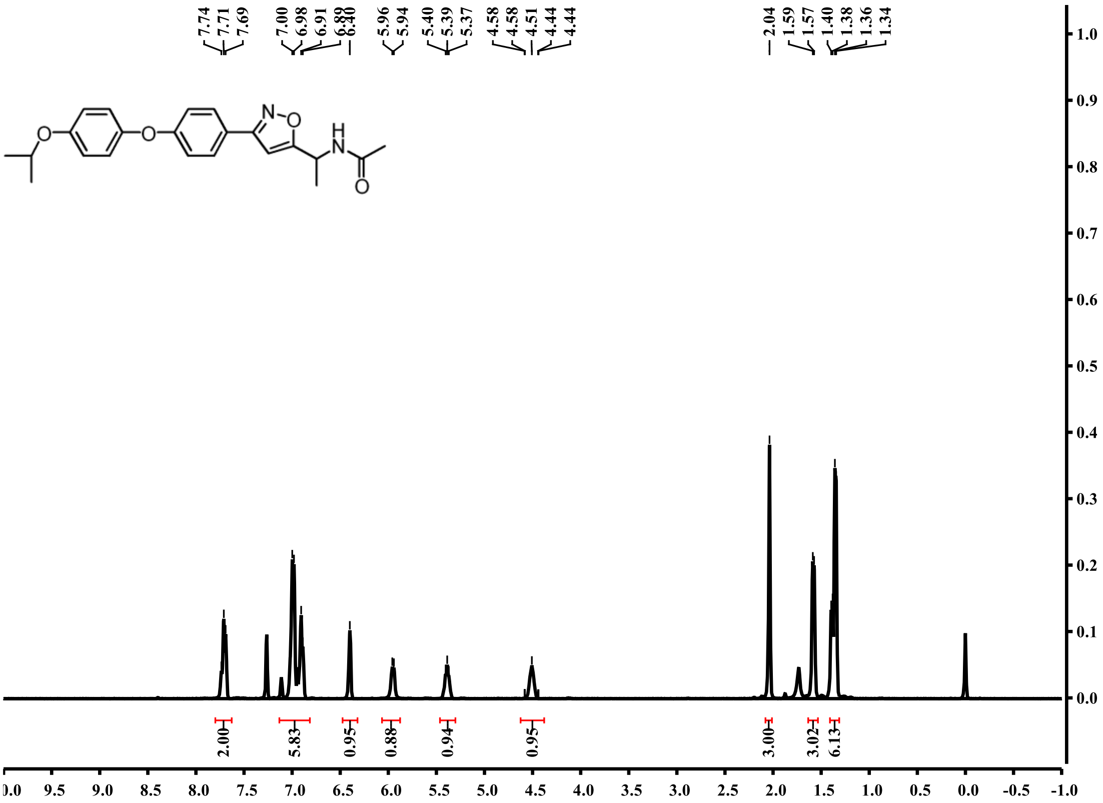

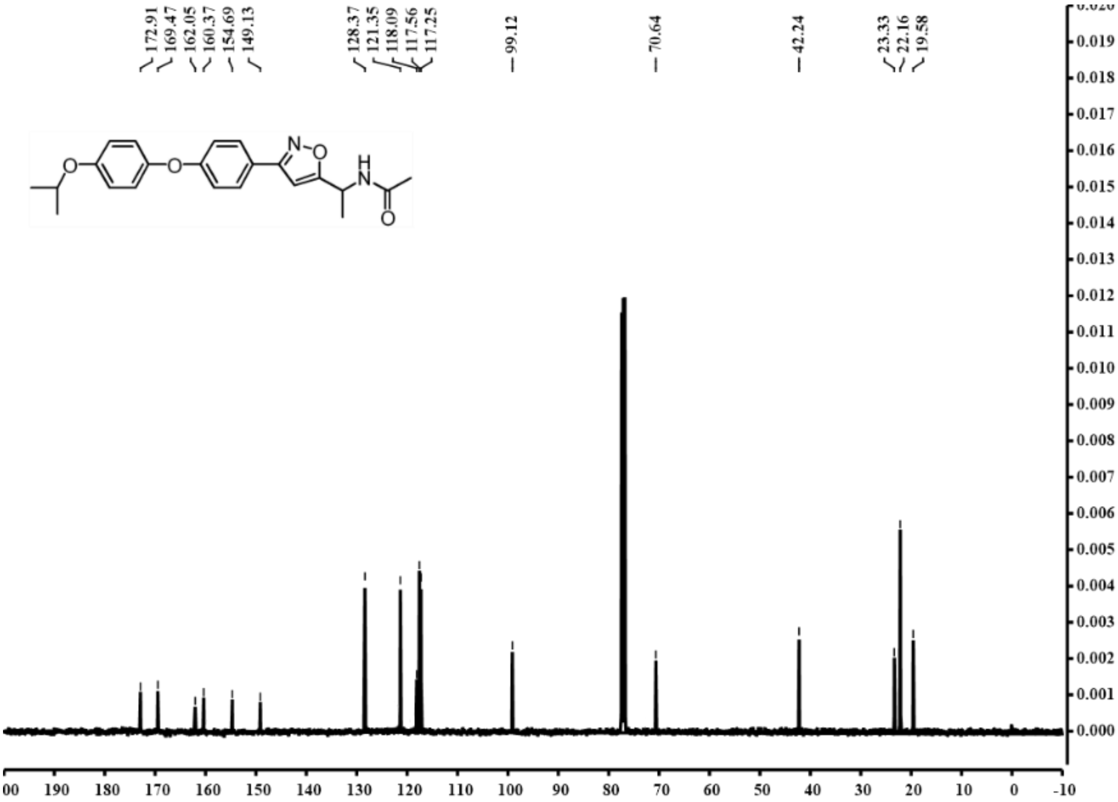

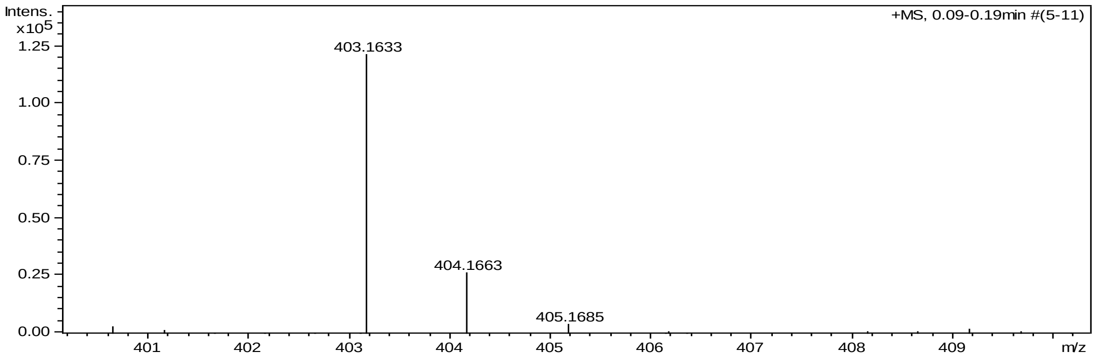

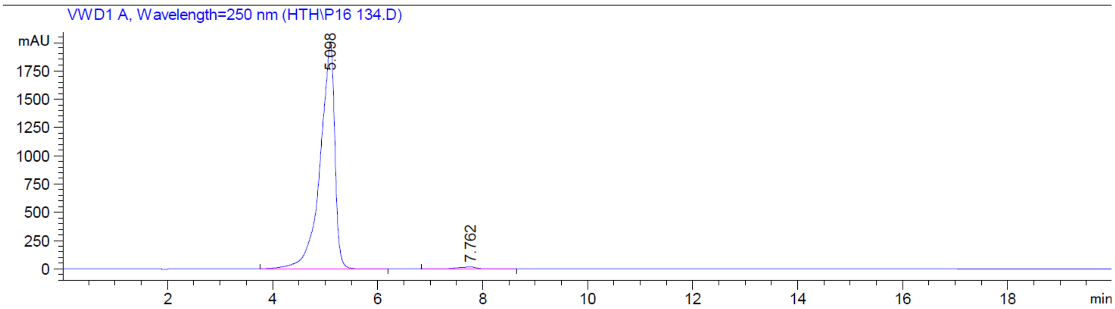

峰 #	保留时间 [min]	类型	峰宽 [min]	峰面积 [mAU*s]	峰高 [mAU]	峰面积 %
1	5.098	BB	0.2667	3.87223e4	1992.87671	98.8164
2	7.762	BB	0.3525	463.79077	18.12761	1.1836

*N-(1-(3-(4-(4-(cyclopropylmethoxy)phenoxy)phenyl)isoxazol-5-yl)ethyl)acetamide (**6g**).*

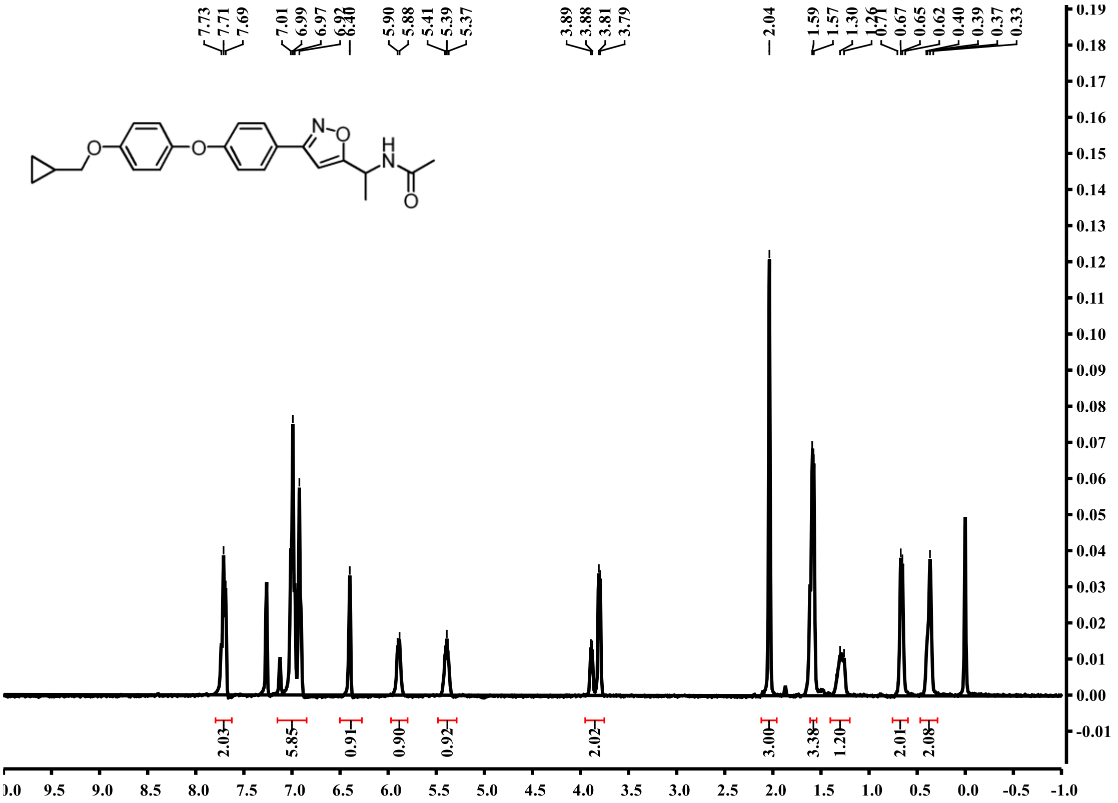

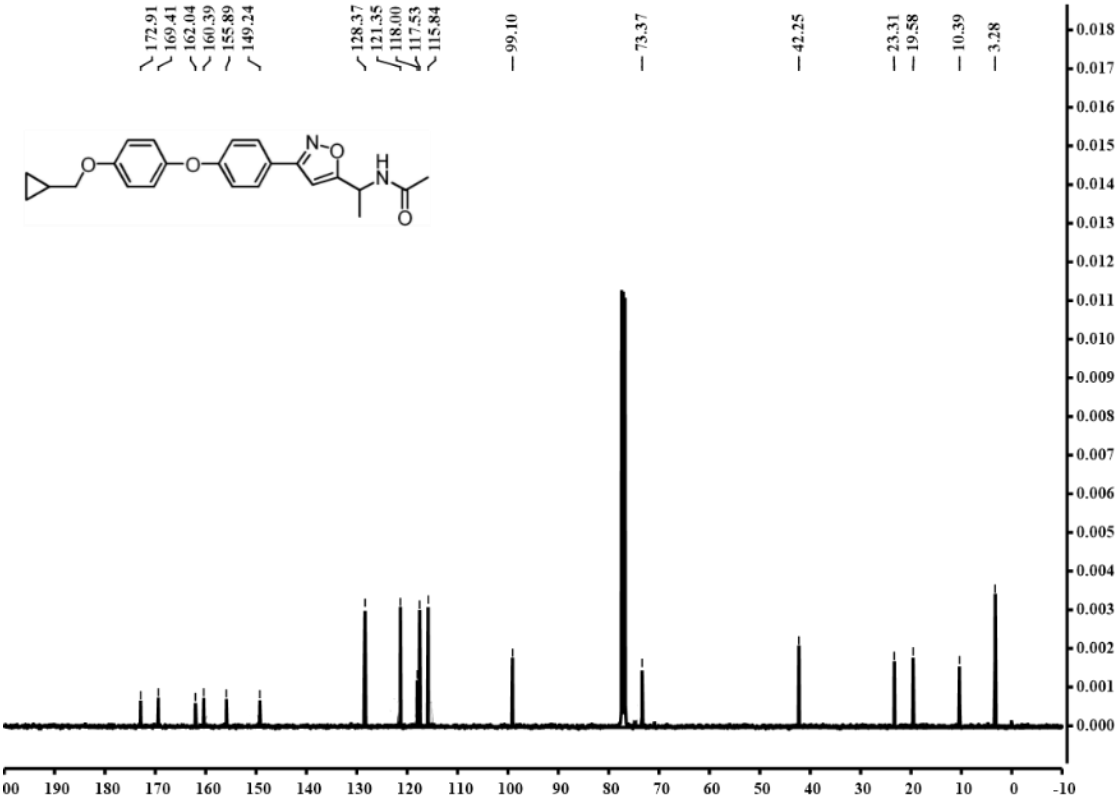

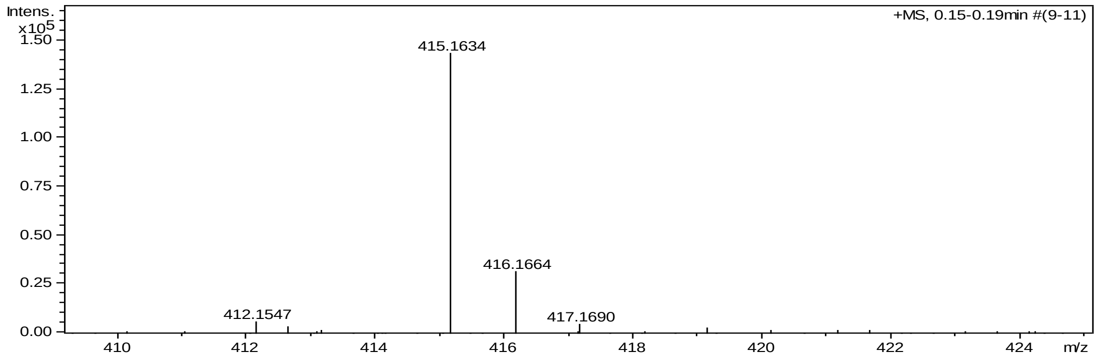

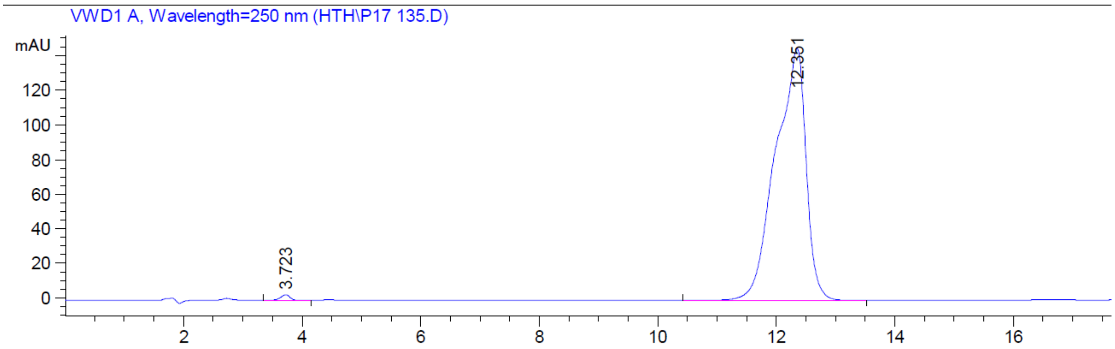

峰 #	保留时间 [min]	类型	峰宽 [min]	峰面积 [mAU*s]	峰高 [mAU]	峰面积 %
1	3.723	BB	0.1689	34.92889	3.21100	0.6679
2	12.351	BB	0.4846	5194.64453	145.47261	99.3321

*N-(1-(3-(4-phenoxyphenyl)isoxazol-5-yl)ethyl)acetamide (**6h**).*

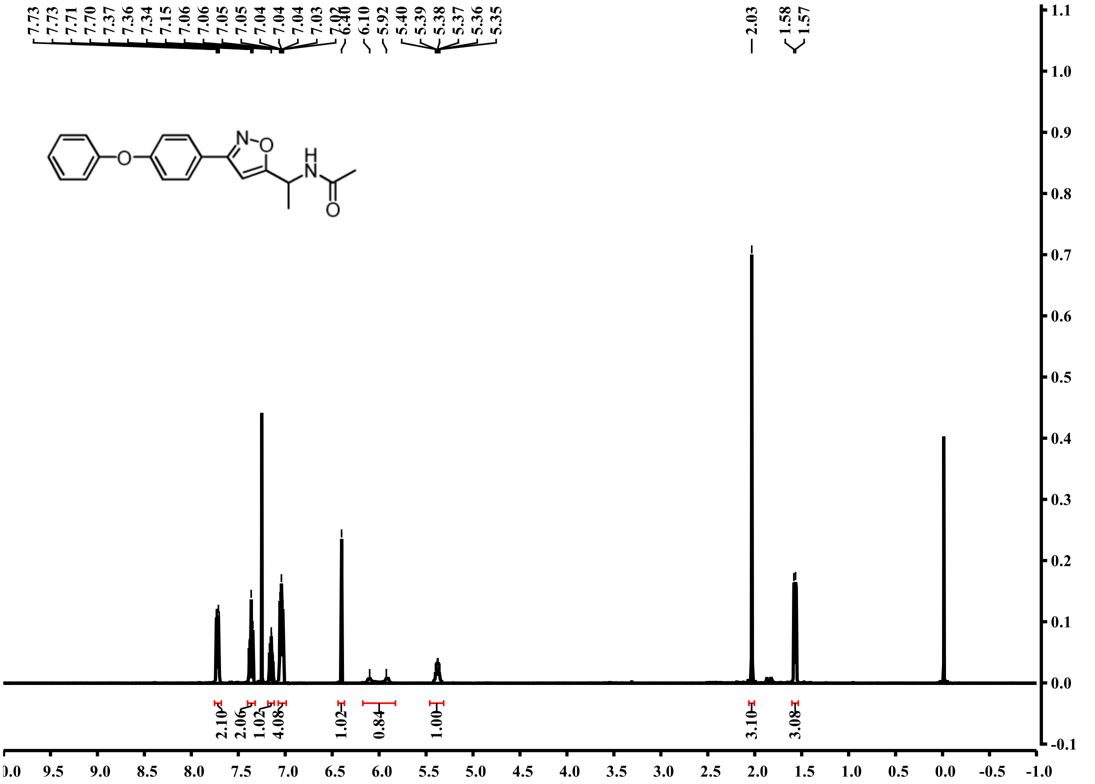

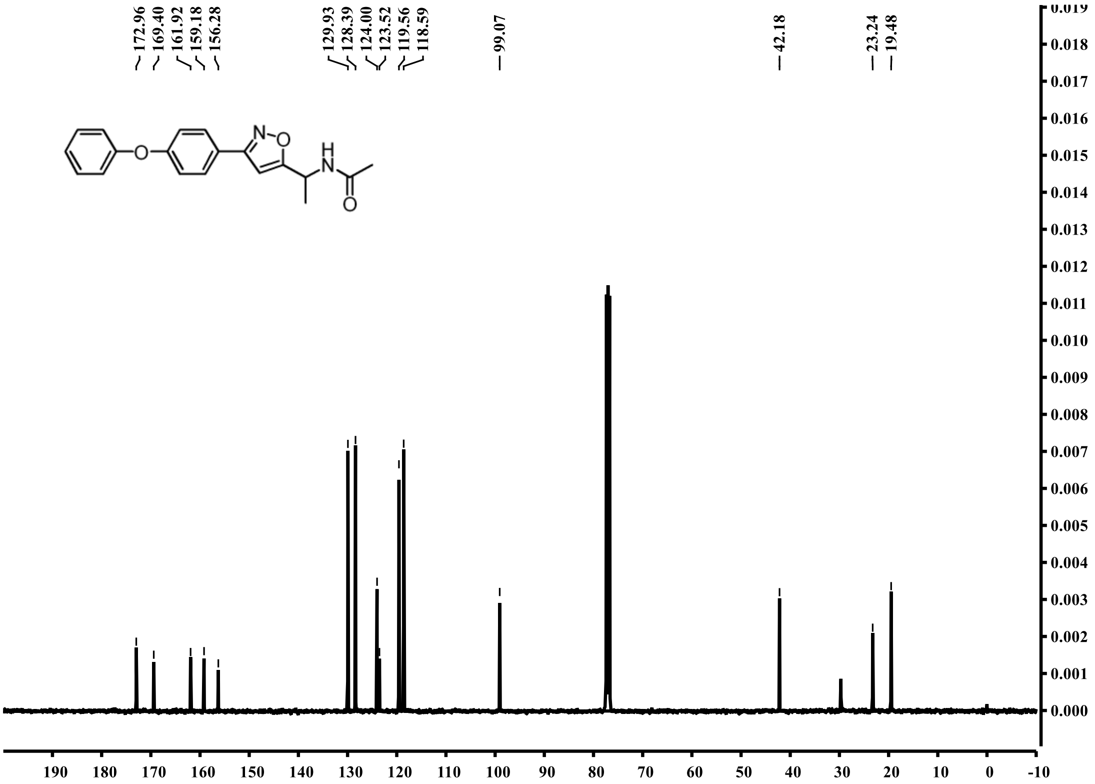

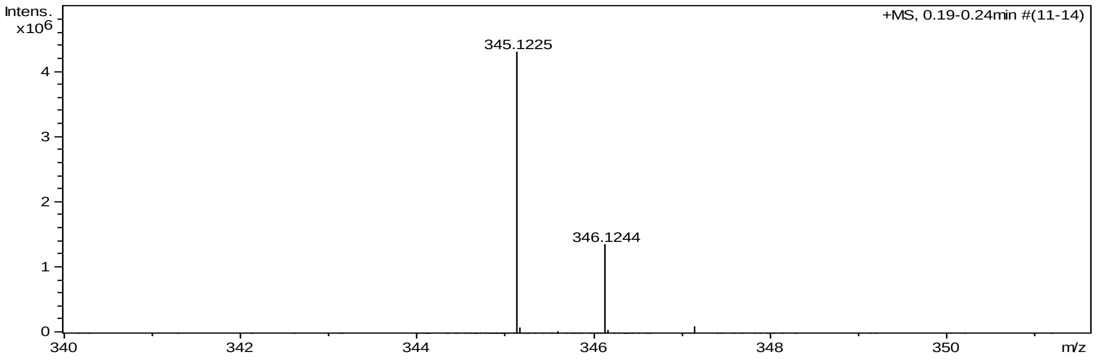

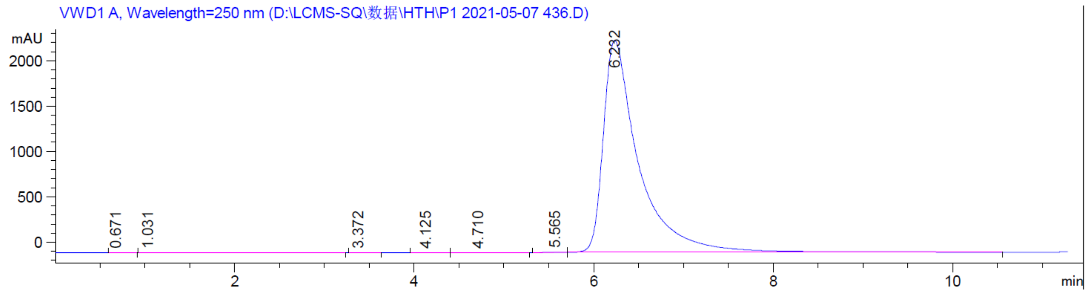

峰 #	保留时间 [min]	类型	峰宽 [min]	峰面积	峰高	峰面积 %
1	0.082	BB	0.0334	5.12129e5	2.55757e5	3.0672
2	6.310	BB	0.5126	1.61849e7	4.25482e5	96.9328

*N-(1-(3-(4-phenoxyphenyl)isoxazol-5-yl)ethyl)propionamide (**6i**).*

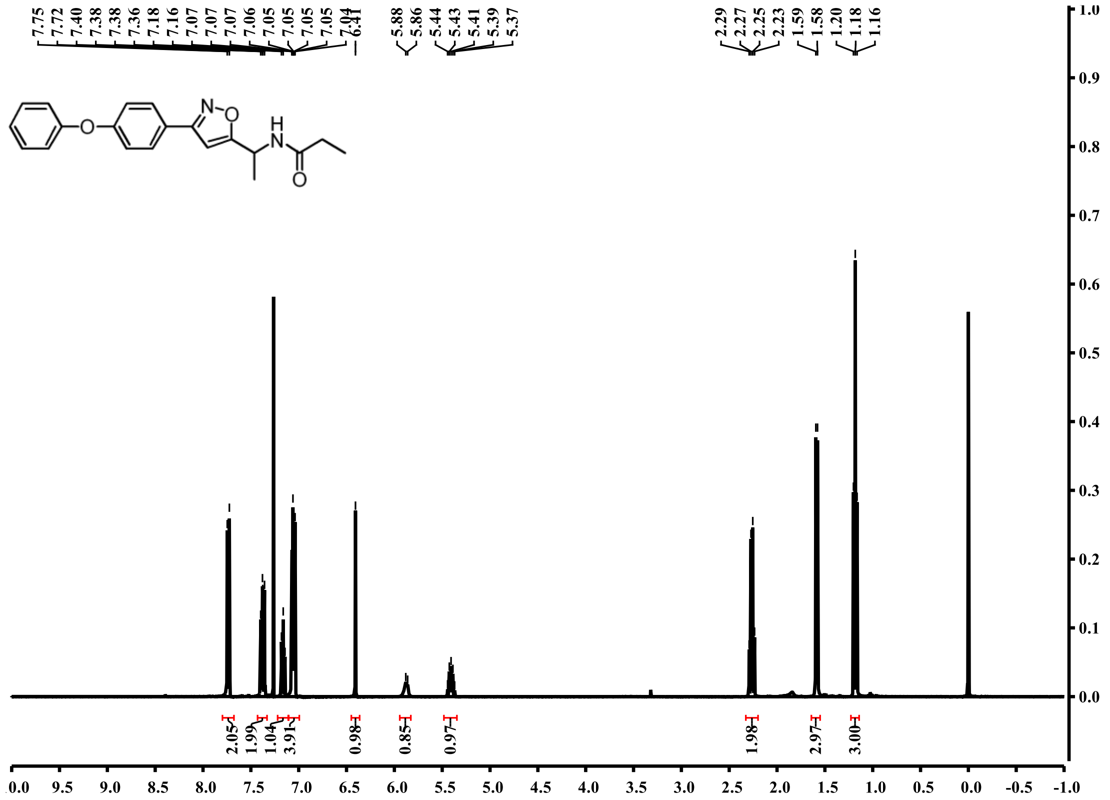

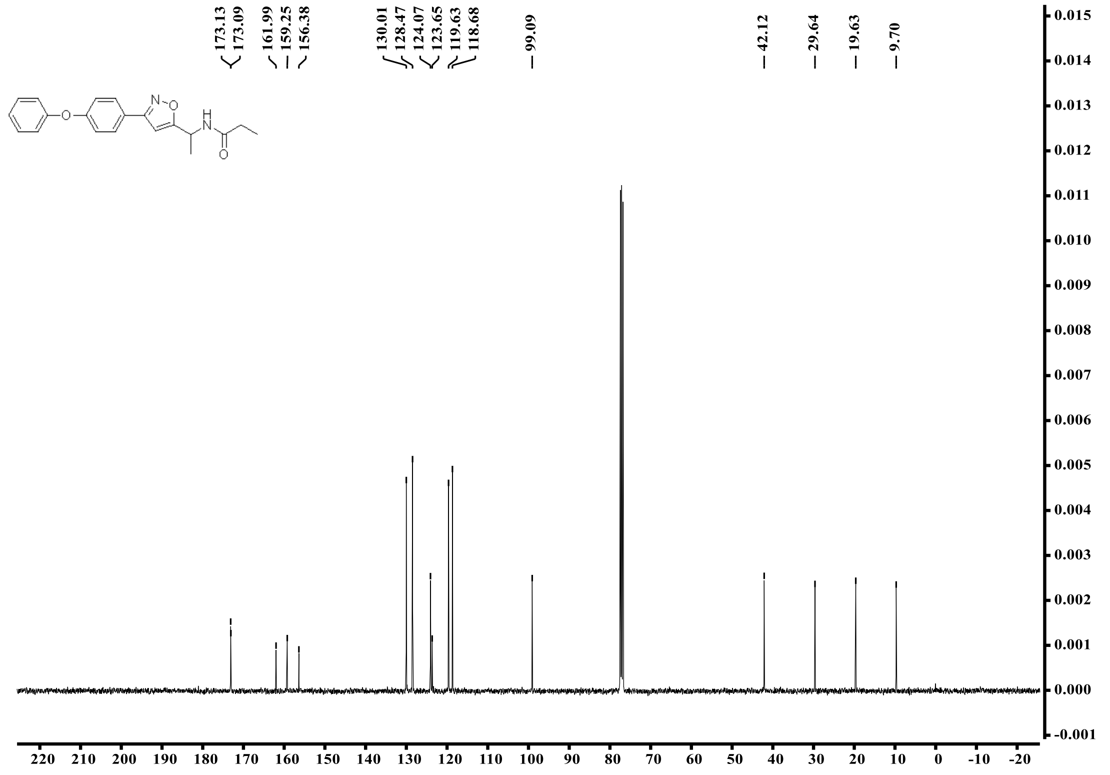

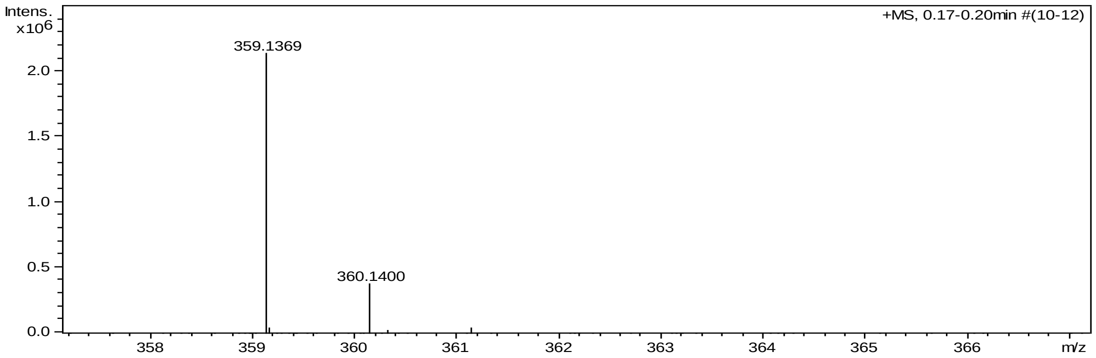

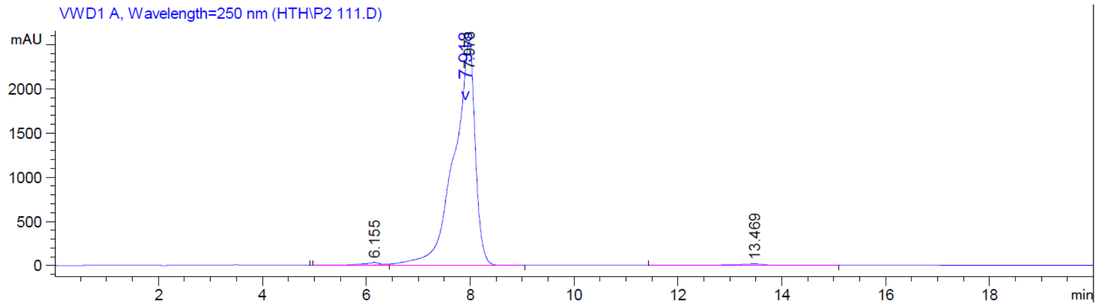

峰 #	保留时间 [min]	类型	峰宽 [min]	峰面积 [mAU*s]	峰高 [mAU]	峰面积 %
1	6.155	BV E	0.3159	643.24023	27.59850	0.8765
2	7.978	VB R	0.3865	7.20810e4	2529.31372	98.2241
3	13.469	BB	0.5389	659.95514	16.70578	0.8993

*N-(1-(3-(4-phenoxyphenyl)isoxazol-5-yl)ethyl)cyclopropanecarboxamide (**6j**).*

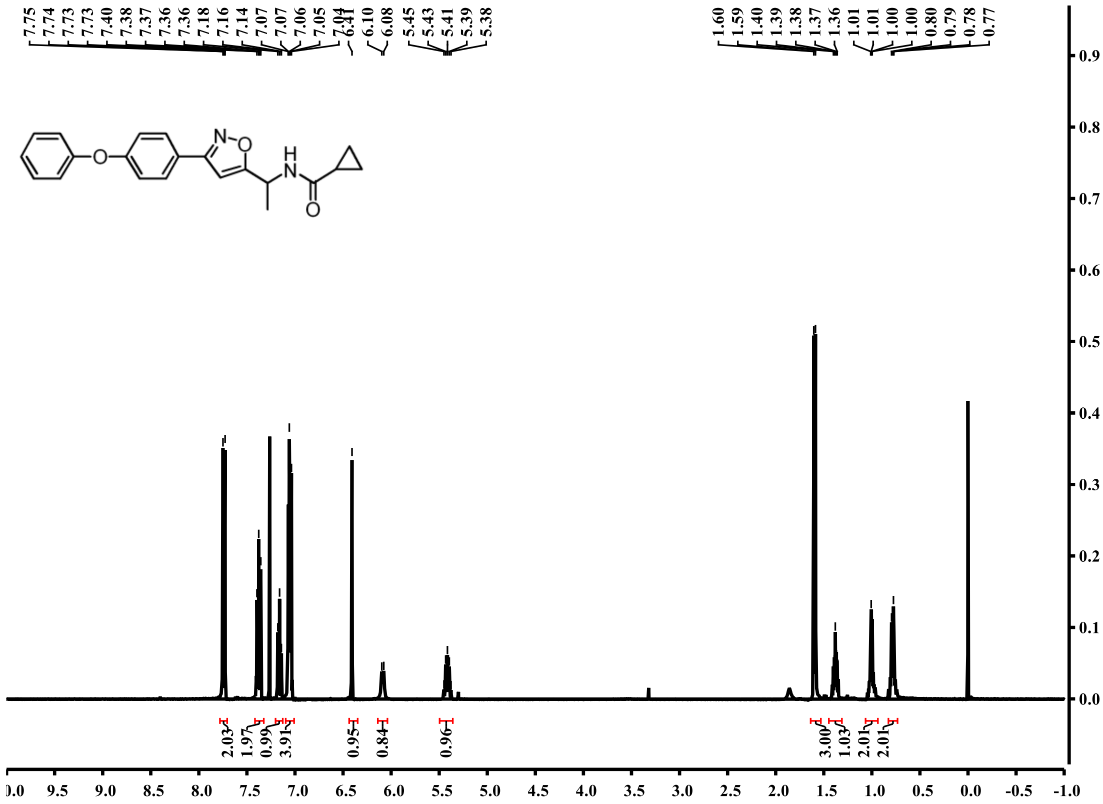

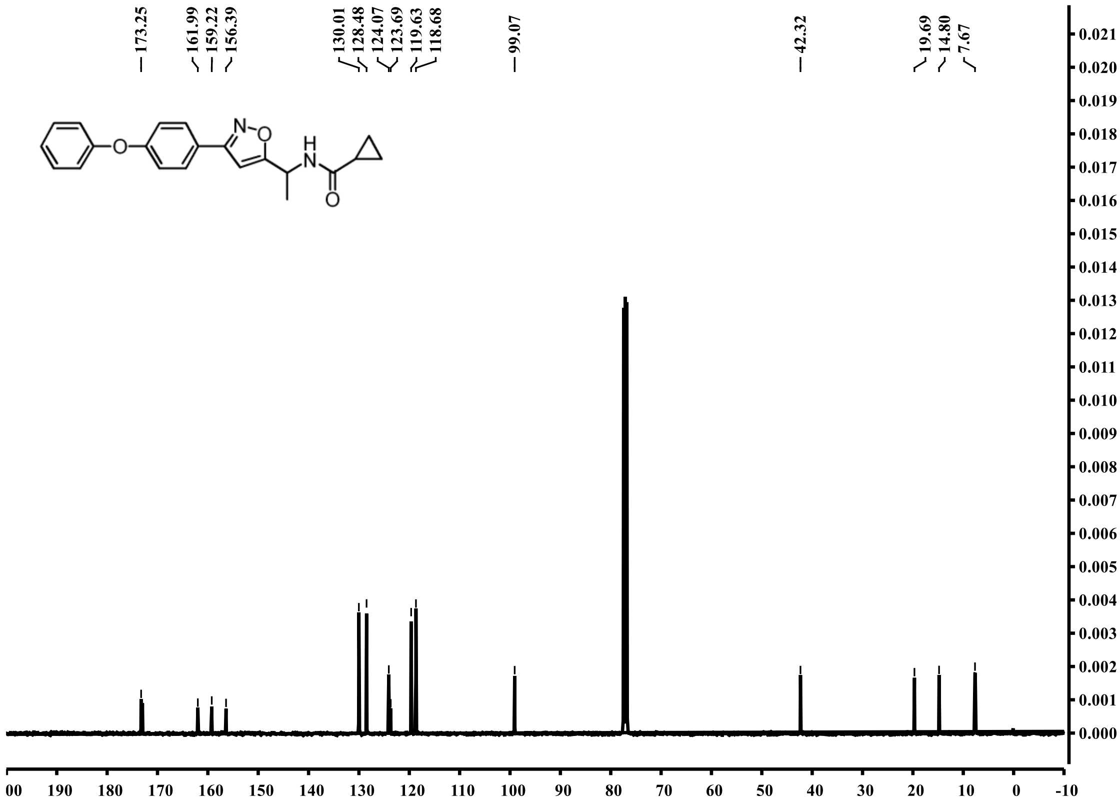

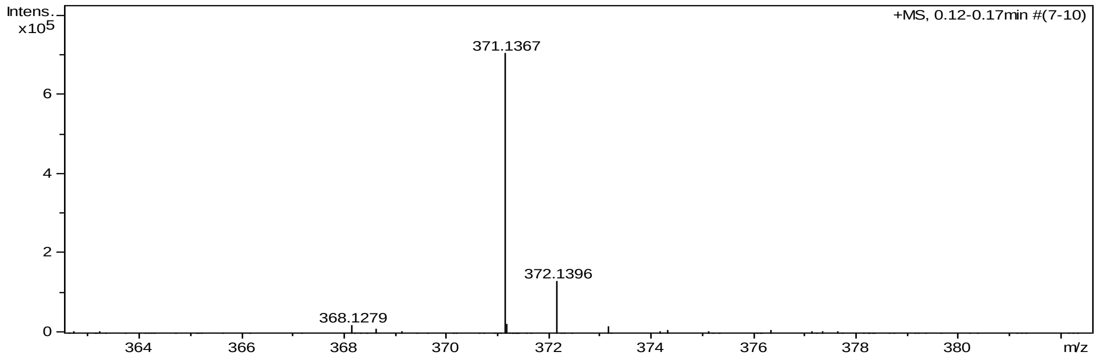

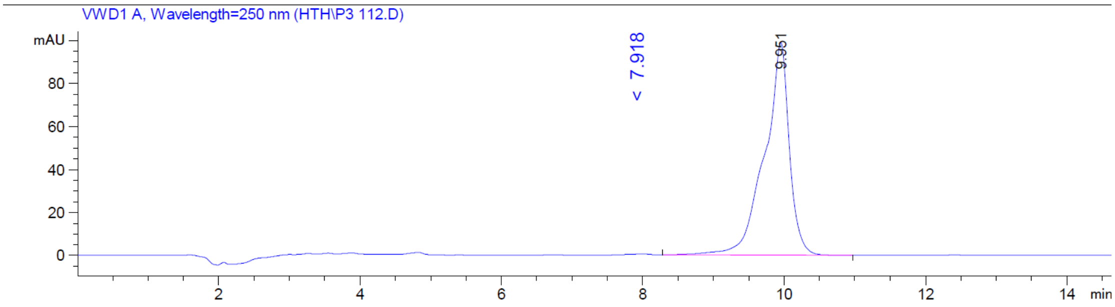

峰 #	保留时间 [min]	类型	峰宽 [min]	峰面积 [mAU*s]	峰高 [mAU]	峰面积 %
1	9.951	BB	0.3337	2404.38208	98.86014	100.0000

*N-(1-(3-(4-phenoxyphenyl)isoxazol-5-yl)ethyl)benzamide (**6k**).*

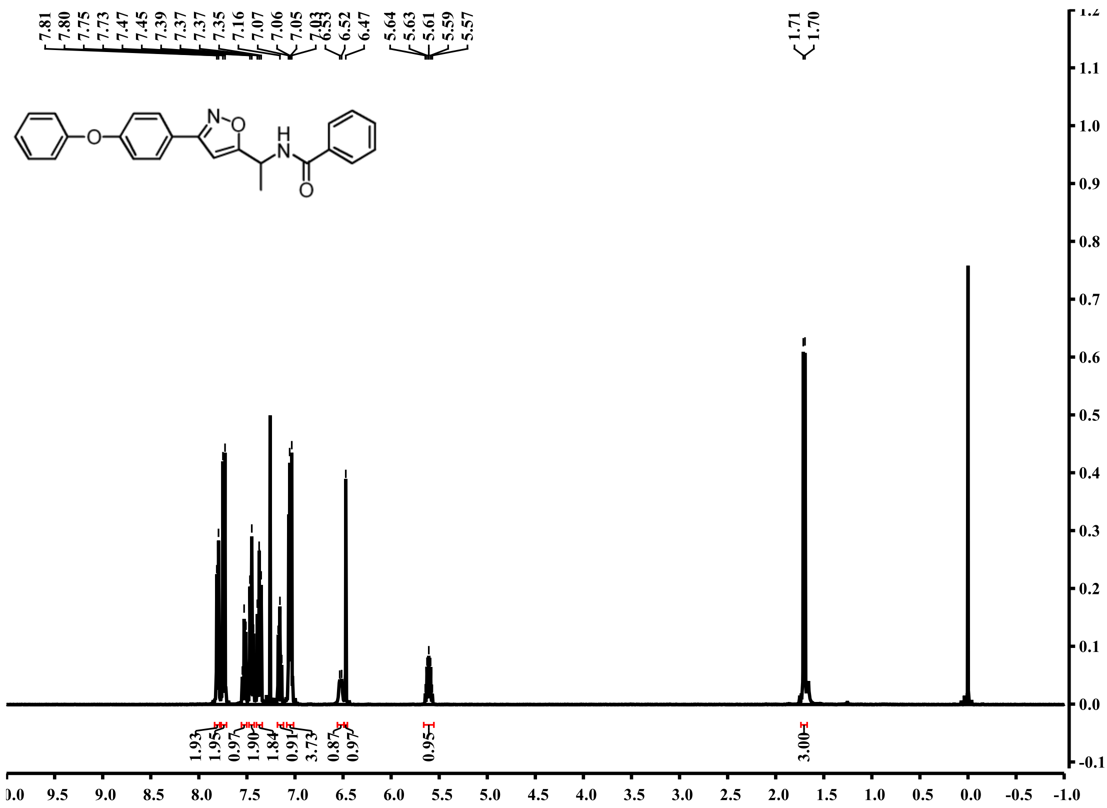

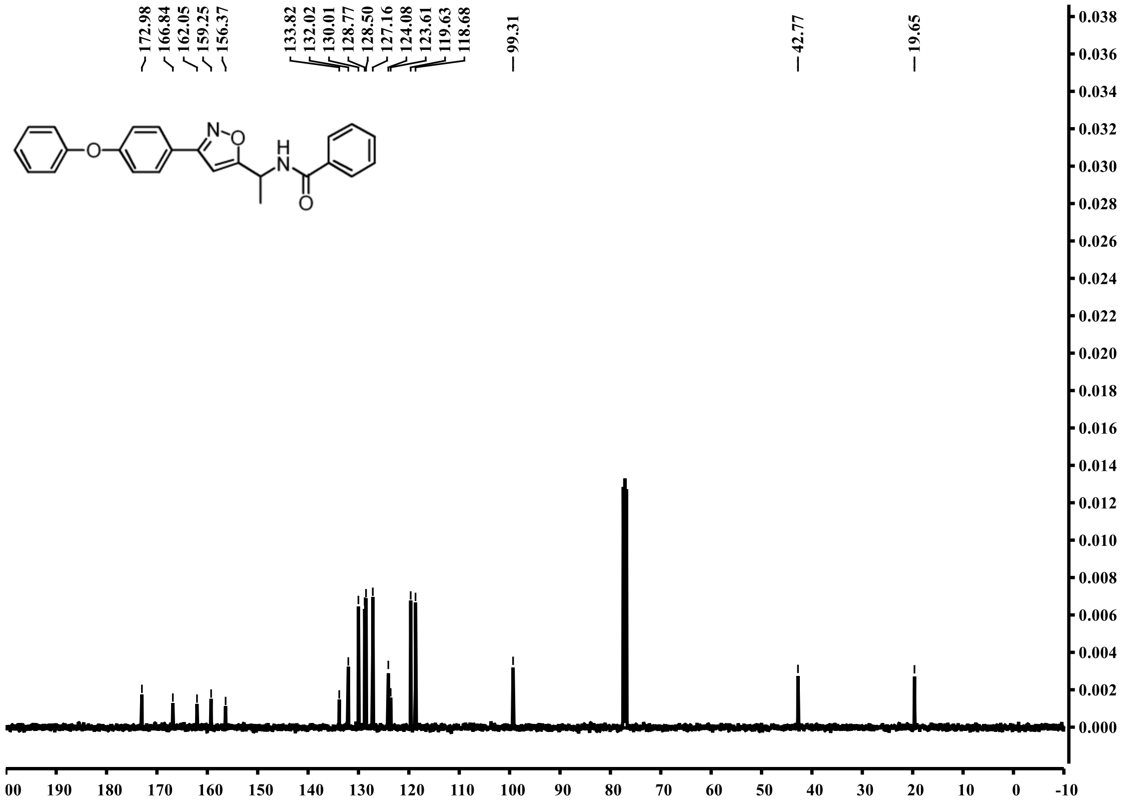

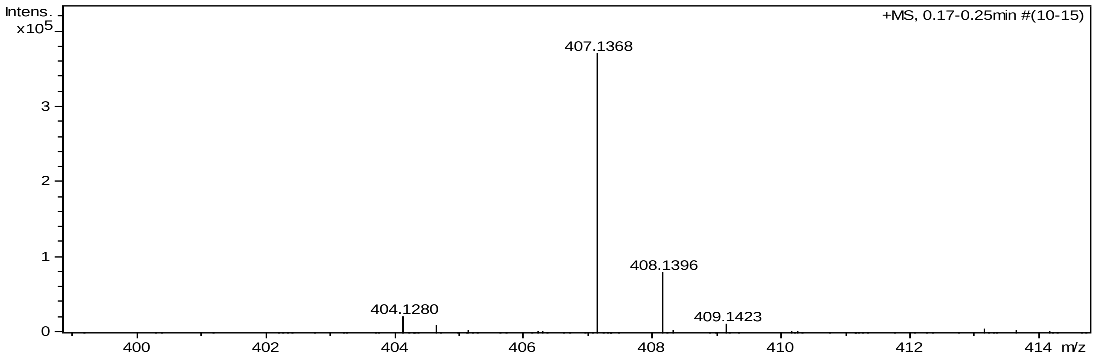

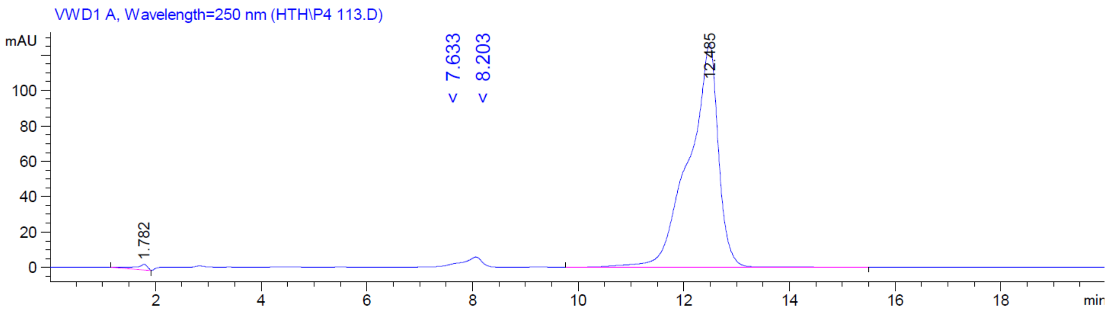

峰 #	保留时间 [min]	类型	峰宽 [min]	峰面积 [mAU*s]	峰高 [mAU]	峰面积 %
1	1.782	BB	0.2248	50.06017	3.09345	1.0079
2	12.485	VV R	0.5256	4916.75244	126.44912	98.9921

*N-(1-(3-(4-phenoxyphenyl)isoxazol-5-yl)ethyl)-2-phenylacetamide (**6l**).*

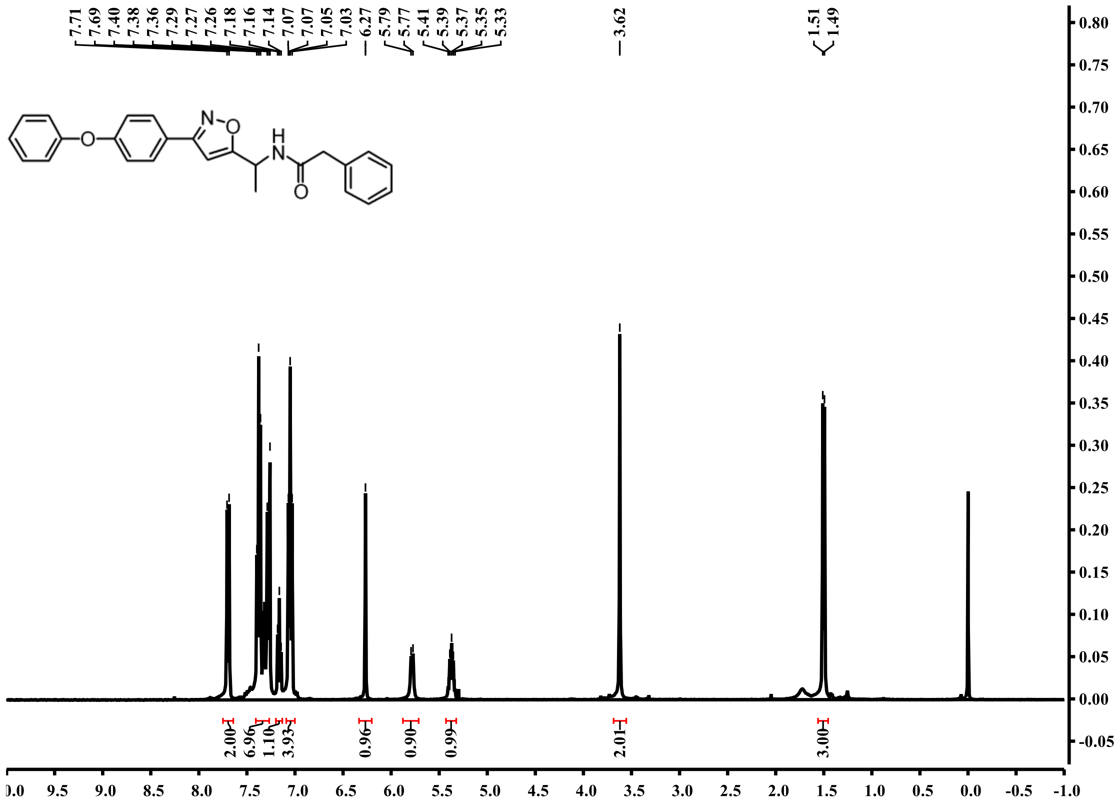

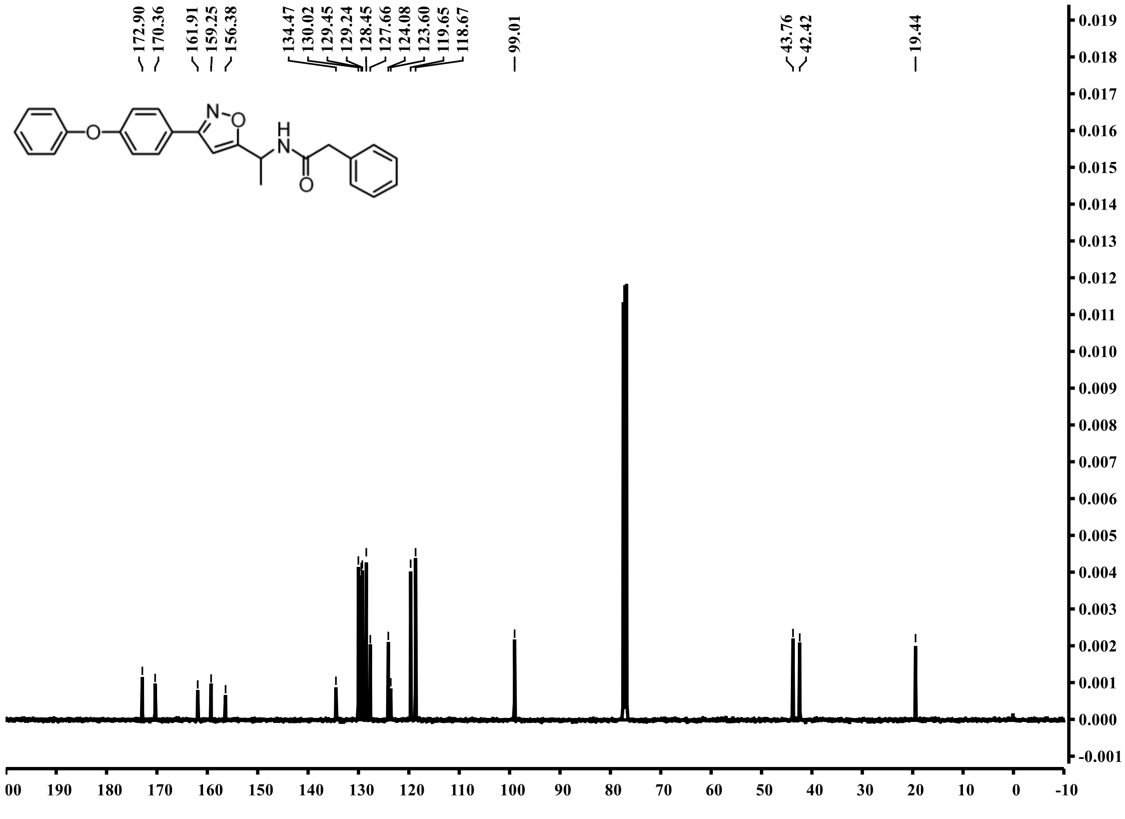

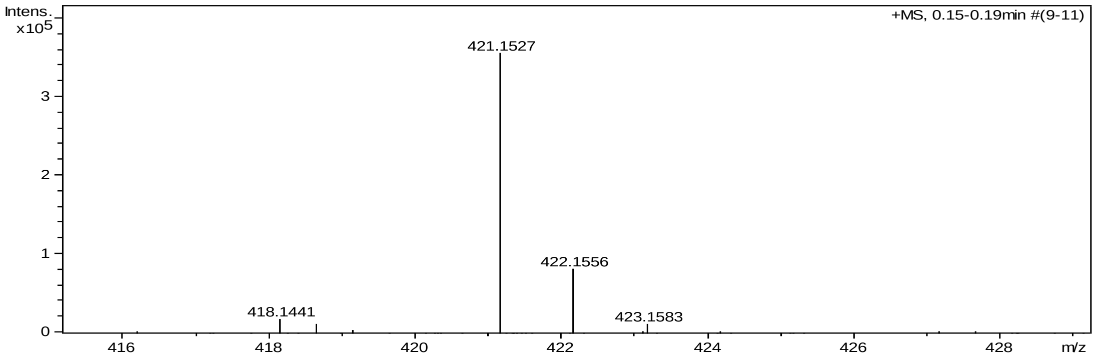

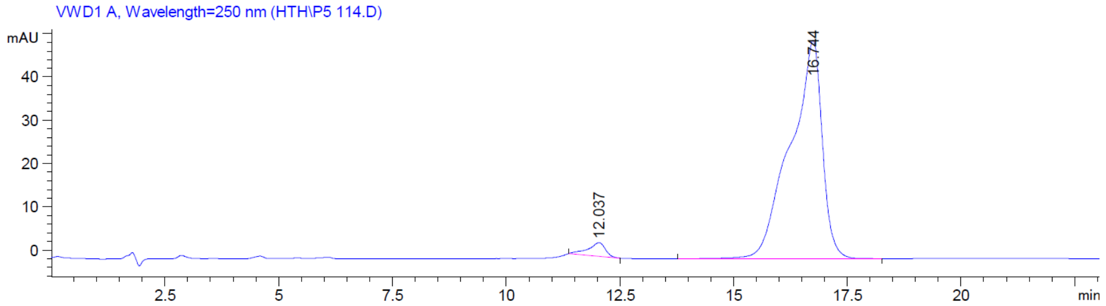

峰 #	保留时间 [min]	类型	峰宽 [min]	峰面积 [mAU*s]	峰高 [mAU]	峰面积 %
1	12.037	BB	0.3805	82.73019	3.12908	3.2632
2	16.744	BB	0.6641	2452.53198	50.36557	96.7368

ethyl (1-(3-(4-phenoxyphenyl)isoxazol-5-yl)ethyl)carbamate ***(6m)***.

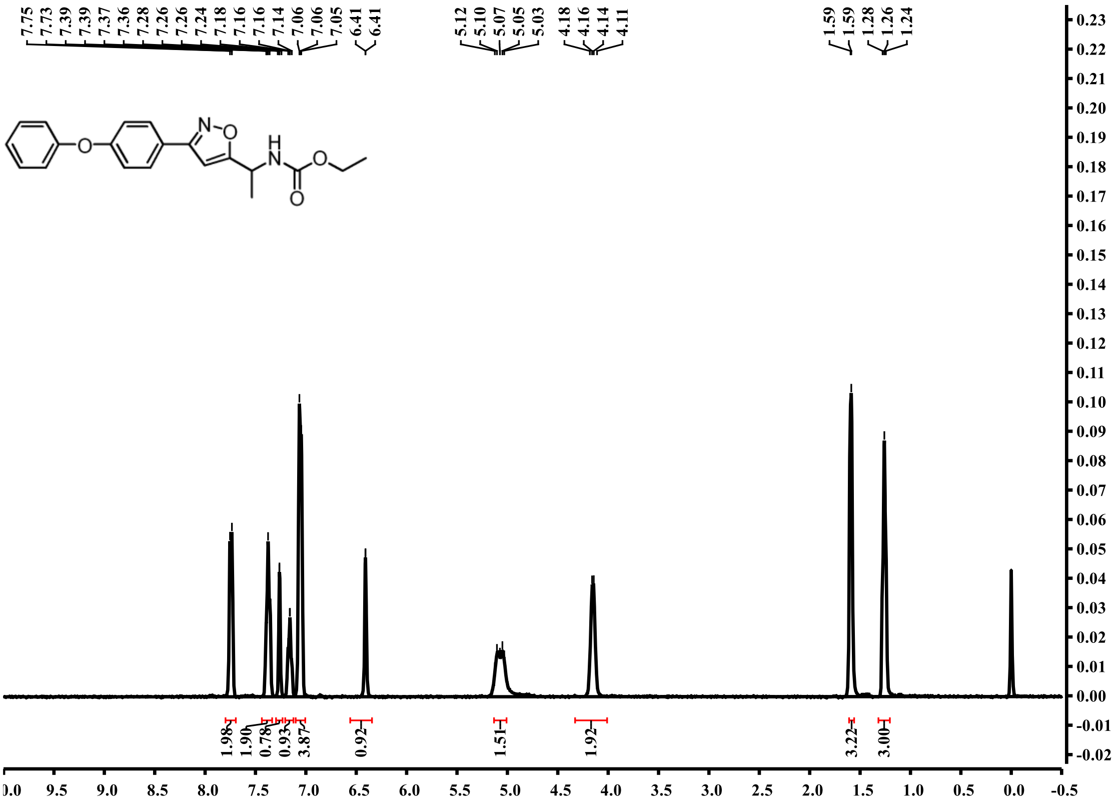

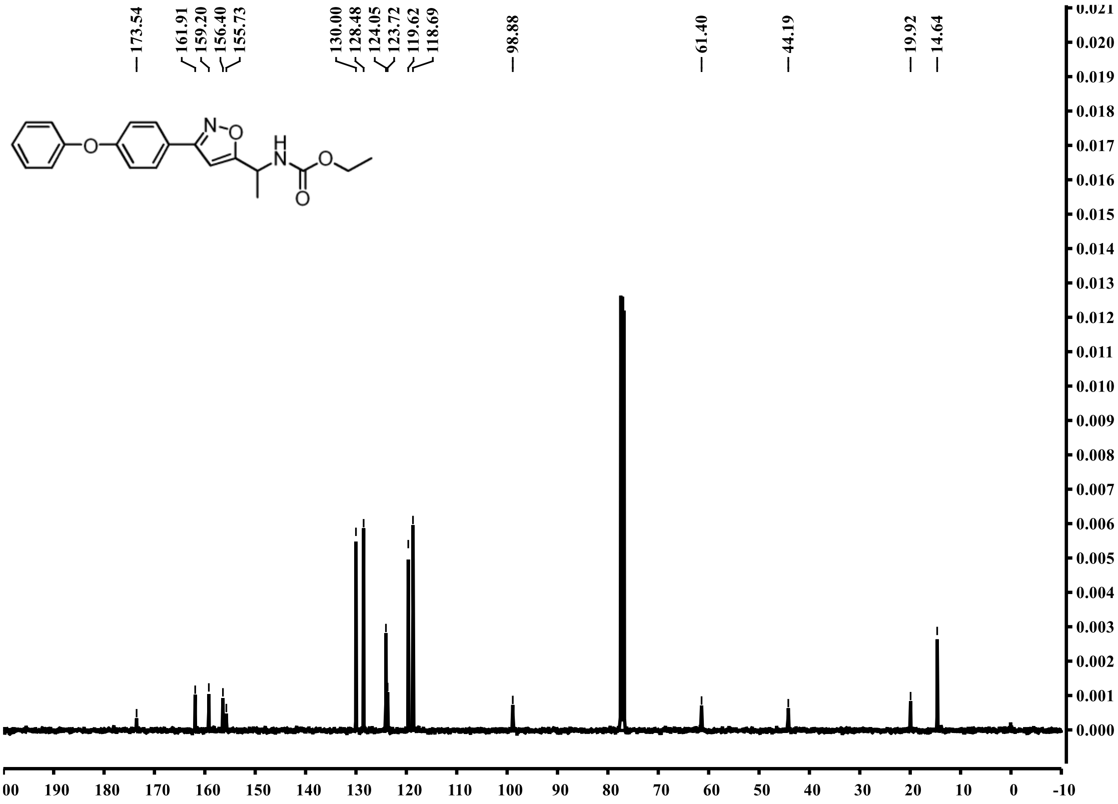

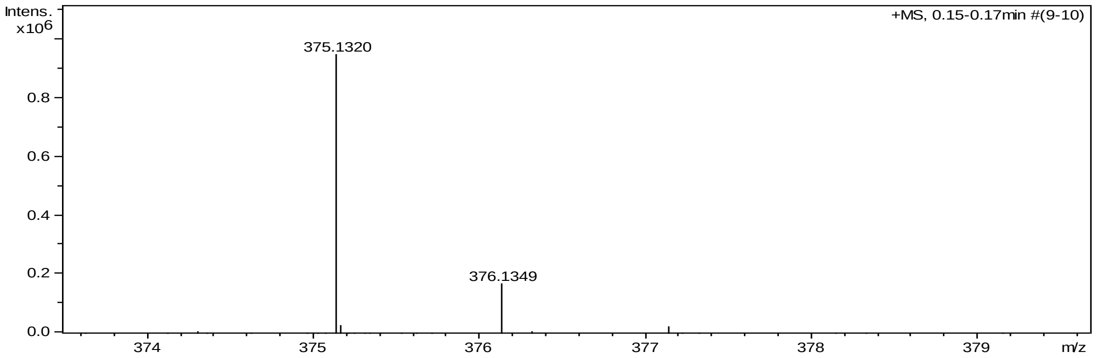

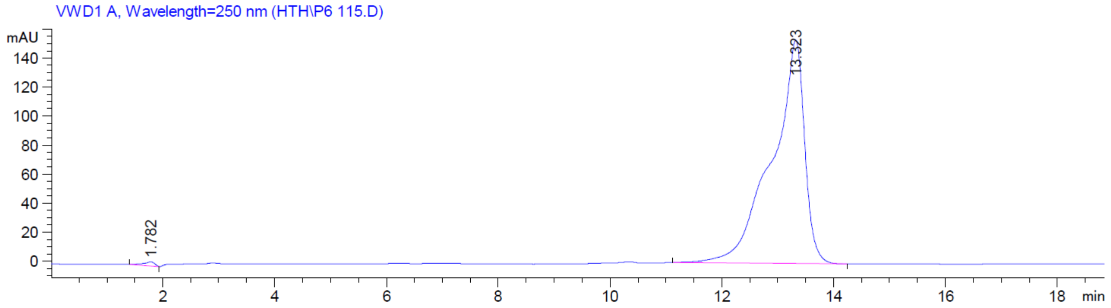

峰 #	保留时间 [min]	类型	峰宽 [min]	峰面积 [mAU*s]	峰高 [mAU]	峰面积 %
1	1.782	BB	0.1897	36.94556	2.84190	0.5888
2	13.323	BB	0.5468	6237.29639	153.89369	99.4112

*1-ethyl-3-(1-(3-(4-phenoxyphenyl)isoxazol-5-yl)ethyl)urea (**6n**).*

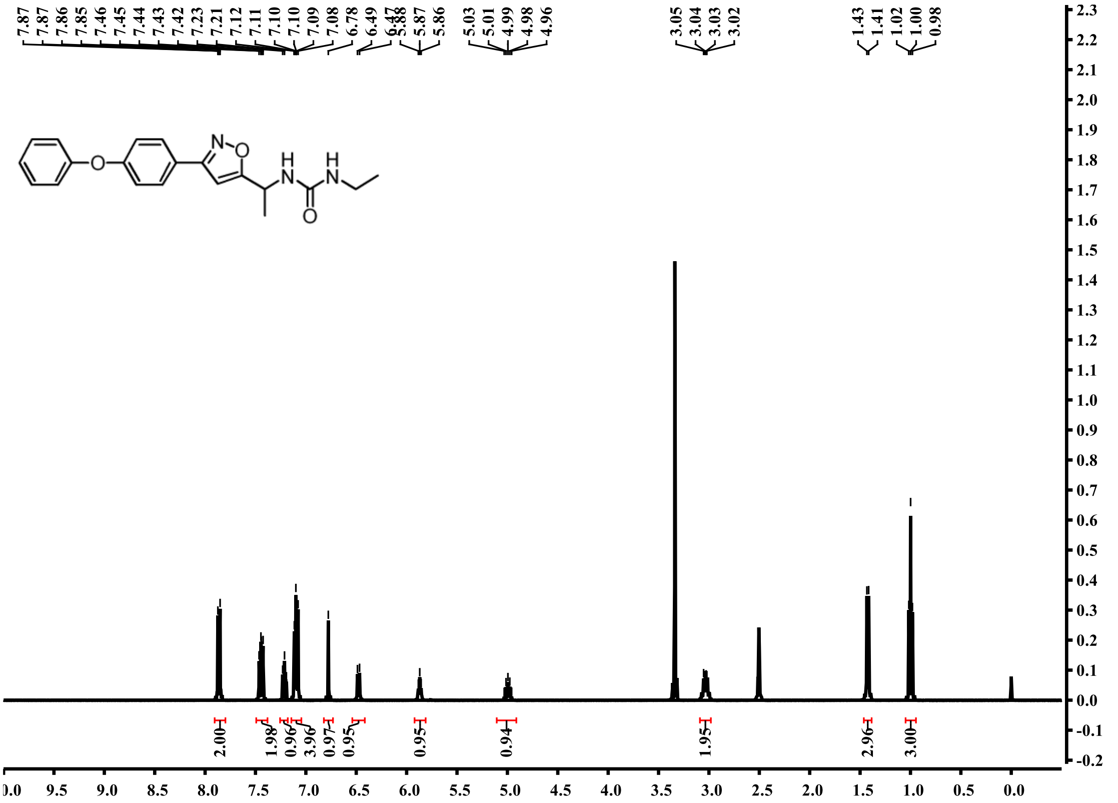

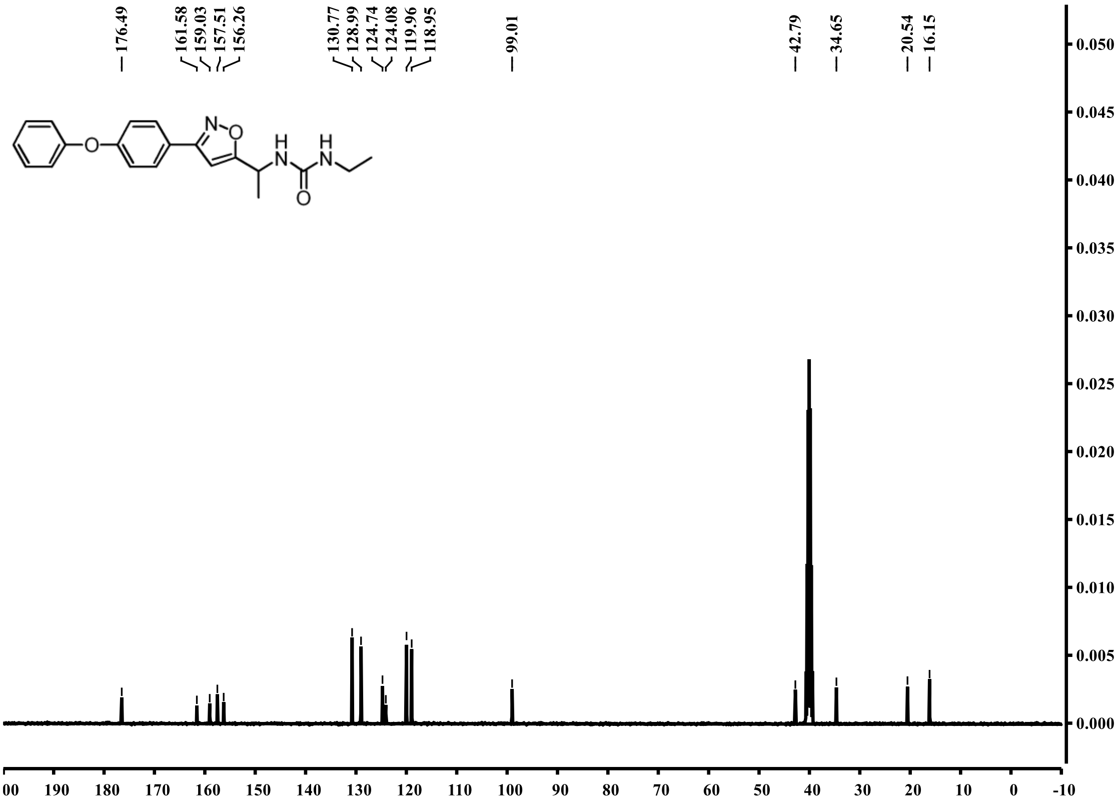

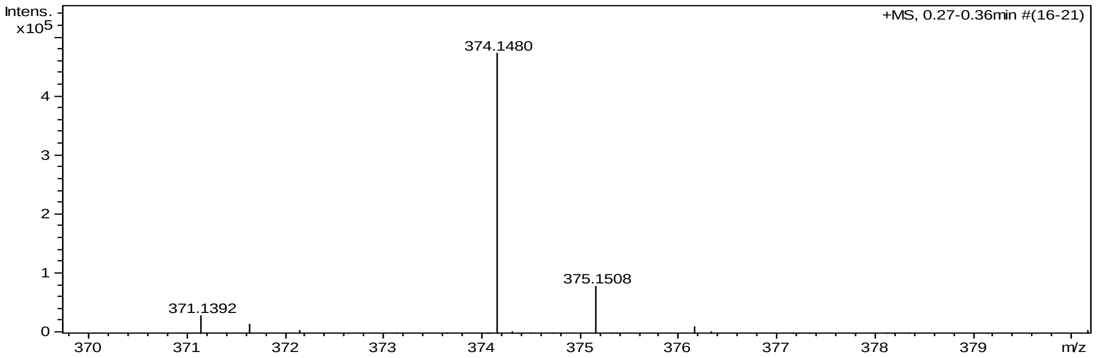

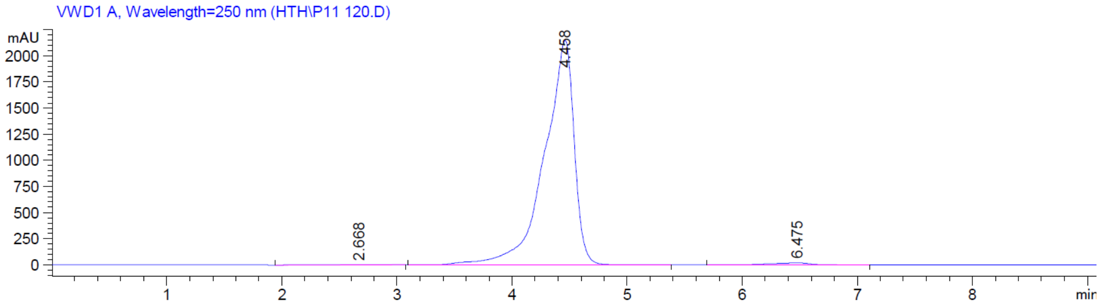

峰 #	保留时间 [min]	类型	峰宽 [min]	峰面积 [mAU*s]	峰高 [mAU]	峰面积 %
1	2.668	BB	0.3100	100.79556	4.29137	0.2572
2	4.458	BB	0.2476	3.86411e4	2150.64160	98.5845
3	6.475	BB	0.3242	454.03702	19.60263	1.1584

*1-isopropyl-3-(1-(3-(4-phenoxyphenyl)isoxazol-5-yl)ethyl)urea (**6o**).*

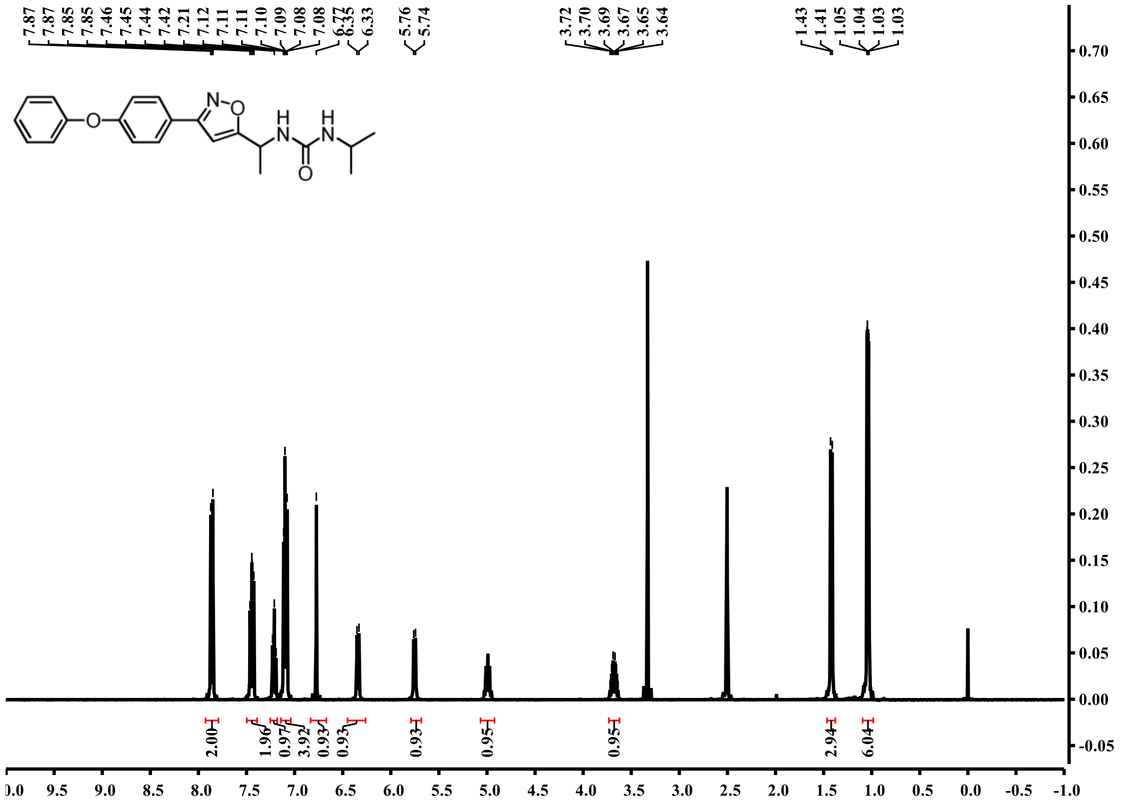

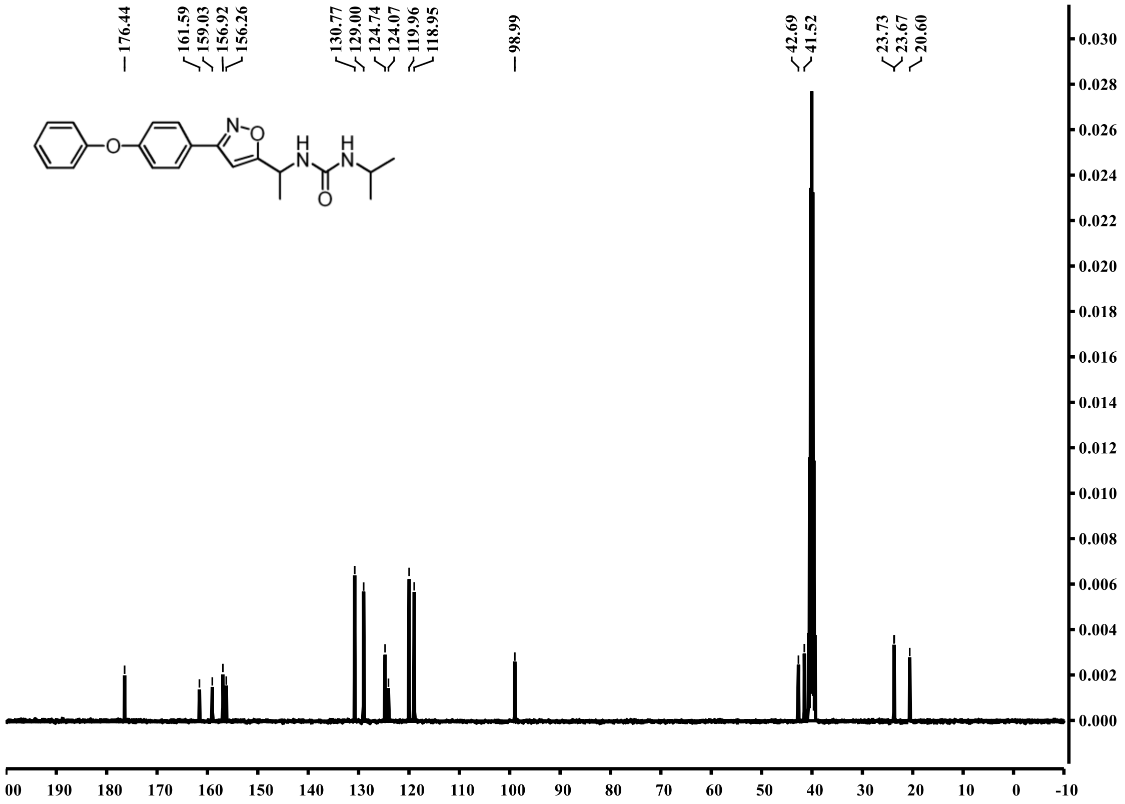

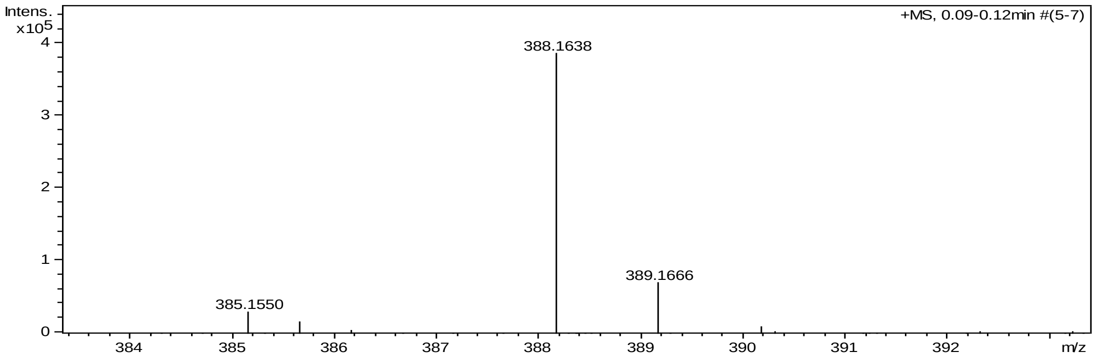

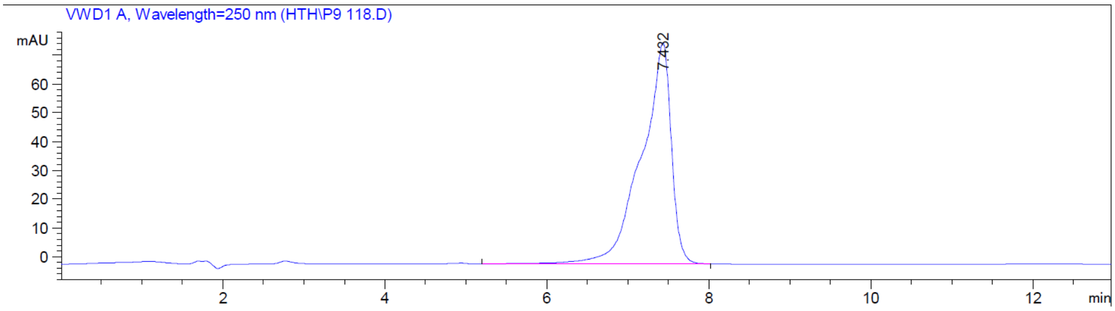

峰 #	保留时间 [min]	类型	峰宽 [min]	峰面积 [mAU*s]	峰高 [mAU]	峰面积 %
1	7.432	BB	0.3496	1971.19031	76.75361	100.0000

*1-(1-(3-(4-phenoxyphenyl)isoxazol-5-yl)ethyl)-3-phenylurea (**6p**).*

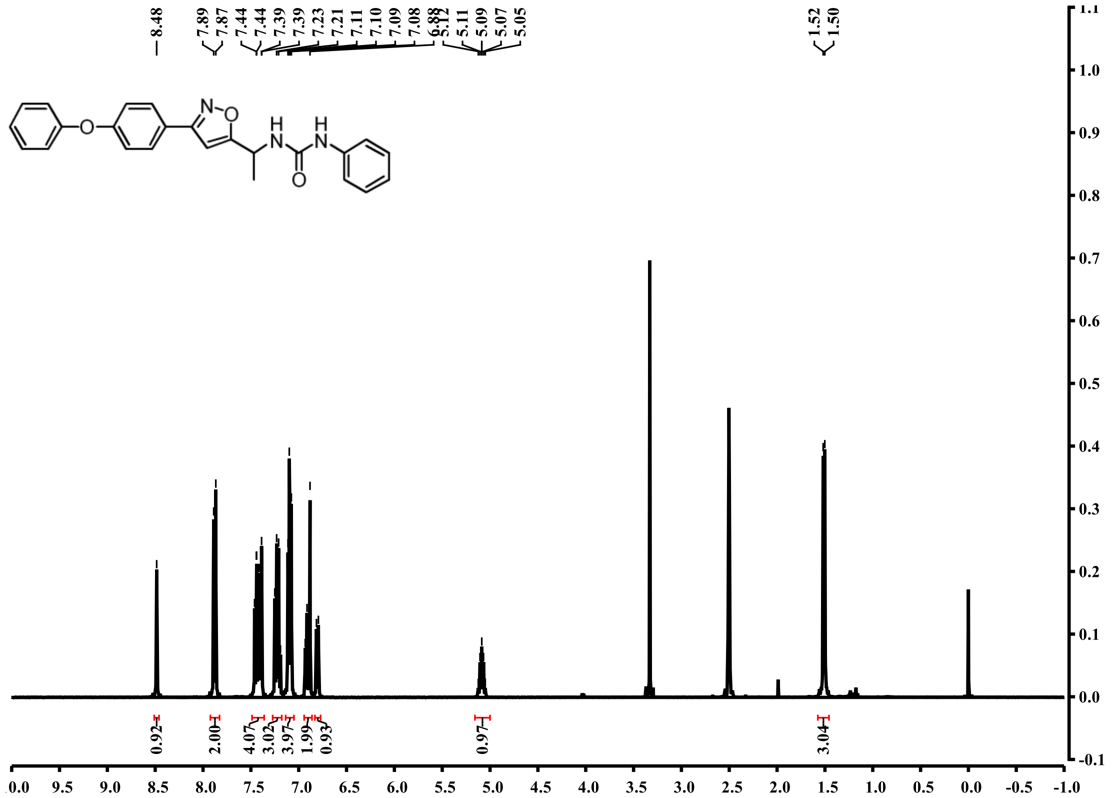

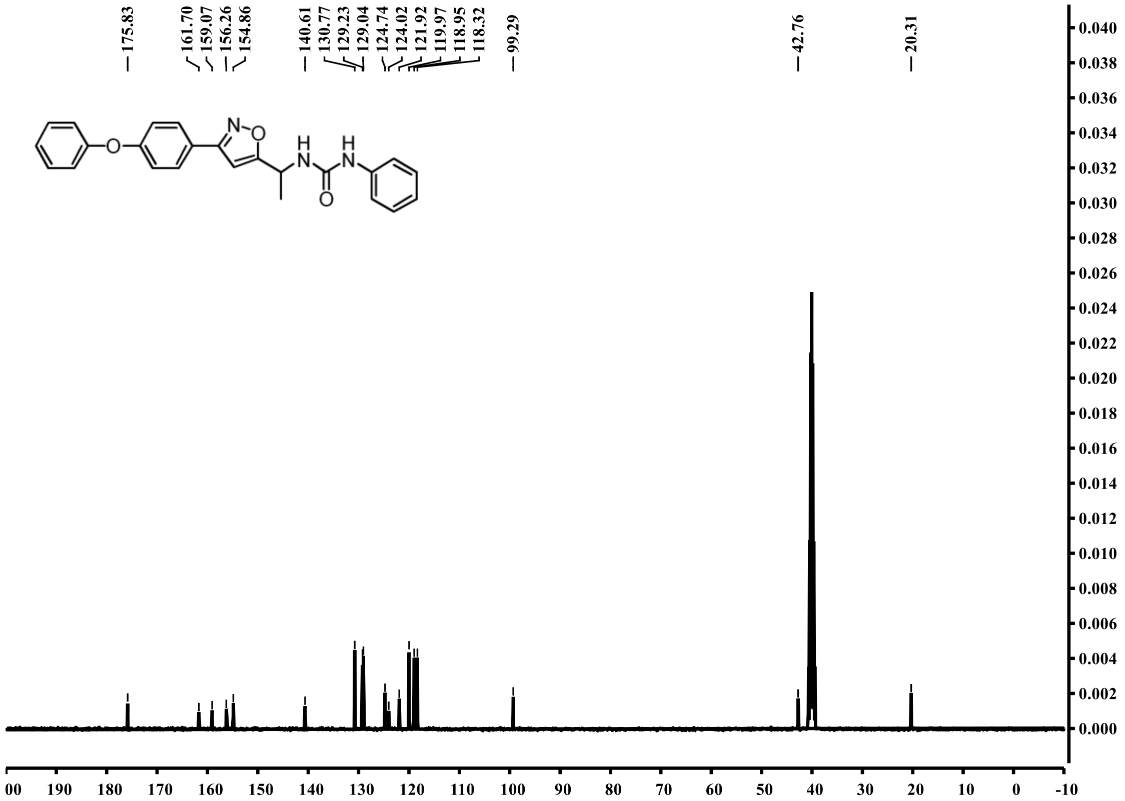

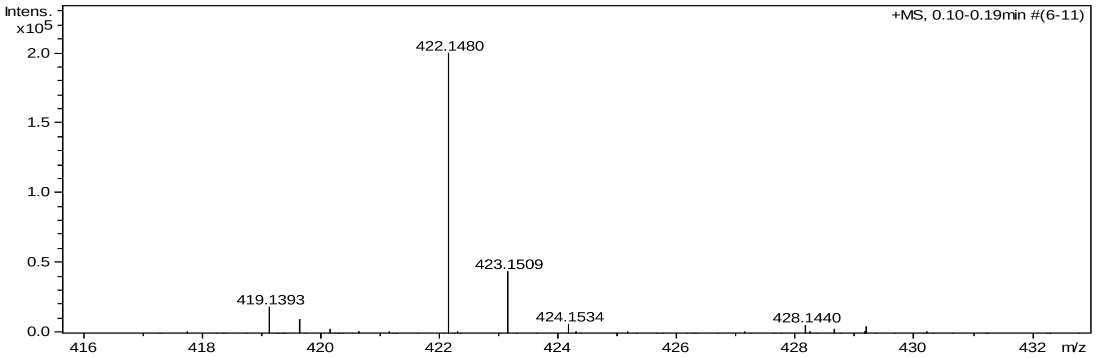

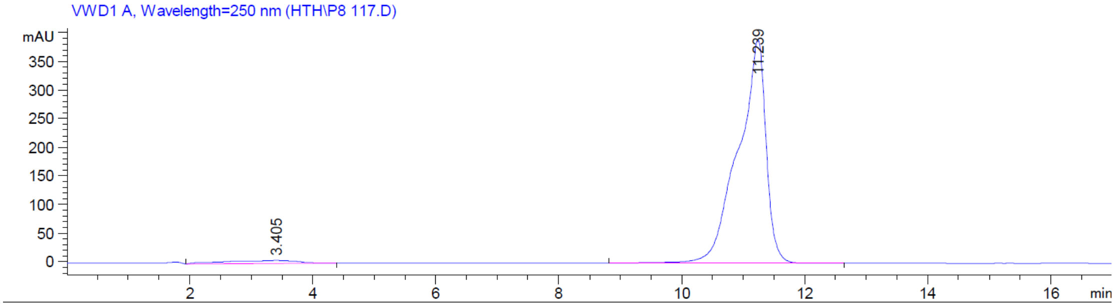

峰 #	保留时间 [min]	类型	峰宽 [min]	峰面积 [mAU*s]	峰高 [mAU]	峰面积 %
1	3.405	BB	1.0428	422.71130	5.25329	3.3179
2	11.239	BB	0.4274	1.23175e4	391.12131	96.6821

*1-benzyl-3-(1-(3-(4-phenoxyphenyl)isoxazol-5-yl)ethyl)urea (**6q**).*

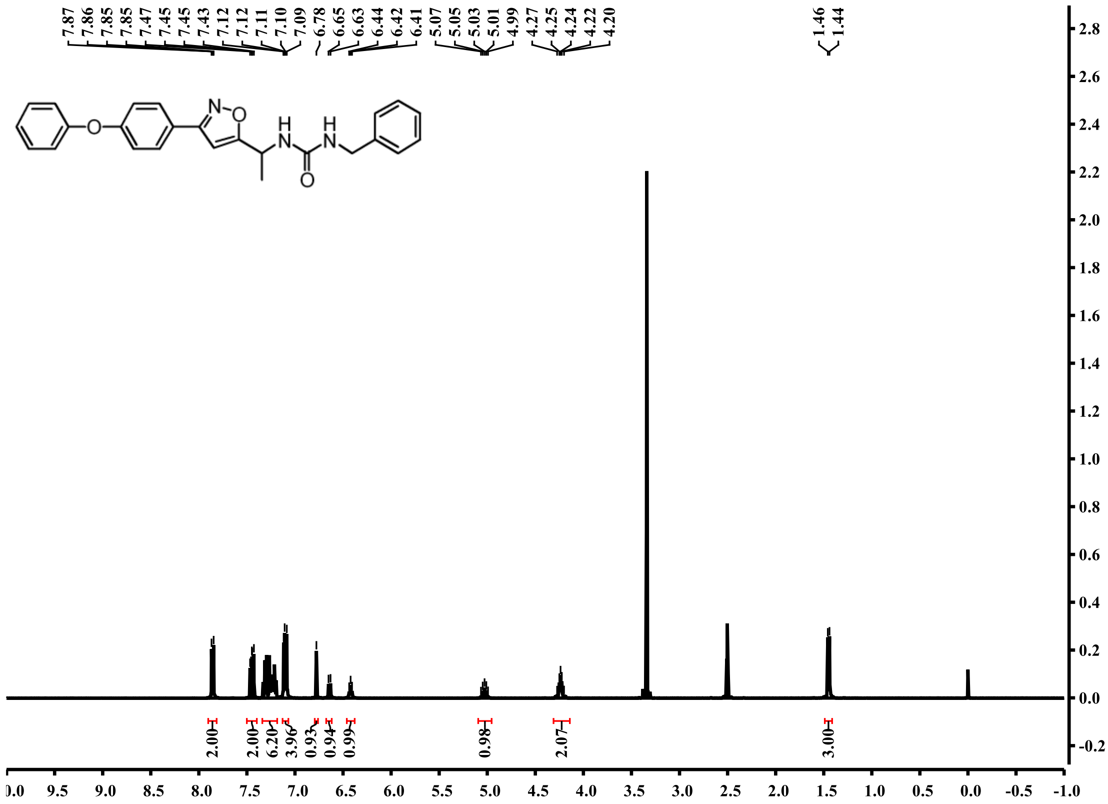

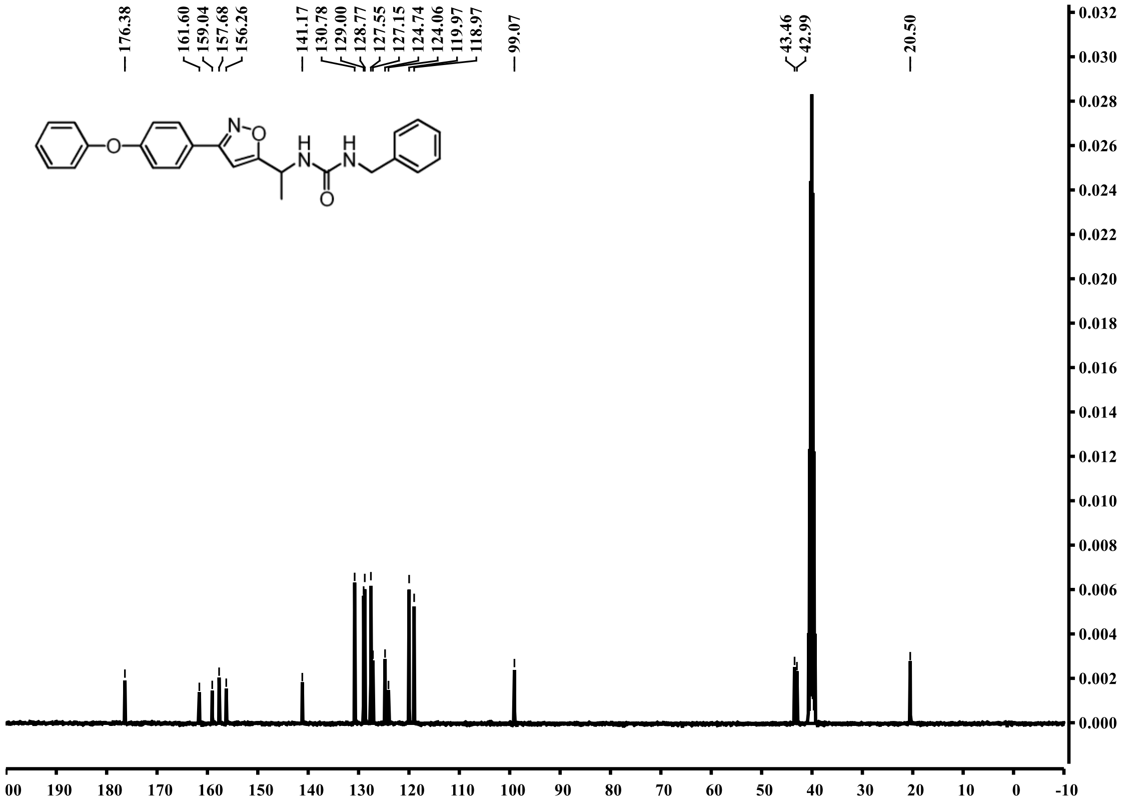

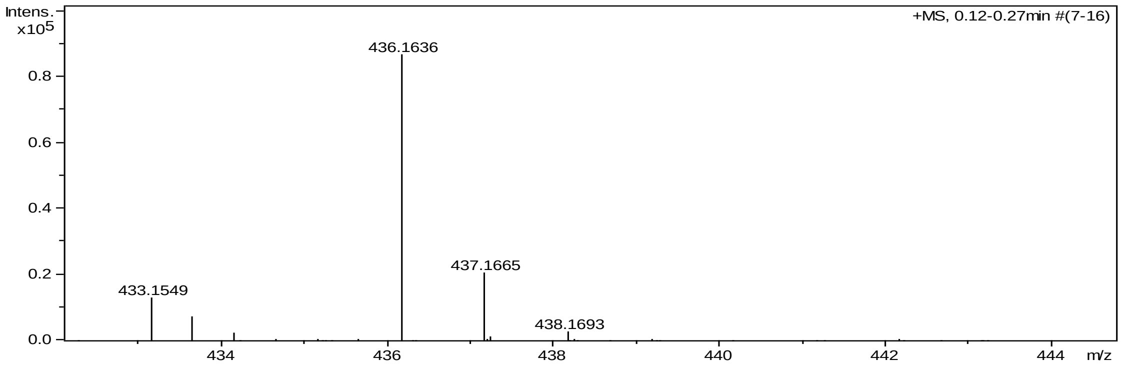

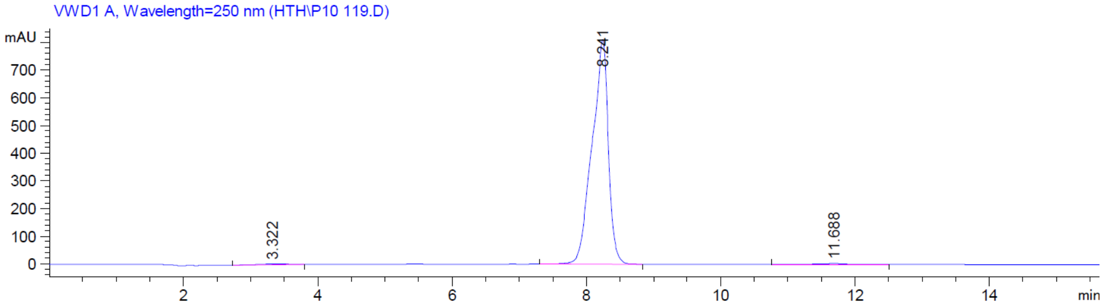

峰 #	保留时间 [min]	类型	峰宽 [min]	峰面积 [mAU*s]	峰高 [mAU]	峰面积 %
1	3.322	BB	0.3438	93.87563	3.65273	0.6808
2	8.241	BB	0.2339	1.36341e4	811.93140	98.8804
3	11.688	BB	0.3137	60.49813	2.69662	0.4388

Abbreviation list

Full name	Abbreviation
Acetyl-CoA carboxylase	ACC
high-throughput virtual screening	HTVS
structure-activity relationship	SAR
fatty acid synthesis	FASyn
fatty acid oxidation	FAOxn
carnitine palmitoyltransferase I	CPT-1
type 2 diabetes mellitus	T2DM
nonalcoholic steatohepatitis	NASH
N-chlorosuccinimide	NCS
inhibition rates	IRs
doxorubicin	DOX
calculated logarithm of the octanol-water partition coefficient	cLogP
carboxyltransferase	CT
